# Antibody‒drug conjugates with DHODH inhibitor as novel payload class for cancer and SARS-CoV-2 infection therapies

**DOI:** 10.1016/j.apsb.2025.11.008

**Published:** 2025-11-12

**Authors:** Zhirui Liu, Lunzhi Yuan, Pengyun Li, Fei Xie, Ming Zhou, Lianqi Liu, Ting Wei, Yi Guan, Ningshao Xia, Zhibing Zheng, Tong Cheng, Dian Xiao, Xinbo Zhou, Song Li

**Affiliations:** a School of Pharmaceutical Engineering, Shenyang Pharmaceutical University, Shenyang 110016, China; b National Engineering Research Center for the Emergency Drug, Beijing Institute of Pharmacology and Toxicology, Beijing 100850, China; c State Key Laboratory of Molecular Vaccinology and Molecular Diagnostics, National Institute of Diagnostics and Vaccine Development in Infectious Diseases School of Life Sciences, School of Public Health, Xiamen University, Xiamen 361000, China; d State Key Laboratory of Emerging Infectious Diseases, School of Public Health, Li Ka Shing Faculty of Medicine, The University of Hong Kong, Hong Kong 999077, China; e Guangdong-Hong Kong Joint Laboratory of Emerging Infectious Diseases, Joint Laboratory for International Collaboration in Virology and Emerging Infectious Diseases, Joint Institute of Virology (STU/HKU), Shantou University, Shantou 515000, China

**Keywords:** DHODH inhibitor, Antibody‒drug conjugates, Novel payload, Broad-spectrum, Antitumor, Antiviral, Ferroptosis, Drug combination

## Abstract

Despite remarkable achievements in antibody‒drug conjugates (ADCs), payloads remain limited. The identification of ADC payloads with novel mechanisms will increase therapeutic options and expand indications. Herein, we describe the use of dihydroorotate dehydrogenase inhibitors (DHODHi) as a novel payload class that provides highly potent ADCs for antitumor and antiviral therapies. Technical innovations include the development of stability-controllable linkers to meet the distinct requirements of acute viral infections and chronic tumor conditions. The antitumor ADC **TH-C8H** exhibited significant efficacy against gastric cancer *in vivo* as monotherapy and enhanced efficacy when combined with the ferroptosis inducer **RSL3**. The antiviral ADC **HG-C3** showed broad-spectrum anti-SARS-CoV-2 activity *in vitro* and *in vivo*. Our study expands the types of ADC payloads and provides novel insights into the development of innovative broad-spectrum ADCs.

## Graphical abstract

Novel DHODHi-based ADCs exhibited broad-spectrum antitumor and antiviral activities by blocking DNA/RNA replication. And the innovation of stability-controllable linkers promoted this discovery.

**Figure:**
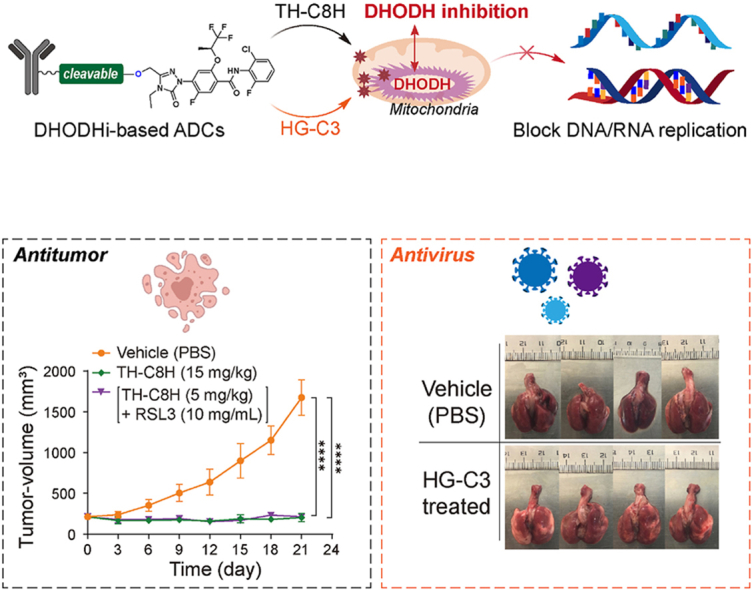

## 1 Introduction

Antibody‒drug conjugates (ADCs), which consist of a cytotoxic payload conjugated to a monoclonal antibody *via* a chemical linker, have garnered great clinical success. Exhibiting advantages over traditional chemotherapy, ADCs offer superior targeting capabilities, fewer side effects, and a higher therapeutic index^1^.

**Figure 1:**
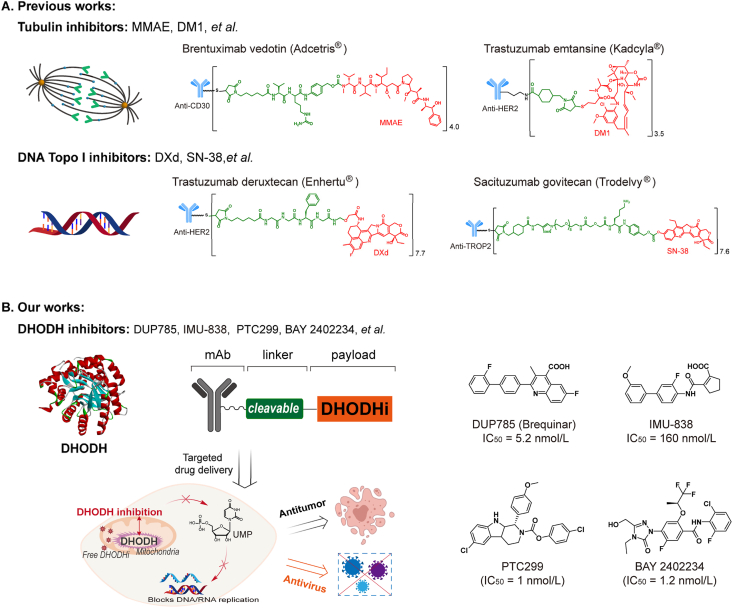
Design of novel DHODHi-based ADCs. (A) Tubulin inhibitors and DNA Topo I inhibitors dominate traditional ADC payloads. (B) Novel DHODHi-based ADCs contribute to the development of broad-spectrum ADCs for antitumor and antiviral infections.

The payload is a crucial component of an ADC that potentiates its therapeutic action. As ADCs can only carry limited amounts of payload to tumor tissues, the payload must be extremely cytotoxic^2^^,^^3^. Currently, the IC_50_ values of payloads are typically as low as 10^−12^–10^−10^ mol/L^4^. Consequently, the categories of payloads employed in the launched ADCs are limited, predominantly comprising tubulin inhibitors (*e.g.*, **MMAE**) and DNA topoisomerase I inhibitors (*e.g.*, **DXd**)^4^^,^^5^ (Fig. 1A). Research on other potential payloads, such as RNA splicing inhibitors, DNA topoisomerase II inhibitors, and STING agonists is ongoing^3^. In addition, the applications of most payloads are currently restricted to cancer therapy^2^^,^^3^^,^^6^, underlining the crucial need for developing payloads with novel mechanisms^7^. Discovery of novel payloads could contribute to overcoming resistance, improving the therapeutic index and expanding indications in the future^7^, ^8^, ^9^.

DHODH is a pivotal rate-limiting enzyme in the *de novo* pyrimidine synthesis pathway by converting dihydroorotate (DHO) to orotate^10^. DHODH inhibition blocks intracellular pyrimidine synthesis and interferes with DNA/RNA replication (Fig. 1B). Rapid proliferative nature of tumors and viruses makes DHODH an ideal therapeutic target^10^, ^11^, ^12^, ^13^. Recent studies have reported the effectiveness of DHODH inhibitors (DHODHi) against tumors and viral infections. For instance, Leflunomide, **PTC299**, **BAY 2402234**, Brequinar, **AG-636**, and **ASLAN003** exhibit high antitumor activity^10^^,^^14^, ^15^, ^16^, ^17^, ^18^, ^19^, ^20^, ^21^, whereas Brequinar and Teriflunomide are effective against SARS-CoV-2, influenza A, Zika, and Ebola^10^^,^^22^. **PTC299** and **IMU-838** also exhibit SARS-CoV-2 inhibitory activity^13^^,^^23^. Additionally, **Brequinar** has been shown to induce ferroptosis in GPX4^low^ tumor cells when synergized with ferroptosis inducers^24^. DHODH inhibition induces ferroptosis by elevating intracellular peroxide levels in tumor cells^11^^,^^25^, ^26^, ^27^. Therefore, a combination of DHODH inhibitors and ferroptosis inducers may contribute to enhanced antitumor activity. However, systemic distribution resulting from direct delivery limits the availability of DHODHis^28^. Therefore, achieving targeted delivery of DHODHis is a crucial research topic that demonstrates important application value.

In this study, we successfully developed ADCs using DHODHi as a novel payload and explored their antitumor and antiviral infection efficacies (Fig. 1B). And series of stability-controllable linkers were explored to meet the distinct requirements of acute viral infections and chronic tumor conditions. To explore the antitumor potential of **TH-C8H**, we conducted multiple exploratory experiments, including cell proliferation assay, cell cycle arrest assay, apoptosis analysis, and tumor growth inhibition assay in a xenograft model. To explore the potential of **HG-C3** against viral infections, we performed viral proliferation inhibition tests on various SARS-CoV-2 mutant strains, including the prototype, Delta, B.A5, and XBB1.9.2.1. The data showed that **TH-C8H** displayed significant monotherapy efficacy against gastric cancer *in vivo*, and demonstrated enhanced efficacy in combination with the ferroptosis inducer **RSL3**. At the same time, **HG-C3** exhibited broad-spectrum anti-SARS-CoV-2 activities both *in vitro* and *in vivo*. Our study successfully identified a novel ADC payload that contributes to the development of broad-spectrum antitumor ADC, and provides new insights into antiviral applications.

## 2 Results

### 2.1 Design and structural optimization of DHODHi-based ADCs

**Figure 2:**
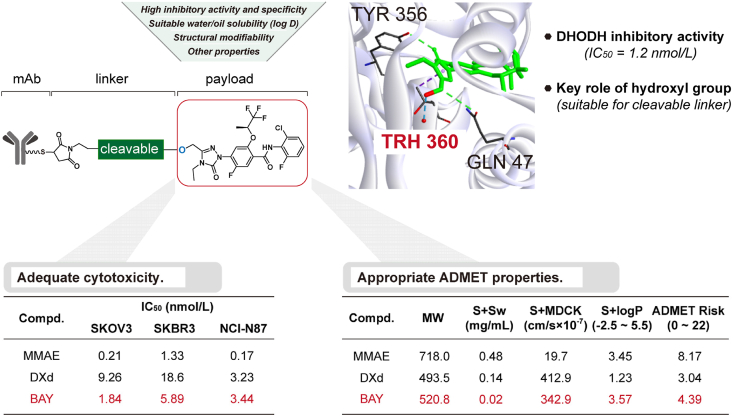
DHODHi **BAY** is selected as a candidate payload based on structural modifiability, adequate cytotoxicity and appropriate ADMET properties.

The payload, a key component of ADC, demands stringent requirements for high cytotoxicity, adequate stability, suitable water solubility, and structural modifiability^3^^,^^6^^,^^8^. Various FDA-approved and clinically-used DHODHis have been reported including, Teriflunomide, **DUP785** (Brequinar), **IMU-838**, **PTC299**, and **BAY 2402234**, etc.^15^^,^^23^^,^^29^, ^30^, ^31^. We ultimately selected **BAY 2402234** (**BAY**) as the candidate payload (Fig. 2) primarily because of its high inhibitory activity against DHODH (IC_50_ = 1.2 nmol/L)^15^. Cytotoxicity test data showed that the IC_50_ values of **BAY** in different tumor cells ranged from 1.84 to 5.89 nmol/L, generally stronger than **DXd** while slightly weaker than **MMAE** (Fig. 2; Supporting Information Fig. S1). Its lipophilicity (log*D* = 2.7, *S* + log*P* = 3.57)^15^ and permeability (*S* + MDCK = 342.9 cm/s∗10^−7^) favor bystander killing effects. In addition, **BAY** features a modifiable hydroxyl group and appropriate metabolic half-life (*T*_1/2_ = 4.1 h)^15^. The high cytotoxicity and appropriate ADMET properties make **BAY** a promising ADC payload. However, several challenges remain. Unlike traditional payloads (**MMAE**), which are modified from the amino group^32^^,^^33^, **BAY** contains only one hydroxyl group that can be conjugated with linkers. The binding model revealed that the hydroxyl group of **BAY** interacts with Thr360 in the active pocket of DHODH (PDB 6QU7) *via* hydrogen bonding^15^^,^^34^ (Fig. 2), indicating the necessity of a cleavable linker. Therefore, we designed various cleavable linkers to construct DHODHi-based ADCs (Fig. 2).

**Figure 3:**
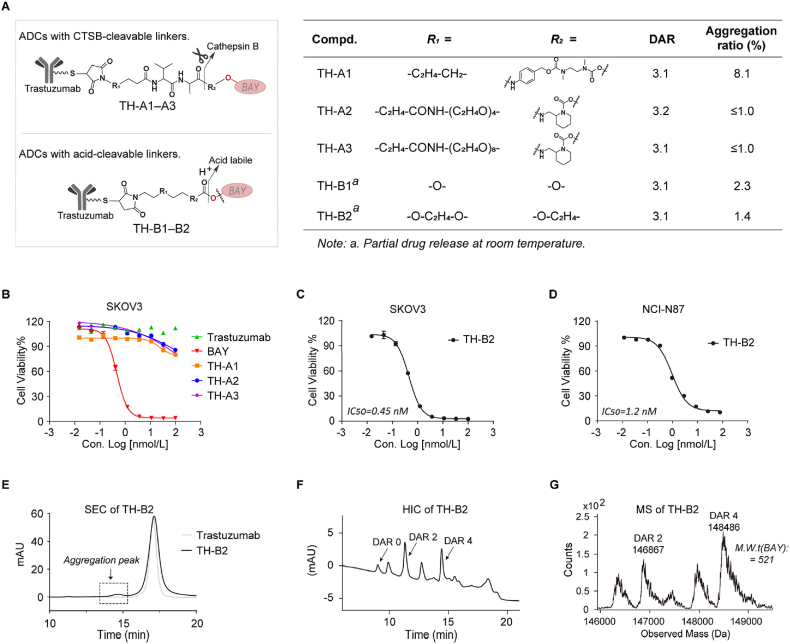
The design and activity evaluation of ADCs with CTSB-cleavable and acid-cleavable linkers. (A) The structural information and quality study of ADCs with CTSB-cleavable and acid-cleavable linkers. (B) Cytotoxicity of the CTSB-cleavable ADCs **TH-A1–A3** in SKOV3 cell. (C, D) Cytotoxicity assay of the acid-cleavable ADC **TH-B2** in SKOV3 (C) and NCI-N87 (D) cells. (E) SEC analysis for aggregation level of **TH-B2**. (F) HIC analysis for DAR of **TH-B2**. (G) MS analysis of **TH-B2**. For (B), (C), and (D), data are shown as mean ± SD (*n* ≥ 2).

ADCs featuring cathepsin B (CTSB)-cleavable linkers were designed (Fig. 3A). Linkers **A1–A3** were obtained by inserting ethylenediamine or *N-*methylpyridine between the dipeptide (Val-Ala) fragments and DHODHi **BAY** (Supporting Information Scheme S1). They were then conjugated to HER2 antigen-targeted Trastuzumab to prepare the target ADCs **TH-A1–A3** (Fig. 3A; Scheme S1). Quality analysis showed that the drug–antibody ratios (DAR) of **TH-A1–A3** were 3.1, 3.2, and 3.1 (Supporting Information Fig. S2A), and the aggregation ratios were 8.1%, 1.0%, and 1.0% (Fig. S2B). However, in the HER2^+^ SKOV3 cell lines, the IC_50_ values of **TH-A1–A3** were above 100 nmol/L (Fig. 3B). These data suggested that the dipeptide derivative linkers were too stable to release **BAY**. Subsequently, the acid-cleavable linkers **B1–B2** were designed (Fig. 3A). Carboxylate and carbonate-based linkers were synthesized and conjugated to Trastuzumab to prepare the target ADCs **TH-B1–B2** (Supporting Information Scheme S2). Testing data showed that **TH-B2** exhibited high cytotoxicity against HER2^+^ SKOV3 and NCI-N87 cell lines, with IC_50_ values of 0.45 and 1.2 nmol/L, respectively (Fig. 3C and D). Size exclusion chromatography (SEC), hydrophobic interaction chromatography (HIC), and mass spectrometry (MS) analyses showed that **TH-B2** generally met the quality requirements, except for a few unwanted drug shedding signals (Fig. 3E‒G; Fig. S2C and S2D).

**Figure 4:**
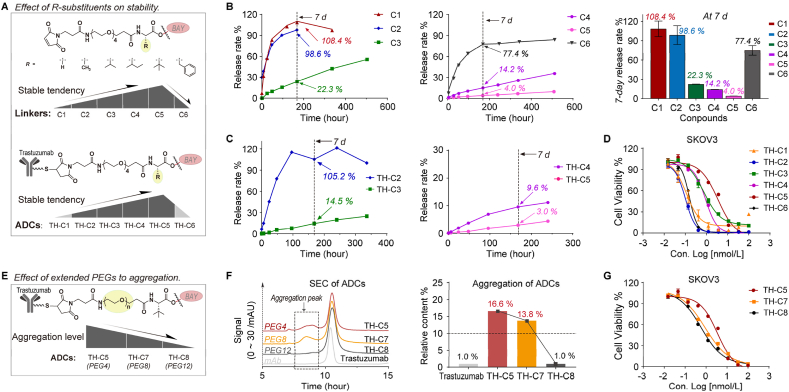
The design, stability study and evaluation of ADCs with stability-controllable linkers. (A) Structural information of linkers (**C1–C6**) and ADCs (**TH-C1–C6**). (B) Stability study of linkers. (C) Stability study of ADCs. (D) Cytotoxicity of ADCs (**TH-C1–C6**) in SKOV3 cells. (E) Extended PEGs contributed to lower the aggregation level of ADCs. (F) SEC analysis for aggregation level of ADCs (**TH-C5**, **TH-C7**, and **TH-C8**) with different PEGs. (G) Cytotoxicity of ADCs (**TH-C5**, **TH-C7**, and **TH-C8**) in SKOV3 cells. For (B), (C), (D), and (G), data are shown as mean ± SD (*n* ≥ 2).

Inspired by the antitumor effects of ADC with acid-cleavable linkers, we explored ADCs with more stable and controllable linkers. Specifically, the *α*-amino acid ester bond was designed as the cleavable trigger of the linker, and substituents (R-groups) with different spatial sizes were introduced around it to regulate the stability. By applying this strategy, linkers **C1**–**C6** were designed, synthesized, and conjugated to Trastuzumab to prepare the target ADCs **TH-C1**–**C6** (Fig. 4A; Supporting Information Scheme S3). Data from stability studies of linkers **C1**–**C6** supported this strategy. By Day 7, the release percentages of drugs from the PBS solution of **C1**–**C5** were respectively 108.4%, 98.6%, 22.3%, 14.2%, and 4.0% (Fig. 4B), demonstrating that linker stability increased with steric hindrance at the *α*-position of the carbonyl carbon atom. However, compound **C6**, possessing a “large” benzyl R-substituent, showed significantly lower stability, with a 7-day release rate of 77.4% (Fig. 4B). This anomaly may be due to the planar structure of the sp2 hybridized orbitals of benzene ring restricting the steric hindrance effect. Moreover, plasma stability studies of the ADCs revealed that **TH-C2**–**C5** exhibited drug release rates of 105.2%, 14.5%, 9.6%, and 3.0%, by Day 7 (Fig. 4C), respectively, which was consistent with the stability trend of the linkers (Supporting Information Fig. S3A). These results indicated that plasma does not significantly accelerate drug release, and ADCs maintain a controllable stability in systemic circulation. The cytotoxicity data showed that ADCs based on this strategy maintained satisfactory activity. In the SKOV3 cell line, the IC_50_ values of **TH-C1**–**C6** were 0.11, 0.10, 0.75, 0.89, 3.5, and 0.18 nmol/L (Fig. 4D), respectively.

We then performed a qualitative analysis of the ADCs. The data showed that the DARs of **TH-C1**–**C6** were 3.1, 2.7, 3.2, 2.4, 2.8, and 1.6 (Fig. S3B), with aggregation ratios of 3.1%, 7.9%, 3.0%, 24.2%, 16.6%, and 55.8%, respectively (Fig. S3C). Notably, the aggregation ratios of **TH-C4**–**6** exceeded the standard (≤10.0%), commonly caused by poor water solubility of the payload. Excess aggregation increased the risk of rapid clearance *in vivo*. Therefore, we lengthened the polyethylene glycol (PEG) chain of linker **C5** (PEG4) to obtain linkers **C7** (PEG8) and **C8** (PEG12) and then conjugated them with Trastuzumab to prepare ADCs **TH-C7** and **TH-C8** (Fig. 4E; Scheme S3), with aggregation ratios of 13.8% and 1.0% (Fig. 4F; Fig. S3D), respectively. The aggregation level of **TH-C8** satisfied the requirements. Cytotoxicity test data showed that the IC_50_ values of **TH-C7** and **TH-C8** were 0.89 and 0.59 nmol/L, respectively, in SKOV3 cells (Fig. 4G). The extended polyethylene glycol chain (PEGs) did not significantly affect the ADC activity. Structure-activity relationship (SAR) of stability-controllable ADCs is summarized as follows: (a) The linker modification strategy mediated by the steric hindrance effect favored stability regulation; the greater the spatial site resistance of the substituent, the higher the stability of the linker. (b) The extended PEGs contributed to a lower aggregation level of ADCs, thus favoring further enhancement of ADC activity by raising the DAR value. Overall, the stability-controlled linker modification strategy met our expectations and achieved the goal of improving the ADC stability while maintaining high activity. Subsequently, we investigated the potential of DHODHi-based ADCs for antitumor and antiviral applications.

### 2.2 Antitumor ADC **TH-C8H** exerted high cytotoxicity *in vitro*

**Figure 5:**
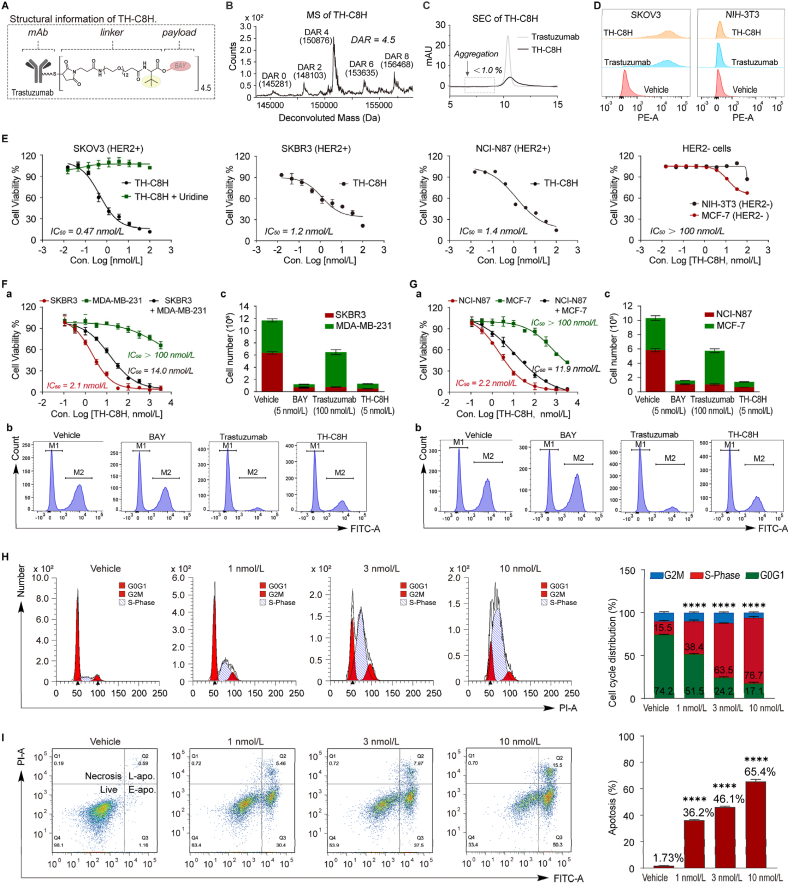
**TH-C8H** showed high antitumor activity *in vitro*. (A) Structural information of **TH-C8H**. (B) MS analysis for DAR. (C) SEC analysis for aggregation level. (D) Cell surface antigen binding was detected by flow cytometric analysis in HER2-positive SKOV3 and HER2-negative NIH-3T3 cells. (E) The cytotoxicity of **TH-C8H**. (F) Bystander killing effect of **TH-C8H** in the HER2-positive SKBR3 cells and the HER2-negative MDA-MB-231 cells co-culture conditions *in vitro*. (Fa) Growth inhibitory activity of **TH-C8H** against SKBR3 and MDA-MB-231 cells; (Fb) Data of flow cytometric analysis, M1 peak for MDA-MB-231 cells, M2 peak for SKBR3 cells; (Fc) Numbers of SKBR3 and MDA-MB-231 viable cells. (G) Bystander killing effect of **TH-C8H** in the HER2-positive NCI-N87 cells and the HER2-negative MCF-7 cells co-culture conditions *in vitro*. (Ga) Growth inhibitory activity of **TH-C8H** against NCI-N87 and MCF-7 cells; (Gb) Data of flow cytometric analysis, M1 peak for MCF-7 cells, M2 peak for NCI-N87 cells; (Gc) Numbers of NCI-N87 and MCF-7 viable cells. (H, I) Cell cycle analysis assays (H) and apoptosis analysis (I) of SKOV3 cells were treated with **TH-C8H** at concentrations of 1, 3, and 10 nmol/L, respectively. For (E), (F), (G), (H), and (I), data are shown as mean ± SD (*n* = 3). Significance is calculated using unpaired *t*-test. (∗∗∗∗*P* < 0.0001).

Considering the targeted delivery and long-acting requirements of antitumor drugs, we constructed the antitumor ADC **TH-C8H** by conjugating HER2 targeted antibody Trastuzumab with **BAY** *via* a stable PEG12-*tert*-butyl-substituted **C8** linker (Fig. 5A), featuring a DAR of 4.5 (Fig. 5B) and an aggregation ratio of less than 1.0% (Fig. 5C). The ELISA data showed that the binding affinities of **TH-C8H** and Trastuzumab for HER2 antigen were comparable, with EC_50_ values of 2.8 *versus* 0.2 μg/mL, respectively (Supporting Information Fig. S4A). The flow cytometric analysis data showed that the fluorescence intensity of PE-labelled **TH-C8H** in HER2-positive SKOV3 cells was significantly higher than that in HER2-negative NIH-3T3 cells (Fig. 5D; Fig. S4B), indicating that **TH-C8H** specifically targeted the HER2 antigen of tumor cells. **TH-C8H** also exhibited differentiated cytotoxicity in various cell lines. **TH-C8H** exhibited high cytotoxicity in HER2-positive SKOV3, SKBR3, and NCI-N87 cell lines, with IC_50_ values of 0.47, 1.2, and 1.4 nmol/L, respectively (Fig. 5E). But this high cytotoxicity was completely abolished by the addition of uridine (100 μmol/L, Fig. 5E). Conversely, **TH-C8H** exhibited limited cytotoxicity in HER2-negative NIH-3T3 and MCF-7 cell lines, both with IC_50_ values above 100 nmol/L (Fig. 5E). These data confirmed the selectivity of **TH-C8H** for the HER2 antigen and its dependence on the *de novo* pyrimidine synthesis pathway. To evaluate the bystander effect of **TH-C8H**, a co-culture cell killing assay was carried out^35^. The HER2-positive SKBR3 cells and the HER2-negative MDA-MB-231 cells were mixed and cultured at an appropriate ratio overnight. The cells were treated with **TH-C8H** for 5 days, and then the number of living cells was determined. As shown in Fig. 5Fa, the number of living cells decreased in the **TH-C8H**-treated group. Concurrently, to determine the ratio of SKBR3 and MDA-MB-231 cells, HER2-expressing living cells were detected by flow cytometric analysis. The living cells clearly showed two peaks indicative of SKBR3 (M2 peak in Fig. 5Fb) and MDA-MB-231 cells (M1 peak in Fig. 5Fb). Based on the number of living cells and ratio of SKBR3 and MDA-MB-231 cells in each well, the number of SKBR3 or MDA-MB-231 cells was calculated. As a result, Trastuzumab killed only HER2-positive SKBR3 cells, while **TH-C8H** killed both SKBR3 and MDA-MB-231 cells (Fig. 5Fc). Similar results were obtained in co-culture experiments with HER2-positive NCI-N87 cells and HER2-negative MCF-7 cells (Fig. 5Ga‒Gc). The above data indicated that **TH-C8H** could kill tumor tissues by inducing a bystander killing effect.

Our study confirmed that **TH-C8H** and **BAY** share the same mechanisms of action. After treating SKOV3 cells with **TH-C8H** at concentrations of 1, 3, and 10 nmol/L for 24 h, the percentages of cells in S-phase were 38.4%, 63.5%, and 76.7% (Fig. 5H), and the apoptosis rates were 36.2%, 46.1%, and 65.4%, respectively (Fig. 5I). These results suggest that **TH-C8H** exerts high cytotoxicity through dose-dependent cell cycle arrest and the induction of apoptosis.

### 2.3 Antitumor ADC **TH-C8H** showed synergy with ferroptosis inducer *in vitro*

**Figure 6:**
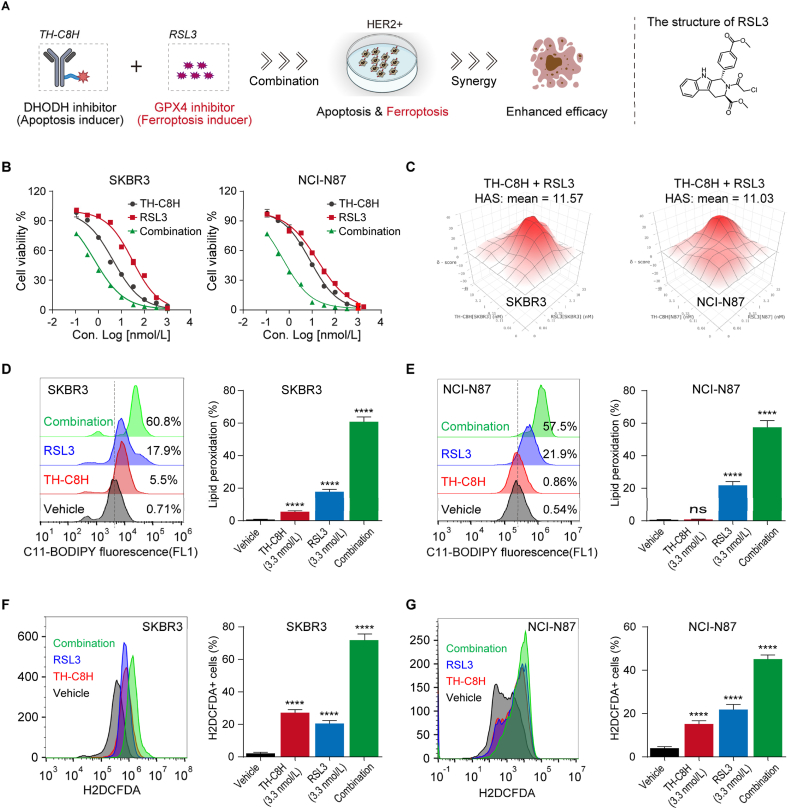
Simultaneous activation of apoptosis and ferroptosis significantly enhanced the proliferation inhibitory activity against HER2^+^ tumors. (A) The combination of DHODH inhibitor (**TH-C8H**) and GPX4 inhibitor (**RSL3**) enhances antitumor activity. (B) Effects of **TH-C8H** or **RSL3** as single agents or drug combinations in the SKBR3 (left) and NCI-N87 (right) cells. (C) Three-dimensional synergy score heatmaps of **TH-C8H** plus **RSL3** combination in SKBR3 (left) and NCI-N87 (right) cells are calculated using SynergyFinder. (D, E) Lipid peroxidation levels are determined following 72 h treatment with **TH-C8H** or **RSL3** as single agents or drug combinations in SKBR3 (D) and NCI-N87 (E) cells. Histograms show the relative change of lipid ROS. (F, G) The level of cytosolic ROS is determined after treated with **TH-C8H** or **RSL3** as single agents or drug combinations for 72 h in SKBR3 (F) and NCI-N87 (G) cells. Histograms show the relative change of cytosolic ROS. Data are shown as mean ± SD (*n* = 3). Significance is calculated using unpaired *t*-test. (∗∗∗∗*P* < 0.0001; *ns* nonsignificant).

DHODH inhibitors and ferroptosis inducers synergistically promote ferroptosis and inhibit GPX4^low^ (GPX4, for glutathione peroxidase 4) tumor growth^24^. We were interested in this finding and were inspired to further explore the synergy between a DHODH inhibitor (inducing apoptosis) and a GPX4 inhibitor (inducing ferroptosis) for antitumor applications (Fig. 6A and B).

As shown in Fig. 6B, the combination of **TH-C8H** and **RSL3**, a ferroptosis inducer, significantly triggered SKBR3 and NCI-N87 cell death. The dose-response curve of **TH-C8H** combined with **RSL3** revealed significant potency and a strong synergistic effect, as determined by the Highest Single Agent (HAS) model, with the synergy score of 11.57 (SKBR3) and 11.03 (NCI-N87), respectively (synergy score >10, Fig. 6C; Supporting Information Fig. S5A). We then investigated the underlying mechanisms, along with the synergistic effect on cell viability reduction by examining the involvement of ferroptosis, including lipid peroxidation and the promotion of cytosolic ROS levels. We found that the combination treatment significantly increased lipid peroxidation and cytosolic ROS levels compared to a moderate increase in a single treatment (Fig. 6D‒G). These findings demonstrate the highly potent synergy between DHODH inhibitor (**TH-C8H**) and ferroptosis inducer (**RSL3**) in inhibiting the growth of HER2^+^ tumor cells through ferroptosis-inducing activity. Simultaneous activation of apoptosis (induced by a DHODH inhibitor) and ferroptosis (induced by a GPX4 inhibitor) significantly enhanced the antitumor effects. This was also observed when **TH-C8H** was combined with other ferroptosis inducers, such as **Erastin**, **ML210**, and **FIN56** (Fig. S5B and S5C), suggesting that this synergy is universal.

### 2.4 Antitumor ADC **TH-C8H** exhibited significant tumor-suppressive effects *in vivo*

**Figure 7:**
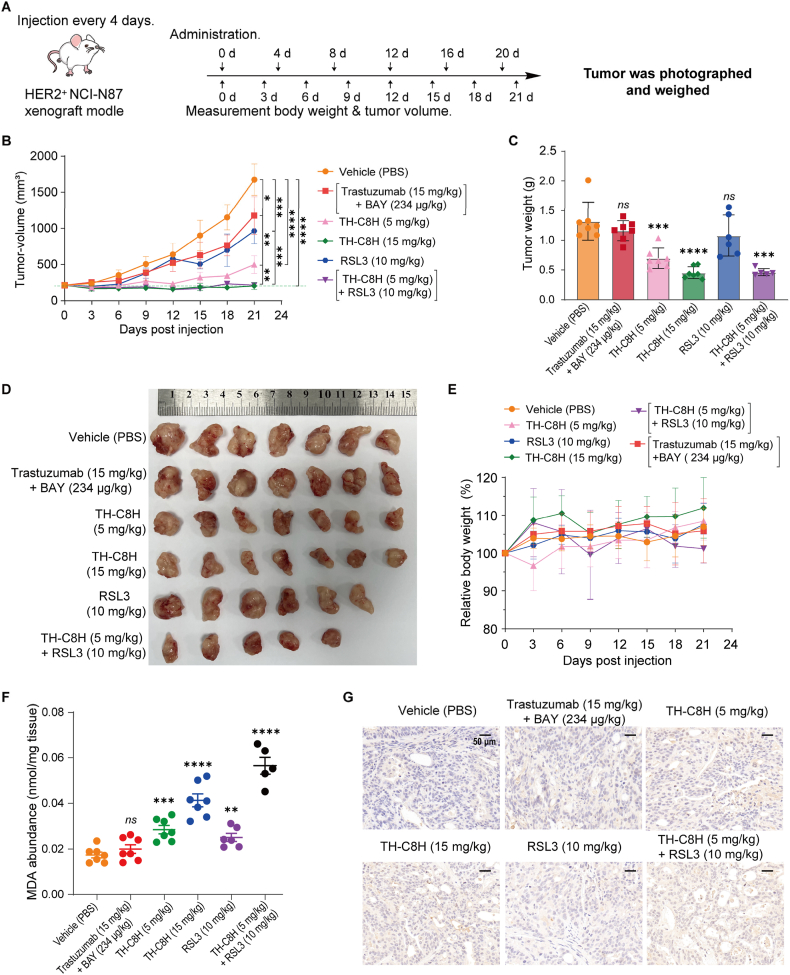
**TH-C8H** shows dose-dependent antitumor efficacy in the NCI-N87 xenograft model. (A) When tumors have reached approximately 200 mm^3^, the mice are treated as follows: vehicle (PBS), [Trastuzumab (15 mg/kg) + **BAY** (234 μg/kg)], **TH-C8H** (5 mg/kg), **TH-C8H** (15 mg/kg), **RSL3** (10 mg/kg), and [**TH-C8H** (5 mg/kg) + **RSL3** (10 mg/kg)] on Days 0, 4, 8, 12, 16, and 20. (B) Changes in tumor volume. (C, D) Tumor weights (C) and tumor tissue images (D). Tumor tissues are collected uniformly at the end of the trial. (E) Body weight changes of NOD/SCID mice during the study. (F) Malondialdehyde (MDA) level in tumor lysates. (G) 4-hydroxynonenal (4-HNE) expression in tumor. Scale bar = 50 μm. Data are shown as mean ± SD (*n* ≥ 5), and significance is calculated using two-way ANOVA (B) or unpaired *t*-test (C, F). (∗*P* < 0.05, ∗∗*P* < 0.001; ∗∗∗*P* < 0.0003; ∗∗∗∗*P* < 0.0001; *ns* nonsignificant).

Encouraged by its potent antitumor effects *in vitro*, we evaluated the antitumor efficacy of **TH-C8H** *in vivo*. In the NCI-N87 (HER2^+^) xenograft model, **TH-C8H** was administered at doses of 5 or 15 mg/kg for six consecutive doses at 4-day intervals (Fig. 7A).

Tumor volume measurements showed that **TH-C8H** exhibited a dose-dependent tumor-suppressive effect. Specifically, the tumor growth inhibition (TGI)% of **TH-C8H** was 72.1% at 5 mg/kg, which increased to 89.1% at 15 mg/kg. Conversely, the TGI% of the combined treatment group of [Trastuzumab at 15 mg/kg and **BAY** at 234 μg/kg] was only 34.6% (Fig. 7B). These data indicate that the antitumor effects of **BAY** can be significantly bolstered by the application of an ADC-targeting strategy (*P* < 0.005). To further explore the advantages of the DHODHi-based ADC, we continued to investigate the synergistic effects of **TH-C8H** in combination with **RSL3**. The combination therapy of **TH-C8H** (5 mg/kg) and **RSL3** (10 mg/kg) led to a higher TGI% of 86.5% than either monotherapy, which **TH-C8H** (5 mg/kg) with a TGI% of 72.1% and **RSL3** (10 mg/kg) with a TGI% of 43.0% (Fig. 7B).

The final tumor weight was measured to further validate the efficacy of **TH-C8H**, as well as the enhanced inhibitory efficacy of combination therapy over monotherapy (Fig. 7C and D). Additionally, monotherapy with **TH-C8H** or drug combinations showed no obvious toxicity at the applied doses, as indicated by changes in body weight during the experiments (Fig. 7E). Furthermore, the levels of malondialdehyde (MDA) in tumor lysates and lipid peroxide 4-hydroxynonenal (4-HNE) stained immunohistochemical sections of the tumors showed that **TH-C8H** and **RSL3** alone significantly increased MDA levels and 4-HNE expression, and the combination therapy group showed a more significant increase compared to the single agents (Fig. 7F and G), confirming their profound inhibitory effect on tumor growth *via* ferroptosis induction. These results confirmed that combination therapy significantly enhanced the antitumor efficacy of both monotherapies.

### 2.5 Antiviral ADC **HG-C3** exhibited broad-spectrum anti-SARS-CoV-2 activity *in vitro*

**Figure 8:**
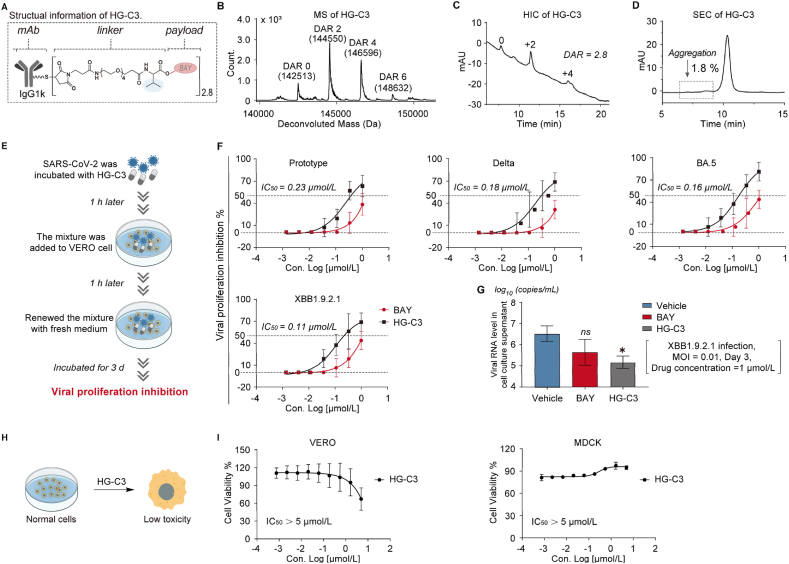
**HG-C3** exhibits anti-SARS-CoV-2 proliferative activity at the cellular level. (A) Structural information of **HG-C3**. (B–D) MS analysis (B), HIC analysis (C), and SEC analysis (D) of **HG-C3**. (E–G) Virus-challenge experiments (E) of **HG-C3** are conducted on VERO cells with SARS-CoV-2 prototype, Delta, BA.5, and XBB1.9.2.1 mutants (F), and the viral RNA loads in cell culture supernatant are tested (G). (H, I) **HG-C3** displays low toxicity (H) in normal VERO and MDCK cells (I). For (F), (G), and (I), data are shown as mean ± SD (*n* ≥ 3). Significance is calculated using unpaired *t*-test. (∗*P* < 0.05; *ns* nonsignificant).

For antiviral infection research, ADC **HG-C3** was constructed by conjugating human IgG1k antibody with **BAY** *via* a slightly less stable PEG4–iso-propyl-substituted **C3** linker (Fig. 8A; Scheme S3). **HG-C3** was expected to release **BAY** slowly into the respiratory tract to block viral invasion. Moreover, because sufficient nucleotides are necessary for viral DNA/RNA replication, any epidemic viral infection should theoretically be sensitive to DHODH activity. Thus, DHODHi-based ADCs are expected to serve as an effective broad-spectrum antiviral therapeutic agent in the future.

Quality studies showed that **HG-C3** shared a DAR of 2.8 (Fig. 8B and C) and an aggregation ratio of 1.8% (Fig. 8D). At cellular level, **HG-C3** exhibited broad-spectrum antiviral proliferative activity against various SARS-CoV-2 mutant strains including the prototype, Delta, B.A5, and XBB1.9.2.1, with IC_50_ values of 0.23, 0.18, 0.16, and 0.11 μmol/L, respectively; whereas **BAY** with IC_50_ values of above 1 μmol/L (50% inhibition was not achieved even at the highest administered concentration of 1 μmol/L; Fig. 8E and F). Subsequent analysis of cell culture supernatants infected with the XBB1.9.2.1 mutant revealed that the average viral RNA load was 1.48 × 10^5^ copies/mL in **HG-C3**-treated group, 4.44 × 10^5^ copies/mL in **BAY**-treated group, and 3.36 × 10^6^ copies/mL in control group (Fig. 8G). These data confirmed the better antiviral effect of **HG-C3** over **BAY**. In addition, cytotoxicity assessment showed that **HG-C3** exhibited limited toxicity against normal cell lines, including VERO and MDCK, both with IC_50_ values above 5 μmol/L (Fig. 8H and I). Overall, **HG-C3** exhibited broad-spectrum antiviral activity against multiple mutants while maintaining low toxicity to normal cells.

### 2.6 Antiviral ADC **HG-C3** exhibited significant antiviral efficacy *in vivo*

To clarify the distribution of **HG-C3** after nasal drops, an *in vivo* imaging test was performed. The data showed that following nasal delivery of DyLight-680 labeled **HG-C3** (10 mg/kg) to C57 mice, fluorescence was observed in the lungs within 1 h (Supporting Information Fig. S6). The fluorescence lasted for at least 48 h (Fig. S6), indicating that **HG-C3** retains in the lungs for a prolonged duration and exhibits long-lasting antiviral efficacy. This special administration method, which simulates SARS-CoV-2 invasion, contributes to increasing drug concentrations in the lungs, thereby enhancing the antiviral efficacy. In our previous *in vivo* study against influenza virus (CA07 mutant), **BAY** exhibited no antiviral efficacy even at a high dose of 3 mg/kg (Supporting Information Fig. S7). We hypothesize that this may be due to the nonspecific systemic distribution of DHODH as a host target. As a result, it is challenging to achieve the desired efficacy by directly delivering therapeutic doses of **BAY** small molecules. However, the strategy of delivering ADC *via* nasal drops may contribute to achieving long-lasting and potent antiviral efficacy *in vivo*.

**Figure 9:**
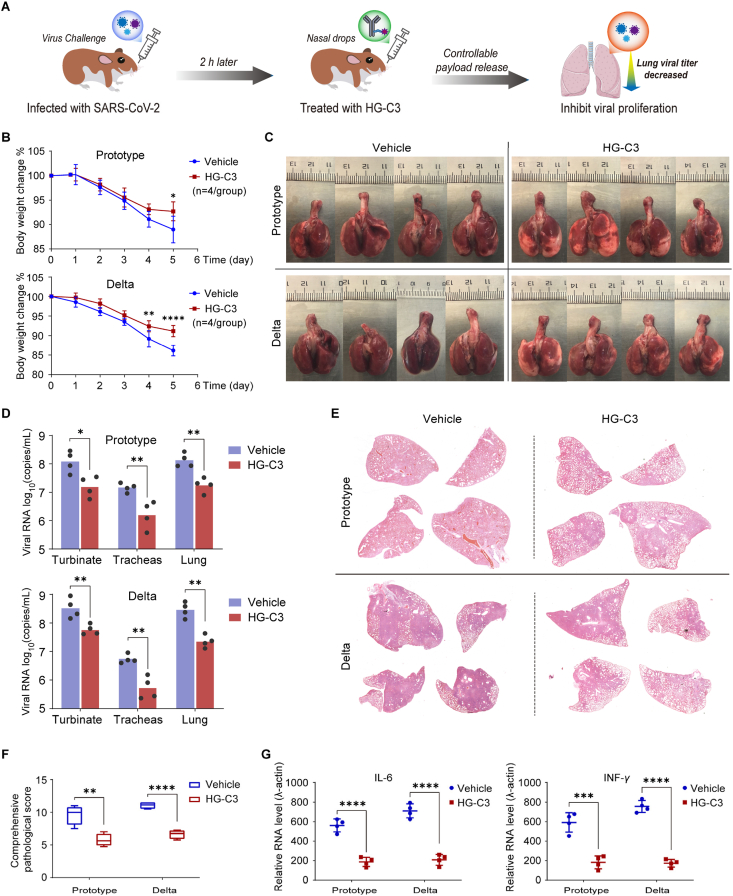
**HG-C3** shows significant efficacy against severe pneumonia caused by SARS-CoV-2 infection in a hamster model. (A) Hamsters are infected with the SARS-CoV-2 virus and nasally inoculate with **HG-C3** 2 h post-infection to observe its protective effect on the lungs. (B) Body weight changes of SARS-CoV-2-infected hamsters. (C) Gross lung images. (D) The viral RNA loads in respiratory tract tissues, including the turbinates, trachea and lungs, are measured by RT-PCR at 5 dpi. (E) Representative H&E staining of lung lobes. (F) Comprehensive pathological scores (12 lung lobes from four hamsters per group are evaluated). (G) Fold changes in the mRNA levels of proinflammatory cytokines (standardized to the housekeeping gene *γ*-actin levels). For (B), (D), (F), and (G), data are shown as mean ± SD (*n* = 4). Significance is calculated using using two-way ANOVA (B) or unpaired t-test (D, F, and G). (∗*P* < 0.05; ∗∗*P* < 0.001; ∗∗∗*P* < 0.0003; ∗∗∗∗*P* < 0.0001; *ns* nonsignificant).

To further determine the therapeutic efficacy of **HG-C3** against SARS-CoV-2 *in vivo*, we conducted a viral challenge experiment and evaluated the therapeutic activity of **HG-C3** in a hamster animal model (Fig. 9A). Considering that SARS-CoV-2 viral infection primarily affects and damages tissues and organs of the respiratory system^36^^,^^37^, we believe that respiratory medication localized to the target tissue (lungs) offers the inherent advantages of increasing the drug concentration in the target tissue (lungs) and delaying clearance. Following intranasal inoculation with the indicated doses of SARS-CoV-2 (prototype, or Delta variant), **HG-C3** was administered *via* nasal drops 2 h later.

The data showed that although hamsters were infected with different strains, there was less weight loss in the **HG-C3**-treated group. At 5 days post-infection (dpi) with the prototype or Delta variant, the body weight loss in the treated group was 7.3 ± 2.0% and 8.5 ± 1.4%, respectively, compared to 11.0 ± 2.7% and 13.8 ± 1.4% in the control group, respectively (Fig. 9B). Gross lung tissue images showed moderate to severe lung injury at 5 dpi in the control group but only mild lung injury in the **HG-C3**-treated group (Fig. 9C). Viral replication in respiratory tissues (turbinates, trachea, and lungs) was analyzed by RT-PCR amplification of SARS-CoV-2 ORF1ab to assess the viral RNA loads in tissues collected at 5 dpi. Viral RNA levels in all three tissues showed a similar trend, with an approximately 10-fold reduction in the **HG-C3**-treated group compared with that in the control group (Fig. 9D). Hematoxylin and eosin (H&E) staining of fixed lung lobes and comprehensive pathological scoring confirmed that **HG-C3** significantly reduced lung damage caused by viral infection in hamsters (Fig. 9E and F). Given the close link between viral clearance efficiency, the severity of pneumonia caused by SARS-CoV-2, and host innate immune responses, we measured the mRNA levels of typical proinflammatory cytokines and type I interferon (IFN)-related genes in homogenized lung tissues *via* RT-PCR. The **HG-C3**-treated group showed 3‒5 fold lower expression of typical proinflammatory cytokines, including interleukin 6 (IL-6) and interferon-*γ* (IFN-*γ*) at 5 dpi, indicating reduced lung inflammation (Fig. 9G). These results suggest that **HG-C3** attenuates the inflammatory responses in the lungs.

## 3 Discussion

In this study, we discovered a novel payload and developed a novel class of DHODHi-based ADCs. Owing to the key role of DHODH in pyrimidine synthesis, novel ADCs hold great promise for the treatment of diseases characterized by rapid proliferation, such as tumors and viral infections. Targeted delivery of ADCs significantly enhances the antitumor effects and minimizes toxicity to normal tissues. Another important discovery was that DHODHi-based ADC exhibited a synergistic effect with ferroptosis inducers in the nanomolar range, which was a breakthrough over previous studies on DHODH inhibitors by Mishima et al.^24^^,^^27^ (with IC_50_ in the micromolar range). However, the underlined mechanism was needed to be further clarified. This study provides diverse options for future antitumor medications and lays the foundation for drug combinations. Meanwhile, we discovered the first antiviral ADC, which provides new insights into the development of broad-spectrum antiviral therapies.

Notably, the presence of the hydroxyl group in **BAY** complicates the design of plasma-stable linkers. The marketed ADC **IMMU-132** uses a carbonate bond for linkage and also lacks stability in plasma^37^^,^^38^. For the first time, we have developed novel linkers with controllable stability by modulating the site-blocking effect of the *α*-site carbonyl carbon atom to meet the differentiated requirements of acute viral infections and chronic tumors. In addition, the innovative delivery method can deliver ADCs directly to the respiratory system and achieve a prolonged protective effect covering turbinates, trachea, and lungs through sustained drug release. Nasal drops of **HG-C3** significantly reduced viral load in three tissues and suppressed the inflammatory response in the lungs.

Despite these satisfactory findings, challenges and limitations remain. The activity of DHODHi still remains the potential to be further improved by structural modification. The in-depth exploration of other DHODH inhibitors featuring novel structures such as acrylamide derivatives^39^ and naphtho[2,3-*d*][1,2,3]triazole-4,9-dione derivatives^40^ brings additional opportunities for the development of DHODHi-based ADCs. The efficacy of the ADC may also be further improved by linker optimization, including applying new conjugating approaches and raising the DAR value. In the future, the efficacy of antitumor ADCs should be evaluated in additional models, and the prescription and dosage of drug combinations should be further optimized. The *in vivo* evaluation of the ADC bystander effect also remains to be updated and refined. The broad-spectrum antiviral efficacy of antiviral ADCs remains to be demonstrated on a wider range of mutant strains. These are all necessary steps for the clinical success of novel ADCs. Given the multi-effect nature of DHODH inhibition, DHODHi-based ADCs are not limited to antitumor and antiviral but also hold the promise of being developed into a potent DHODH-targeted therapy for anti-infective^41^ and for the treatment of immune disorders, such as rheumatoid arthritis^39^. Fundamentally, our study offers an innovative concept for developing broad-spectrum antitumor and antiviral ADCs, which is crucial for expanding the therapeutic applications of ADCs.

## 4 Conclusions

Our studies have shown that DHODHis is a novel payload class for generating broad-spectrum ADCs against tumors and viral infections. The innovation of stability-controllable linkers has promoted this discovery. DHODHi-based ADCs targeting HER2 antigen demonstrated potent and selective anti-proliferative activity in a panel of HER2-positive tumor cells *in vitro* and high antitumor efficacy *in vivo*. Local administration of DHODHi-based ADC achieved broad-spectrum antiviral efficacy in animal models. Thus, ADCs with DHODHi as payload offer a promising novel approach for broad-spectrum antitumor and antiviral treatments.

## Author contributions

Zhirui Liu: Writing – original draft, Investigation, Methodology. Lunzhi Yuan: Methodology, Writing – original draft, Investigation. Pengyun Li: Writing – original draft, Investigation, Methodology. Fei Xie: Methodology, Investigation. Ming Zhou: Methodology, Investigation. Lianqi Liu: Methodology, Investigation. Ting Wei: Methodology, Investigation. Yi Guan: Supervision, Resources. Ningshao Xia: Resources, Investigation. Zhibing Zheng: Supervision, Resources. Tong Cheng: Supervision, Resources. Dian Xiao: Resources, Writing – review & editing, Funding acquisition. Xinbo Zhou: Writing – review & editing, Funding acquisition, Supervision. Song Li: Resources, Supervision, Project administration.

## Conflicts of interest

The authors have no conflicts of interest to declare.

## Appendix A Supplementary data

The following is the Supporting Information to this article:
