# Supplementary material for: Antibody‒drug conjugates with DHODH inhibitor as novel payload class for cancer and SARS-CoV-2 infection therapies
Source: Acta Pharm Sin B. 2025 Nov 12;16(2):1046–58. doi: 10.1016/j.apsb.2025.11.008 (PMC12891887; doi:10.1016/j.apsb.2025.11.008)
Supplement: Multimedia component 1 [file mmc1.pdf]

## Supporting Information for

## ORIGINAL ARTICLE

# Antibody–drug conjugates with DHODH inhibitor as novel payload class for cancer and SARS-CoV-2 infection therapies

**Zhirui Liu[a,b,†], Lunzhi Yuan[c,†], Pengyun Li[b,†], Fei Xie[b], Ming Zhou[c], Lianqi Liu[b], Ting Wei[b], Yi Guan[d,e], Ningshao Xia[c], Zhibing Zheng[b,*], Tong Cheng[c,*], Dian Xiao[b,*], Xinbo Zhou[b,*], Song Li[a,b,*]**

[a]*School of Pharmaceutical Engineering, Shenyang Pharmaceutical University, Shenyang 110016, China*

[b]*National Engineering Research Center for the Emergency Drug, Beijing Institute of Pharmacology and Toxicology, Beijing 100850, China*

[c]*State Key Laboratory of Molecular Vaccinology and Molecular Diagnostics, National Institute of Diagnostics and Vaccine Development in Infectious Diseases School of Life Sciences, School of Public Health, Xiamen University, Xiamen 361000, China*

[d]*State Key Laboratory of Emerging Infectious Diseases, School of Public Health, Li Ka Shing Faculty of Medicine, The University of Hong Kong, Hong Kong SAR 999077, China*

[e]*Guangdong-Hong Kong Joint Laboratory of Emerging Infectious Diseases, Joint Laboratory for International Collaboration in Virology and Emerging Infectious Diseases, Joint Institute of Virology (STU/HKU), Shantou University, Shantou 515000, China*

Received 3 June 2024; received in revised form 10 August 2025; accepted 18 October 2025

*Corresponding authors.

E-mail addresses: lis.lisong@gmail.com (Song Li), zzbcaptain@aliyun.com (Zhibing Zheng), tcheng@xmu.edu.cn (Tong Cheng), xiaodian@bmi.ac.cn (Dian Xiao), zhouxinbo@bmi.ac.cn (Xinbo Zhou).

[†]These authors made equal contributions to this work.

## 5. Experimental

### *5.1. Reagents and materials*

**BAY 22402234** (**BAY**) API were purchased from MedChemExpress (MCE). Trastuzumab (Herceptin®) and human IgG1k were obtained from Sino Biological Inc. Other reagents and solvents, unless specified otherwise, were commercially available and used without further purification. All reactions requiring anhydrous conditions were performed under an argon atmosphere. The reaction process was monitored *via* thin-layer chromatography (TLC). High-performance liquid chromatography (HPLC) analyses were performed on an Agilent 1260 series liquid chromatography (LC) system (CA, USA). NMR spectra were recorded on JNM-ECA-400 (JEOL Co., Ltd.). Chemical shifts are reported in ppm, and tetramethylsilane (TMS) is used as the internal standard. Coupling constants (*J*) are given in Hertz, while spin multiplicities are denoted as the following abbreviations: s (singlet), d (doublet), dd (doublet doublet), t (triplet), q (quadruplet), and m (multiplet). Mass spectrometry (MS) was performed on a time-of-flight mass spectrometer (TOF-MS) using electrospray ionization (Agilent, 6200 series TOF, Canada).

All cells were purchased from the American Type Culture Collection. The Human ovarian carcinoma cell line SKOV3 and breast carcinoma cell line SKBR3 were cultured in high-glucose DMEM supplemented with 10% fetal bovine serum (FBS) and 0.1% penicillin–streptomycin. The human gastric cancer cell line NCI-N87 was cultured in RPMI-1640 with 10% FBS and 0.1% penicillin-streptomycin.

### *5.2. Synthesis of ADCs (**TH-A1–A3**, **TH-B1–B2**, **TH-C1–C8**, **TH-C8H**, and **HG-C3**)*

#### *5.2.1. Synthesis of ADCs **TH-A1–A3***

The synthesis route of **TH-A1**–**A3** and **TH-B1**–**B2** was shown in Scheme S1. Compound **1** was obtained by the reaction of Fmoc-VA-PAB with Boc-ethylenediamine derivatives. Compound **2** was obtained by removing protective groups from **1**. Compound **2** reacted with **BAY** to yield **3**. The removal of the Fmoc protecting group from **3** yielded **4**, which contained an exposed amino group. The amino group of **4** reacted with the maleimide NHS active ester derivative to obtain the target small molecule **A1**. **A1** was conjugated with the thiol group of Trastuzumab monoclonal antibody to prepare **TH-A1** (Scheme S1a). Compound **5** was obtained by reaction of Fmoc-Val-Ala with 2-(aminomethyl)-1-*Boc*-Piperidine. Compound **6** is obtained by removing the Fmoc protective group from **5**. Compound **6** reacted with **7** to obtain **8**. The removal of the Fmoc protecting group from **8** yielded **9**, which contained an exposed amino group. The amino group of **9** undergoes an amide-forming reaction with maleimide derivatives containing different PEG polymerization degrees to yield the target small molecules **A2** (PEG4) and **A3** (PEG8).

**A2**–**A3** were conjugated with Trastuzumab to prepare **TH-A2**–**A3** respectively (Scheme S1b).

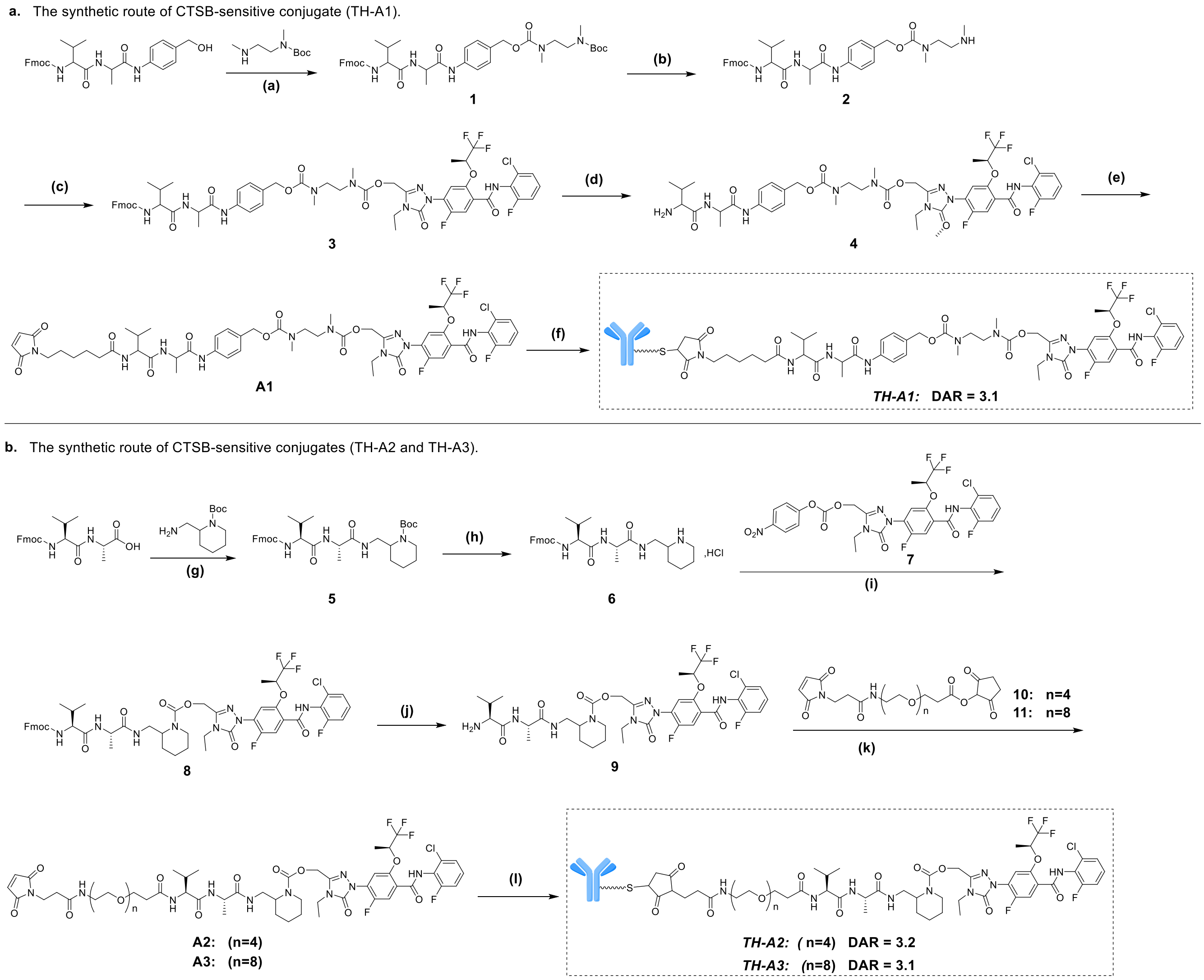


**Scheme S1** Synthesis of conjugates with CTSB-sensitive linkers (**TH-A1**–**A3**). Reagents and conditions: (a) Bis(4-nitrophenyl) carbonate, DIPEA, DMF; (b) TFA, DCM; (c) Bis(4-nitrophenyl) carbonate, DIPEA, DMF; (d) 10% Piperidine, DMF; (e) DIPEA/DMF; (f) Trastuzumab, TCEP, NAC, Buffer; (g) EDCI, DIPEA, HoBt, DMF, 12h; (h) HCl, EA, 1h; (i) Bis(4-nitrophenyl) carbonate, DIPEA, DMF, 12h; (j) 10% Piperidine, DMF; (k) DIPEA, DMF; (l) Trastuzumab, TCEP, NAC, Buffer.

*5.2.1.1. Synthesis of (1-(4-((2-chloro-6-fluorophenyl)carbamoyl)-2-fluoro-5-(((S)-1,1,1-trifluoropropan-2-yl)oxy)phenyl)-4-ethyl-5-oxo-4,5-dihydro-1H-1,2,4-triazol-3-yl)methyl (4-(25-(2,5-dioxo-2,5-dihydro-1H-pyrrol-1-yl)-5-isopropyl-2-methyl-4,7,23-trioxo-10,13,16,19-tetraoxa-3,6,22-triazapentacosanamido)*

*benzyl) ethane-1,2-diylbis(methylcarbamate)* ***(A1)***. $^1$H-NMR (400 MHz, $CDCl_3$): $\delta$ 9.03 (s, 1H), 8.67 (s, 1H), 8.15 (d, J=7.0, 1H), 7.67 (m, 1H) 7.50 (m, 1H), 7.29 (m, 2H), 7.24 (m, 1H), 7.13 (t, J=5.8, 1H), 6.93

(s, 1H), 6.69 (s, 2H), 6.51 (s, 1H), 5.08 (m, 3H), 4.62 (m, 1H), 4.19 (s, 1H), 3.82 (m, 5H), 3.70 (m, 1H), 3.61 (m, 12H), 3.51 (m, 3H), 3.40 (m, 4H), 2.91 (m, 6H), 2.73 (s, 1H), 2.52 (m, 2H), 1.65 (m, 3H), 1.43 (m, 4H), 1.37 (m, 2H), 1.29 (s, 2H), 1.26 (s, 6H), 1.00 (d, J=4.6, 3H), 0.98 (d, J=4.6, 3H). HR-MS (ESI+) *m/z*: 1374.4843 $[M+Na]^+$; 674.3390 $[M+2H]^{2+}$.

*5.2.1.2. Synthesis of tert-butyl 2-((5S,8S)-1-(9H-fluoren-9-yl)-5-isopropyl-8-methyl-3,6,9-trioxo-2-oxa-4,7,10-triazaundecan-11-yl)piperidine-1-carboxylate* ***(5)***. To a solution of Fmoc-Val-Ala-COOH (2.0 g, 4.9 mmol) in anhydrous *N,N*-dimethylformamide (DMF), 1-(3-dimethylaminopropyl)-3-ethylcarbodiimide hydrochloride (EDCI) (1.0 g, 5.3 mmol) and 1-hydroxybenzotriazole (HoBt) (0.72 g, 5.3 mmol) was added. After stirring for 1 h, 2-(aminomethyl)-1-*Boc*-piperidine (0.87 g, 4.1 mmol) and *N,N*-diisopropylethylamine (DIPEA) (0.79 g, 6.1 mmol) were added at the same time. The mixture continued to be stirred overnight or until the starting material disappeared completely (monitored by TLC). After evaporation of the solvent under vacuum, the crude product (pale yellow oil) was purified by chromatography on silica gel (dichloromethane/methanol = 5:1, *v/v*) to give **(5)** as an off-white solid (1.26 g, 81.3% yield). $^1$H-NMR (400 MHz, $CDCl_3$): $\delta$ 7.76 (d, J=5.0, 2H), 7.59 (m, 2H),7.40 (dt, 2H), 7.31 (m, 2H), 6.54 (m, 2H), 5.46 (m, 1H), 4.40 (m, 4H), 4.22 (m, 1H), 3.99 (m, 2H), 3.71 (m, 1H), 3.20 (m, 1H), 2.96 (s, 1H), 2.88 (s, 1H), 2.83 (m, 1H), 2.13 (m, 1H), 1.60 (m, 4H), 1.45 (m, 9H), 1.36 (m, 3H), 0.96 (m, 6H). HR-MS (ESI+) *m/z*: 607.3490 $[M+H]^+$.

*5.2.1.3. Synthesis of (9H-fluoren-9-yl)methyl ((2S)-3-methyl-1-oxo-1-(((2S)-1-oxo-1-((piperidin-2-ylmethyl)amino)propan-2-yl)amino)butan-2-yl)carbamate* ***(6)***. To a solution of **5** (1.1 g, 1.8 mmol) in hydrogen chloride ethyl acetate solution (4 mol/L), continued to be stirred for 30 min or until the starting material disappeared completely. After evaporation of the solvent under vacuum, the crude product was purified by chromatography on silica gel (dichloromethane/methanol = 10:1, *v/v*) to give **6** as an off-white solid (0.81 g, 88.7% yield). $^1$H-NMR (400 MHz, $CD_3OD$): $\delta$ 7.81 (m, 2H), 7.67 (m, 2H), 7.39 (m, 2H), 7.31 (m, 2H), 4.45 (m, 1H), 4.35 (m, 1H), 4.25 (m, 1H), 4.18 (m, 1H), 3.88 (m, 1H), 3.54 (m, 1H), 3.35 (m, 1H), 3.23 (m, 2H), 2.91 (m, 1H), 2.05 (m, 1H), 1.85 (m, 2H), 1.68 (m, 2H), 1.48 (m, 2H), 1.38 (m, 4H), 1.03 (q, 1H), 0.97 (m, 6H). HR-MS (ESI+) *m/z*: 507.2963 $[M+H]^+$.

*5.2.1.4. Synthesis of (S)-(1-(4-((2-chloro-6-fluorophenyl)carbamoyl)-2-fluoro-5-((1,1,1-trifluoropropan-2-yl)oxy)phenyl)-4-ethyl-5-oxo-4,5-dihydro-1H-1,2,4-triazol-3-yl)methyl (4-nitrophenyl) carbonate* ***(7)***. To a solution of **BAY** (100 mg, 0.19 mmol) in anhydrous DMF was added Bis (4-nitrophenyl) Carbonate (NPC). Around 10 min later, added DIPEA (37 mg, 0.29 mmol) at room temperature. The mixture continued to be stirred overnight or until the starting material disappeared completely. After evaporation

of the solvent under vacuum, the crude product was purified by chromatography on silica gel (petroleum ether/ethyl acetate = 2:1, *v/v*) to give **7** as pale yellow solid (51 mg, 38.7% yield) that was directly used in the next step without further purification. HR-MS (ESI+) *m/z*: 686.1071 $[M+H]^+$.

*5.2.1.5. Synthesis of (1-(4-((2-chloro-6-fluorophenyl)carbamoyl)-2-fluoro-5-(((S)-1,1,1-trifluoropropan-2-yl)oxy)phenyl)-4-ethyl-5-oxo-4,5-dihydro-1H-1,2,4-triazol-3-yl)methyl 2-((5S,8S)-1-(9H-fluoren-9-yl)-5-isopropyl-8-methyl-3,6,9-trioxo-2-oxa-4,7,10-triazaundecan-11-yl)piperidine-1-carboxylate* ***(8)***. To a solution of **6** (52 mg, 0.095 mmol) in anhydrous DMF, **7** (50 mg, 0.073 mmol) and 1-Hydroxybenzotriazole (HoBt) (9.8 mg, 0.073 mmol) were added successively. When raw solid materials dissolved completely, DIPEA (19 mg, 0.15 mmol) was added at room temperature. The mixture continued to be stirred overnight or until the starting material disappeared completely. After evaporation of the solvent under vacuum, the crude product (pale yellow oil) was purified by chromatography on silica gel (dichloromethane/methanol = 50:1, *v/v*) to give **8** as an off-white solid (37 mg, 48.2% yield). $^1$H-NMR (400 MHz, $CDCl_3$): $\delta$ 9.02 (s, 1H), 8.17 (d, 1H), 8.02 (s, 1H), 7.75 (m, 2H), 7.56 (m, 2H),7.39 (m, 2H), 7.29 (m, 2H), 7.23 (m, 1H), 7.13 (m, 1H), 6.59 (m, 2H), 5.01 (m, 2H), 4.41 (m, 4H), 4.21 (m, 1H), 3.88 (m, 3H), 3.05 (m, 2H), 2.96 (m, 3H), 2.89 (m, 3H), 2.08 (s, 1H), 1.64 (m, 8H), 1.36 (m, 6H), 0.90 (m, 6H). HR-MS (ESI+) *m/z*: 1053.3684 $[M+H]^+$; 1075.3500 $[M+Na]^+$.

*5.2.1.6. Synthesis of (1-(4-((2-chloro-6-fluorophenyl)carbamoyl)-2-fluoro-5-(((S)-1,1,1-trifluoropropan-2-yl)oxy)phenyl)-4-ethyl-5-oxo-4,5-dihydro-1H-1,2,4-triazol-3-yl)methyl 2-(((S)-2-((S)-2-amino-3-methylbutanamido)propanamido)methyl)piperidine-1-carboxylate* ***(9)***. To a solution of **8** (127 mg, 0.12 mmol) in 10 mL anhydrous DMF was added 1 mL piperazine. The mixture continued to be stirred for 30 min. After evaporation of the solvent under vacuum, the crude product (pale yellow solid) was purified by chromatography on silica gel (dichloromethane/methanol = 10:1, *v/v*) to give **9** as an off-white solid (91 mg, 90.9% yield). $^1$H-NMR (400 MHz, $CDCl_3$): $\delta$ 9.03 (s, 1H), 8.17 (d, 1H), 7.72 (m, 1H), 7.54 (m, 1H),7.29 (m, 1H), 7.23 (m, 1H), 7.13 (t, 1H), 6.66 (m, 1H), 5.30 (s, 1H), 5.17 (m, 2H), 4.98 (m, 1H), 4.42 (m, 3H), 3.89 (m, 4H), 3.14 (m, 3H), 2.24 (m, 1H), 1.91 (s, 1H), 1.66 (m, 4H), 1.36 (m, 8H), 0.97 (m, 3H) 0.84 (m, 3H). HR-MS (ESI+) *m/z*: 831.3015 $[M+H]^+$.

*5.2.1.7. Synthesis of (1-(4-((2-chloro-6-fluorophenyl)carbamoyl)-2-fluoro-5-(((S)-1,1,1-trifluoropropan-2-yl)oxy)phenyl)-4-ethyl-5-oxo-4,5-dihydro-1H-1,2,4-triazol-3-yl)methyl 2-((4S,7S)-27-(2,5-dioxo-2,5-dihydro-1H-pyrrol-1-yl)-7-isopropyl-4-methyl-3,6,9,25-tetraoxo-12,15,18,21-tetraoxa-2,5,8,24-tetraazaheptacosyl)piperidine-1-carboxylate* ***(A2)***. To a solution of **9** (30 mg, 0.036 mmol) and **10** (28 mg, 0.054 mmol) in anhydrous DMF, DIPEA (9.5 mg, 0.072 mmol) was added. The mixture continued

to be stirred overnight or until the starting material disappeared completely. After evaporation of the solvent under vacuum, the crude product was purified by chromatography on silica gel (dichloromethane/methanol = 10:1, *v/v*) to give **A2** as an off-white solid (48 mg, 108.2% yield). $^1$H-NMR (400 MHz, $CDCl_3$): $\delta$ 9.04 (s, 1H), 8.16 (d, *J*=7.3, 1H), 7.54 (s, 1H), 7.28 (m, 1H), 7.24 (m, 1H), 7.13 (m, 1H), 6.70 (m, 3H), 6.50 (s, 1H), 5.15 (m, 4H), 4.40 (m, 2H), 4.16 (m, 2H), 3.84 (m, 4H), 3.74 (s, 1H), 3.64 (m, 12H), 3.54 (s, 2H), 3.41 (s, 2H), 3.09 (m, 3H), 2.74 (s, 1H), 2.53 (m, 4H), 2.22 (s, 1H), 1.66 (m, 3H), 1.33 (m, 12H), 0.95 (m, 6H). HR-MS (ESI+) *m/z*: 1251.4512 $[M+Na]^+$; 615.2382 $[M+2H]^{2+}$.

*5.2.1.8. Synthesis of (1-(4-((2-chloro-6-fluorophenyl)carbamoyl)-2-fluoro-5-(((S)-1,1,1-trifluoropropan-2-yl)oxy)phenyl)-4-ethyl-5-oxo-4,5-dihydro-1H-1,2,4-triazol-3-yl)methyl 2-((4S,7S)-39-(2,5-dioxo-2,5-dihydro-1H-pyrrol-1-yl)-7-isopropyl-4-methyl-3,6,9,37-tetraoxo-12,15,18,21,24,27,30,33-octaoxa-2,5,8,36-tetraazanonatriacontyl)piperidine-1-carboxylate* ***(A3)***. To a solution of **9** (30 mg, 0.036 mmol) and **11** (35 mg, 0.051 mmol) in anhydrous DMF, DIPEA (9.3 mg, 0.072 mmol) was added. The mixture continued to be stirred overnight or until the starting material disappeared completely. After evaporation of the solvent under vacuum, the crude product was purified by chromatography on silica gel (dichloromethane/methanol = 15:1, *v/v*) to give **A3** as a semi-transparent solid (46 mg, 90.6% yield). $^1$H-NMR (400 MHz, $CDCl_3$): $\delta$ 9.04 (s, 1H), 8.16 (d, 1H), 7.54 (m, 1H), 7.29 (m, 1H), 7.24 (m, 1H), 7.13 (m, 1H), 6.70 (m, 3H), 6.35 (s, 1H), 5.30 (s, 1H), 5.08 (m, 4H), 4.39 (m, 2H), 4.15 (m, 1H), 3.85 (m, 5H), 3.64 (m, 28H), 3.54 (t, J=3.4, 2H), 3.41 (q, J=3.5, 2H), 3.03 (m, 2H), 2.73 (s, 2H), 2.54 (m, 3H), 2.23 (m, 1H), 1.65 (m, 8H), 1.34 (m, 6H), 0.95 (m, 6H). HR-MS (ESI+) *m/z*: 703.2899 $[M+2H]^{2+}$.

*5.2.1.9. Synthesis of ADCs* ***TH-A1–A3****. Standard Procedure A* for the preparation of ADCs. The Monoclonal antibody was exchanged into a buffer solution (20 mmol/L L-Histidine, pH = 7.2–7.4), and TCEP (2.1 molar equivalent) was subsequently added to reduce the interchain disulfide bond. Approximately 2 h later, a small molecule compound in DMA (8 molar equivalent) was added to the solution. The final volume percentage of the organic solvent was maintained at 8%, and the mixture was stirred at 25 °C for 4 h with gentle mixing. The final concentration of antibody used in the reactions was 5 mg/mL. The reaction was halted by the addition of excess *N*-acetylcysteine (NAC) for 30 min. The conjugates were then transferred into phosphate buffered saline (PBS) using Sephadex-G25S to remove the free NAC and small molecule compound. Finally, the conjugates were analyzed or stored at −80 °C for further use.

*Standard Procedure B:* To further elevate the DAR of conjugates, Trastuzumab in reaction buffer solution was treated with 8 mol equivalent TCEP for 4 h to reduce all the interchain disulfide bridges, then 16 mol equivalent of compound **C8** in DMA was added to the solution. The conjugation reaction

was allowed to proceed at room temperature for 12 h. Other operations were the same as Standard Procedure A.

Compounds **A1**–**A3** were conjugated to Trastuzumab by *Standard Procedure A* respectively to prepare **TH-A1**–**A3**.

*5.2.2. Synthesis of ADCs **TH-B1–B2***

The target small molecule **B1** was prepared by combining **BAY** with maleimide polyethylene glycol derivatives and Dichlorodiisopropylsilane by a one-pot method. **B1** was prepared by combining **BAY** with maleimide polyethylene glycol derivative and phosgene by a one-pot method. **B2** was prepared by combining **10** with maleimide carboxylic acid derivatives. **B1**–**B2** were conjugated with Trastuzumab to prepare **TH-B1**–**B2**, respectively (Scheme S2).

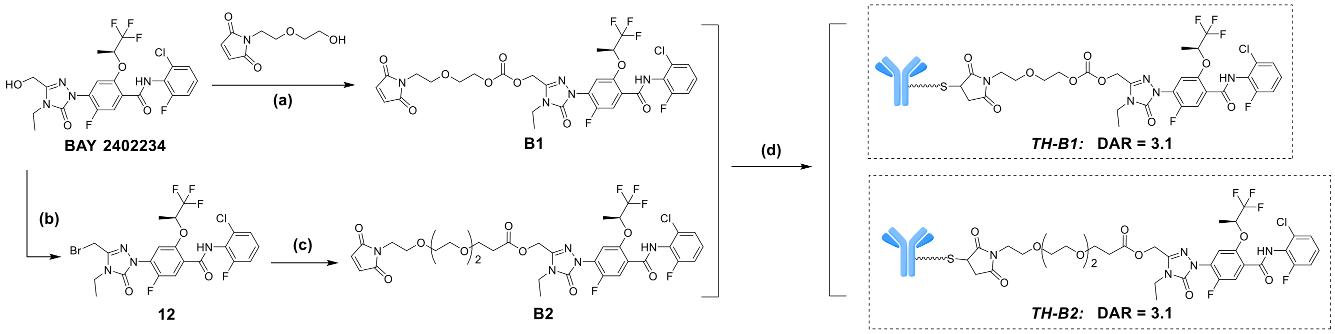


**Scheme S2.** Synthesis of conjugates with pH-sensitive linkers (**TH-B1**–**B2**). Reagents and conditions: (a) BTC, pyridine, DCM, 0 °C; (b) PBr3, DCM, overnight; (c) Potassium carbonate, DMF, 10–30 min; (d) Trastuzumab, TCEP, NAC, Buffer.

*5.2.2.1. Synthesis of (S)-(1-(4-((2-chloro-6-fluorophenyl)carbamoyl)-2-fluoro-5-((1,1,1-trifluoropropan-2-yl)oxy)phenyl)-4-ethyl-5-oxo-4,5-dihydro-1H-1,2,4-triazol-3-yl)methyl (2-(2-(2,5-dioxo-2,5-dihydro-1H-pyrrol-1-yl)ethoxy)ethyl) carbonate (**B1**)*. $^{1}$H-NMR (400 MHz, $CDCl_3$): $\delta$ 9.01 (s, 1H), 8.18 (d, $J$=7.5, 1H), 7.47 (d, $J$=3.6, 1H), 7.29 (m, 1H), 7.24 (m, 1H), 7.13 (m, 1H), 6.71 (s, 2H), 4.95 (m, 1H), 4.32 (m, 2H), 3.88 (q, $J$=4.8, 2H), 3.70 (m, 8H), 1.66 (d, $J$=4.3, 3H), 1.42 (t, $J$=4.8, 3H). HR-MS (ESI+) *m/z*: 732.1489 $[M+H]^+$; 754.1315 $[M+Na]^+$.

*5.2.2.2. Synthesis of (S)-(1-(4-((2-chloro-6-fluorophenyl)carbamoyl)-2-fluoro-5-((1,1,1-trifluoropropan-2-yl)oxy)phenyl)-4-ethyl-5-oxo-4,5-dihydro-1H-1,2,4-triazol-3-yl)methyl 3-(2-(2-(2-(2,5-dioxo-2,5-dihydro-1H-pyrrol-1-yl)ethoxy)ethoxy)ethoxy) propanoate (**12**)*. To a solution of **BAY** (50 mg, 0.096 mmol) in anhydrous Dichloromethane (DCM) was added Triphenylphosphine (34 mg, 0.125 mmol) at room temperature. The mixture continued to be stirred overnight or until the starting material disappeared completely. After evaporation of the solvent under vacuum, the crude product was purified by

chromatography on silica gel (dichloromethane/ethyl acetate = 10:1, *v/v*) to give **12** as white solid (63 mg, 100% yield). $^1$H-NMR (400 MHz, $CDCl_3$): $\delta$ 9.01 (s, 1H), 8.19 (d, *J*=7.6, 1H), 7.49 (d, *J*=3.7, 1H), 7.29 (m, 1H), 7.24 (m, 1H), 7.13 (m, 1H), 4.95 (m, 1H), 4.36 (s, 2H), 3.92 (t, *J*=4.8, 2H), 1.66 (d, *J*=4.3, 3H), 1.48 (t, *J*=4.8, 3H). HR-MS (ESI+) *m/z*: 585.0144 $[M(^{81}Br) +H]^+$; 606.9958 $[M(^{81}Br) +Na]^+$.

*5.2.2.3. Synthesis of (S)-(1-(4-((2-chloro-6-fluorophenyl)carbamoyl)-2-fluoro-5-((1,1,1-trifluoropropan-2-yl)oxy)phenyl)-4-ethyl-5-oxo-4,5-dihydro-1H-1,2,4-triazol-3-yl)methyl 3-(2-(2-(2-(2,5-dioxo-2,5-dihydro-1H-pyrrol-1-yl)ethoxy)ethoxy)ethoxy) propanoate* ***(B2)***. To a solution of Mal–PEG3–propionic acid (52 mg, 0.11 mmol) in anhydrous Dichloromethane (DCM) was added **12** (30 mg, 0.051 mmol) and anhydrous potassium carbonate (34 mg, 0.25 mmol) at room temperature. The mixture continued to be stirred for 10 min or until the starting material disappeared completely. After filtration of insoluble solid materials, the crude product was obtained by evaporation of the solution under vacuum and then purified by flash chromatography on silica gel (dichloromethane/methanol = 10:1, *v/v*) to give **B2** as a semi-transparent solid (25 mg, 50.3% yield). $^1$H-NMR (400 MHz, $CDCl_3$): $\delta$ 9.01 (s, 1H), 8.18 (d, *J*=7.6, 1H), 7.48 (d, *J*=3.7, 1H), 7.29 (m, 1H), 7.24 (m, 1H), 7.13 (m, 1H), 6.70 (s, 2H), 5.13 (s, 2H), 4.95 (m, 1H), 3.84 (q, *J*=4.8, 2H), 3.80 (t, J=4.1, 2H), 3.72 (m, 2H), 3.61 (m, 9H), 2.70 (t, *J*=4.1, 2H), 1.66 (d, *J*=4.3, 3H), 1.39 (t, *J*=4.8, 3H). HR-MS (ESI+) *m/z*: 804.2066 $[M+H]^+$; 826.1891 $[M+Na]^+$.

*5.2.2.4. Synthesis of ADCs* ***TH-B1–B2*** Compounds **B1**–**B2** were conjugated to Trastuzumab by *Standard Procedure A* respectively to prepare **TH-B1**–**B2**.

*5.2.3. Synthesis of ADCs* ***TH-C1–C8****,* ***TH-C8H****, and* ***HG-C3***

The synthesis route was shown in Scheme S3. First, the hydroxyl group in structure **BAY** was substituted for bromine, resulting in the key intermediate **12**. Then, maleimide carboxylic acid derivatives were reacted with NHS to prepare reactive ester intermediates **13**–**15**. **13**–**15** were reacted with different kinds of amino acids, which with specific R-group, to yield compounds **22**–**29**. Then **22**–**29** were reacted with the key intermediate **12** to yield the target small molecules **C1**–**C8**, respectively. **C1**–**C8** were conjugated with the sulfhydryl group of Trastuzumab monoclonal antibody to obtain the target conjugates **TH-C1**–**C8**, respectively.

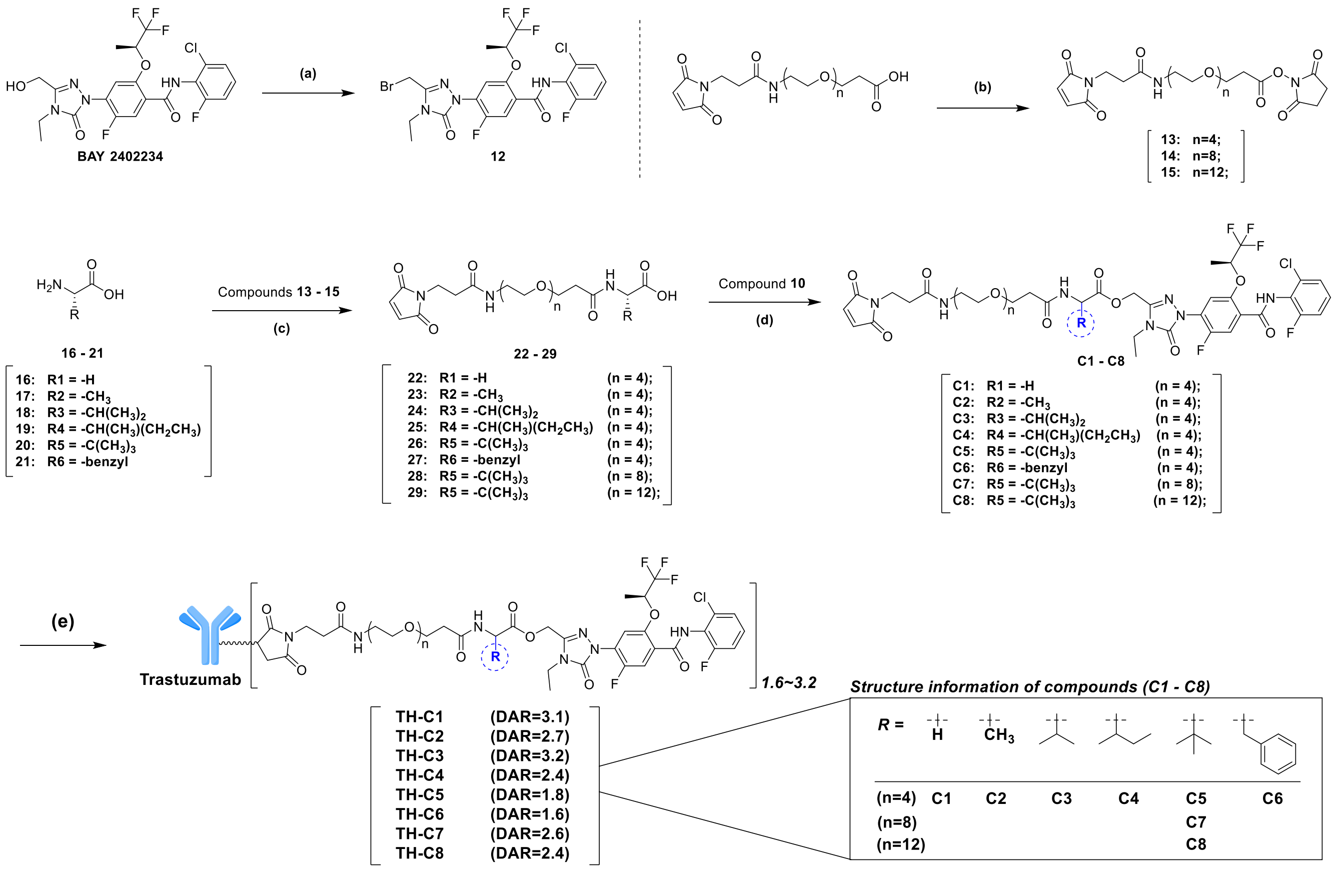

**Scheme S3** Design and synthesis of conjugates with stability-controllable linker (**TH-C1–C8**). Reagents and conditions: (a) PBr3, DCM, overnight, 81%–100%; (b) DIC, DCM, overnight; (c) DIPEA, DMF, 24 h, 49%–81%; (d) Potassium carbonate, DMF, 10–30 min, 43%–87%; (e) Trastuzumab, TCEP, NAC, Buffer.

*5.2.3.1. Synthesis of intermediates (**13–15**).* To a solution of Mal–PEG4–propionic acid (1.0 g, 2.4 mmol) in anhydrous DMF was added *N*,*N'*-Diisopropylcarbodiimide (DIC) (454 mg, 3.6 mmol) and *N*-Hydroxymaleimide (NHS) (332 mg, 2.9 mmol) at room temperature. The mixture continued to be stirred overnight or until the starting material disappeared completely (monitored by TLC). After evaporation of the solvent under vacuum, the crude product was purified by flash chromatography on silica gel (dichloromethane/methanol = 100:1, *v/v*) to give **13** as a semi-transparent solid (841 mg, 71.8% yield) that was directly used in the next step without further purification. **14** and **15** were synthesized in the same way as **13** or purchased directly on the market.

*5.2.3.2. Synthesis of (1-(2,5-dioxo-2,5-dihydro-1H-pyrrol-1-yl)-3-oxo-7,10,13,16-tetraoxa-4-azanonadecan-19-oyl)glycine (**22**).* To a solution of **13** (200 mg, 0.39 mmol) in anhydrous DMF was added **16** (Gly) (44 mg, 0.58 mmol) and DIPEA (100 mg, 0.78 mmol) at room temperature. The mixture

continued to be stirred overnight or until the starting material disappeared completely. After filtration of insoluble solid materials, the crude product was obtained by evaporation of the solution under vacuum and then purified by chromatography on silica gel (dichloromethane/methanol = 5:1, *v/v*) to give **22** as pale yellow oil (79 mg, 42.8% yield). $^{1}$H-NMR (400 MHz, $CDCl_3$): $\delta$ 6.84 (s, 2H), 3.92 (s, 2H), 3.78 (m, 4H), 3.64 (m, 14H), 3.52 (t, *J*=3.6, 2H), 2.54 (t, *J*=4.1, *J*=4.6, 2H), 2.49 (t, 2H). MS (ESI+) *m/z*: 474.21 $[M+H]^+$.

*5.2.3.3. Synthesis of (1-(2,5-dioxo-2,5-dihydro-1H-pyrrol-1-yl)-3-oxo-7,10,13,16-tetraoxa-4-azanonadecan-19-oyl)-L-alanine (23).* To a solution of **13** (87 mg, 0.168 mmol) in anhydrous DMF was added **17** (Ala) (10 mg, 0.11 mmol) and DIPEA (29 mg, 0.224 mmol) at room temperature. The mixture continued to be stirred overnight or until the starting material disappeared completely. After evaporation of the solvent under vacuum, the crude product was purified by chromatography on silica gel (dichloromethane/methanol = 5:1, *v/v*) to give (**23**) as a semi-transparent solid (25 mg, 45.7% yield). $^{1}$H-NMR (400 MHz, $CDCl_3$): $\delta$ 7.31 (s, 1H), 6.79 (s, 1H), 6.71 (s, 2H), 4.56 (m, 1H), 3.84 (t, *J*=5.1, 2H), 3.74 (m, 2H), 3.65 (m, 12H), 3.58 (m, 2H), 3.42 (m, 2H), 2.53 (q, *J*=4.8, 4H), 1.44 (m, 3H). HR-MS (ESI+) *m/z*: 488.2230 $[M+H]^+$; 510.2058 $[M+Na]^+$; 486.2093 $[M-H]^-$.

*5.2.3.4. Synthesis of (1-(2,5-dioxo-2,5-dihydro-1H-pyrrol-1-yl)-3-oxo-7,10,13,16-tetraoxa-4-azanonadecan-19-oyl)-L-valine (24).* To a solution of **13** (400 mg, 0.78 mmol) in anhydrous DMF was added **18** (Val) (137 mg, 1.2 mmol) and DIPEA (202 mg, 1.6 mmol) at room temperature. The mixture continued to be stirred overnight or until the starting material disappeared completely. After filtration of insoluble solid materials, the crude product was obtained by evaporation of the solution under vacuum and then purified by chromatography on silica gel (dichloromethane/methanol = 5:1, *v/v*) to give **24** as semi-transparent oil (197 mg, 49.1% yield). $^{1}$H-NMR (400 MHz, $CDCl_3$): $\delta$ 6.84 (s, 2H), 4.36 (d, J=3.7, 1H), 3.79 (t, *J*=4.6, 2H), 3.75 (m, 2H), 3.65 (m, 10H), 3.62 (m, 2H), 3.52 (t, *J*=3.6, 2H), 3.37 (s, 2H), 2.70 (s, 1H), 2.59 (m, 1H), 2.53 (m, 1H), 2.48 (t, *J*=4.6, 2H), 2.19 (m, 1H), 0.99 (t, *J*=4.8, 6H). HR-MS ($ESI^+$) *m/z*: 516.2548 $[M+H]^+$; 538.2371 $[M+Na]^+$.

*5.2.3.5. Synthesis of (1-(2,5-dioxo-2,5-dihydro-1H-pyrrol-1-yl)-3-oxo-7,10,13,16-tetraoxa-4-azanonadecan-19-oyl)-L-alloisoleucine (25).* To a solution of **13** (200 mg, 0.39 mmol) in anhydrous DMF was added **19** (Ile) (77 mg, 0.58 mmol) and DIPEA (100 mg, 0.78 mmol) at room temperature. The mixture continued to be stirred overnight or until the starting material disappeared completely. After filtration of insoluble solid materials, the crude product was obtained by evaporation of the solution under vacuum and then purified by chromatography on silica gel (dichloromethane/methanol = 5:1, *v/v*) to give

**25** as a semi-transparent solid (89 mg, 43.2% yield). $^1$H-NMR (400 MHz, $CDCl_3$): *δ* 7.12 (d, J=5.7, 1H), 6.76 (s, 1H), 6.71 (s, 2H), 4.56 (dd, 1H), 3.84 (t, J=4.8, 2H), 3.74 (m, 2H), 3.65 (m, 12H), 3.59 (m, 2H), 3.42 (m, 2H), 2.54 (dt, 4H), 1.94 (m, 1H), 1.52 (m, 1H), 1.21 (m, 1H), 0.94 (m, 6H). HR-MS (ESI+) *m/z*: 530.2707 $[M+H]^+$; 552.2529 $[M+Na]^+$.

*5.2.3.6. Synthesis of (S)-2-(tert-butyl)-22-(2,5-dioxo-2,5-dihydro-1H-pyrrol-1-yl)-4,20-dioxo-7,10,13,16-tetraoxa-3,19-diazadocosanoic acid (**26**).* To a solution of **13** (200 mg, 0.39 mmol) in anhydrous DMF was added **20** (Tle) (77 mg, 0.58 mmol) and DIPEA (100 mg, 0.78 mmol) at room temperature. The mixture continued to be stirred overnight or until the starting material disappeared completely. After filtration of insoluble solid materials, the crude product was obtained by evaporation of the solution under vacuum and then purified by chromatography on silica gel (dichloromethane/methanol = 5:1, *v/v*) to give **26** as semi-transparent oil (123 mg, 59.6% yield). $^1$H-NMR (400 MHz, $CDCl_3$): *δ* 7.12 (d, J=6.3, 1H), 6.83 (m, 1H), 6.71 (s, 2H), 4.47 (d, *J*=6.2, 1H), 3.84 (t, *J*=4.8, 2H), 3.75 (m, 2H), 3.65 (m, 12H), 3.58 (m, 2H), 3.42 (m, 2H), 2.54 (m, 4H), 1.02 (s, 9H). HR-MS ($ESI^+$) *m/z*: 530.2706 $[M+H]^+$; 552.2525 $[M+Na]^+$.

*5.2.3.7. Synthesis of (S)-22-(2,5-dioxo-2,5-dihydro-1H-pyrrol-1-yl)-4,20-dioxo-2-phenyl-7,10,13,16-tetraoxa-3,19-diazadocosanoic acid (1-(2,5-dioxo-2,5-dihydro-1H-pyrrol-1-yl)-3-oxo-7,10,13,16-tetraoxa-4-azanonadecan-19-oyl)-L-phenylalanine (**27**).* To a solution of **13** (200 mg, 0.39 mmol) in anhydrous DMF was added **21** (Phe) (97 mg, 0.58 mmol) and DIPEA (100 mg, 0.78 mmol) at room temperature. The mixture continued to be stirred overnight or until the starting material disappeared completely. After filtration of insoluble solid materials, the crude product was obtained by evaporation of the solution under vacuum and then purified by chromatography on silica gel (dichloromethane/methanol = 5:1, *v/v*) to give **27** as semi-transparent oil (109 mg, 49.7% yield). $^1$H-NMR (400 MHz, $CDCl_3$): *δ* 7.28 (m, 1H), 7.21 (m, 4H), 6.69 (s, 2H), 4.86 (m, 1H), 3.82 (t, *J*=3.8, 2H), 3.68 (m, 1H), 3.56 (m, 16H), 3.39 (m, 2H), 3.22 (dd, 1H), 3.13 (dd, 1H), 2.50 (m, 4H). HR-MS ($ESI^+$) *m/z*: 564.2552 $[M+H]^+$; 586.2368 $[M+Na]^+$.

*5.2.3.8. Synthesis of (S)-2-(tert-butyl)-34-(2,5-dioxo-2,5-dihydro-1H-pyrrol-1-yl)-4,32-dioxo-7,10,13,16,19,22,25,28-octaoxa-3,31-diazatetratriacontanoic acid (1-(2,5-dioxo-2,5-dihydro-1H-pyrrol-1-yl)-3-oxo-7,10,13,16,19,22,25,28-octaoxa-4-azahentriacontan-31-oyl)-L-phenylalanine (**28**).* To a solution of **14** (200 mg, 0.29 mmol) in anhydrous DMF was added **20** (Tle) (57 mg, 0.44 mmol) and DIPEA (75 mg, 0.58 mmol) at room temperature. The mixture continued to be stirred overnight or until the starting material disappeared completely. After filtration of insoluble solid materials, the crude product was obtained by evaporation of the solution under vacuum and then purified by chromatography

on silica gel (dichloromethane/methanol = 5:1, *v/v*) to give **28** as semi-transparent oil (109 mg, 53.2% yield). $^{1}$H-NMR (400 MHz, $CDCl_3$): *δ* 7.09 (d, *J*=6.2, 1H), 6.70 (s, 1H), 6.63 (s, 1H), 4.45 (d, *J*=6.2, 1H), 3.84 (t, *J*=4.8, 2H), 3.75 (m, 2H), 3.66 (m, 29H), 3.55 (t, *J*=3.4, 2H), 3.42 (q, *J*=3.5, 2H), 2.54 (t, *J*=4.7, 4H), 1.02 (s, 9H). HR-MS (ESI$^{+}$) *m/z*: 706.3757 $[M+H]^{+}$; 728.3576 $[M+Na]^{+}$; 353.6923 $[M+2H]^{2+}$.

*5.2.3.9. Synthesis of (S)-2-(tert-butyl)-46-(2,5-dioxo-2,5-dihydro-1H-pyrrol-1-yl)-4,44-dioxo-7,10,13,16,19,22,25,28,31,34,37,40-dodecaoxa-3,43-diazahexatetracontanoic acid (**29**).* To a solution of **15** (150 mg, 0.17 mmol) in anhydrous DMF was added **20** (Tle) (34 mg, 0.26 mmol) and DIPEA (45 mg, 0.35 mmol) at room temperature. The mixture continued to be stirred overnight or until the starting material disappeared completely. After filtration of insoluble solid materials, the crude product was obtained by evaporation of the solution under vacuum and then purified by chromatography on silica gel (dichloromethane/methanol = 5:1, *v/v*) to give **29** as semi-transparent oil (72 mg, 47.2% yield). $^{1}$H-NMR (400 MHz, CDOD): *δ* 6.82 (s, 2H), 4.30 (m, 1H), 3.77 (t, *J*=4.6, 2H), 3.74 (m, 2H), 3.63 (m, 48H), 3.50 (t, *J*=3.6, 2H), 2.68 (s, 2H), 2.58 (m, 1H), 2.51 (m, 1H), 2.46 (t, *J*=4.6, 2H), 1.02 (s, 9H). HR-MS (ESI$^{+}$) *m/z*: 904.4619 $[M+Na]^{+}$; 441.7445 $[M+2H]^{2+}$.

*5.2.3.10. Synthesis of (S)-(1-(4-((2-chloro-6-fluorophenyl)carbamoyl)-2-fluoro-5-((1,1,1-trifluoropropan-2-yl)oxy)phenyl)-4-ethyl-5-oxo-4,5-dihydro-1H-1,2,4-triazol-3-yl)methyl (1-(2,5-dioxo-2,5-dihydro-1H-pyrrol-1-yl)-3-oxo-7,10,13,16-tetraoxa-4-azanonadecan-19-oyl)glycinate (**C1**).* To a solution of **22** (52 mg, 0.11 mmol) in anhydrous DMF was added **12** (30 mg, 0.051 mmol) and anhydrous potassium carbonate (34 mg, 0.25 mmol) at room temperature. The mixture continued to be stirred for 10 min or until the starting material disappeared completely. After filtration of insoluble solid materials, the crude product was obtained by evaporation of the solution under vacuum and then purified by chromatography on silica gel (dichloromethane/methanol = 20:1, *v/v*) to give **C1** as a semi-transparent solid (25 mg, 50.3% yield). $^{1}$H-NMR (400 MHz, $CD_3OD$): *δ* 7.73 (d, *J*=6.8, 1H), 7.57 (d, *J*=3.6,1H), 7.38 (m, 2H), 7.23 (m, 1H), 6.80 (s, 2H), 5.23 (m, 3H), 4.06 (s, 2H), 3.88 (q, *J*=4.8, 2H), 3.74 (m, 5H), 3.63 (m, 14H), 3.49 (t, *J*=3.6, 2H), 2.52 (t, *J*=4.1, 2H), 2.45 (t, *J*=4.6, 2H), 1.58 (d, *J*=4.3, 3H), 1.38 (m, 3H). HR-MS (ESI$^{+}$) *m/z*: 976.2913 $[M+H]^{+;}$998.2731 $[M+Na]^{+}$.

*5.2.3.11. Synthesis of (1-(4-((2-chloro-6-fluorophenyl)carbamoyl)-2-fluoro-5-(((S)-1,1,1-trifluoropropan-2-yl)oxy)phenyl)-4-ethyl-5-oxo-4,5-dihydro-1H-1,2,4-triazol-3-yl)methyl (1-(2,5-dioxo-2,5-dihydro-1H-pyrrol-1-yl)-3-oxo-7,10,13,16-tetraoxa-4-azanonadecan-19-oyl)-L-alaninate (**C2**).* To a solution of (**23**) (25 mg, 0.051 mmol) in anhydrous DMF was added **12** (25 mg, 0.043 mmol) and anhydrous potassium carbonate (18 mg, 0.13 mmol) at room temperature. The mixture continued to be

stirred for 10 min or until the starting material disappeared completely. After filtration of insoluble solid materials, the crude product was obtained by evaporation of the solution under vacuum and then purified by chromatography on silica gel (dichloromethane/methanol = 10:1, *v/v*) to give **C2** as an off-white powder (8 mg, 18.8% yield). $^1$H-NMR (400 MHz, $CD_3OD$): $\delta$ 7.75 (d, *J*=6.8, 1H), 7.60 (d, *J*=3.8, 1H), 7.40 (m, 2H), 7.26 (m, 2H), 6.83 (s, 1H), 5.26 (m, 2H), 5.20 (m, 2H), 4.49 (m, 2H), 3.90 (q, *J*=4.8, 2H), 3.78 (t, *J*=4.6, 2H), 3.74 (m, 2H), 3.63 (m, 12H), 3.51 (t, *J*=3.6, 2H), 2.51 (m, 2H), 2.48 (m, 2H), 1.58 (d, *J*=4.3, 3H), 1.46 (d, *J*=4.9, 2H), 1.41 (m, 4H). MS (ESI$^+$) *m/z*: 990.48 $[M+H]^+$.

*5.2.3.12. Synthesis of (1-(4-((2-chloro-6-fluorophenyl)carbamoyl)-2-fluoro-5-(((S)-1,1,1-trifluoropropan-2-yl)oxy)phenyl)-4-ethyl-5-oxo-4,5-dihydro-1H-1,2,4-triazol-3-yl)methyl (1-(2,5-dioxo-2,5-dihydro-1H-pyrrol-1-yl)-3-oxo-7,10,13,16-tetraoxa-4-azanonadecan-19-oyl)-L-valinate (**C3**).* To a solution of **24** (30 mg, 0.058 mmol) in anhydrous DMF was added **12** (31 mg, 0.053 mmol) and anhydrous potassium carbonate (24 mg, 0.174 mmol) at room temperature. The mixture continued to be stirred for 10 min or until the starting material disappeared completely. After filtration of insoluble solid materials, the crude product was obtained by evaporation of the solution under vacuum and then purified by chromatography on silica gel (dichloromethane/methanol = 10:1, *v/v*) to give **C3** as an off-white solid (47 mg, 86.9% yield). $^1$H-NMR (400 MHz, $CDCl_3$): $\delta$ 9.02 (s, 1H), 8.18 (d, *J*=7.5, 1H), 7.50 (d, *J*=3.7, 1H), 7.29 (m, 1H), 7.24 (m, 1H), 7.13 (m, 1H), 7.02 (d, J=5.5, 1H), 6.70 (s, 2H), 6.46 (s, 1H), 5.18 (d, *J*=9.0, 1H), 5.09 (d, *J*=9.0, 1H), 4.99 (m, 1H), 4.54 (dd, 1H), 3.85 (m, 4H), 3.75 (m, 2H), 3.64 (m, 12H), 3.54 (t, *J*=3.3, 2H), 3.42 (t, *J*=3.3, 2H), 2.55 (m, 2H), 2.52 (t, *J*=4.8, 2H), 2.21 (m, 1H), 1.66 (d, *J*=4.3, 3H), 1.41 (t, *J*=4.8, 3H), 0.98 (dd, 6H). HR-MS (ESI$^+$) *m/z*: 1018.3384 $[M+H]^{+;}$ 1040.3202 $[M+Na]^+$.

*5.2.3.13. Synthesis of (1-(4-((2-chloro-6-fluorophenyl)carbamoyl)-2-fluoro-5-(((S)-1,1,1-trifluoropropan-2-yl)oxy)phenyl)-4-ethyl-5-oxo-4,5-dihydro-1H-1,2,4-triazol-3-yl)methyl (1-(2,5-dioxo-2,5-dihydro-1H-pyrrol-1-yl)-3-oxo-7,10,13,16-tetraoxa-4-azanonadecan-19-oyl)-L-alloisoleucinate (**C4**).* To a solution of **25** (32 mg, 0.060 mmol) in anhydrous DMF was added **12** (32 mg, 0.055 mmol) and anhydrous potassium carbonate (23 mg, 0.16 mmol) at room temperature. The mixture continued to be stirred for 10 min or until the starting material disappeared completely. After filtration of insoluble solid materials, the crude product was obtained by evaporation of the solution under vacuum and then purified by chromatography on silica gel (dichloromethane/methanol = 10:1, *v/v*) to give **C4** as a semi-transparent solid (51 mg, 90.2% yield). $^1$H-NMR (400 MHz, $CDCl_3$): $\delta$ 9.02 (s, 1H), 8.18 (d, J=7.6, 1H), 7.50 (d, *J*=3.7, 1H), 7.29 (m, 1H), 7.24 (m, 1H), 7.13 (m, 1H), 6.97 (d, *J*=5.4, 1H), 6.70 (s, 2H), 6.28 (s, 1H), 5.19 (d, *J*=9.0, 1H), 5.08 (d, *J*=9.0, 1H), 4.98 (m, 1H), 4.58 (m, 1H), 3.85 (m, 4H), 3.74 (m, 2H), 3.65 (m, 8H), 3.62 (m, 4H), 3.54 (t, *J*=3.4, 2H), 3.42 (q, *J*=5.0, 2H), 2.55 (m, 4H), 1.90 (m, 1H), 1.66 (d,

*J*=4.3, 3H), 1.47 (m, 1H), 1.41 (t, *J*=4.8, 3H), 1.22 (m, 1H), 0.97 (d, *J*=4.6, 3H), 0.92 (t, *J*=4.9, 3H). HR-MS ($ESI^+$) *m/z*: 1032.3542 $[M+H]^{+;}$ 1054.3355 $[M+Na]^{+;}$ 516.6823 $[M+2H]^{2+}$.

*5.2.3.14. Synthesis of (1-(4-((2-chloro-6-fluorophenyl)carbamoyl)-2-fluoro-5-(((S)-1,1,1-trifluoropropan-2-yl)oxy)phenyl)-4-ethyl-5-oxo-4,5-dihydro-1H-1,2,4-triazol-3-yl)methyl (S)-2-(tert-butyl)-22-(2,5-dioxo-2,5-dihydro-1H-pyrrol-1-yl)-4,20-dioxo-7,10,13,16-tetraoxa-3,19-diazadocosanoate (**C5**).* To a solution of **26** (37 mg, 0.070 mmol) in anhydrous DMF was added **12** (34 mg, 0.058 mmol) and anhydrous potassium carbonate (25 mg, 0.18 mmol) at room temperature. The mixture continued to be stirred for 10 min or until the starting material disappeared completely. After filtration of insoluble solid materials, the crude product was obtained by evaporation of the solution under vacuum and then purified by chromatography on silica gel (dichloromethane/methanol = 10:1, *v/v*) to give **C5** as a semi-transparent solid (68 mg, 110% yield). $^1$H-NMR (400 MHz, $CDCl_3$): $\delta$ 9.02 (s, 1H), 8.18 (d, J=7.5, 1H), 7.50 (d, J=3.7, 1H), 7.29 (m, 1H), 7.24 (m, 1H), 7.13 (m, 1H), 7.08 (m, 1H), 6.70 (s, 2H), 6.23 (s, 1H), 5.19 (d, *J*=9.0, 1H), 5.06 (d, *J*=9.0, 1H), 4.98 (m, 1H), 4.42 (d, *J*=5.6, 1H), 3.88 (m, 2H), 3.84 (t, *J*=4.8, 2H), 3.74 (m, 2H), 3.66 (m, 8H), 3.62 (m, 4H), 3.54 (t, *J*=3.4, 2H), 3.42 (q, *J*=3.3, 2H), 2.54 (t, *J*=3.8, 2H), 2.52 (t, *J*=4.8, 2H), 1.78 (s, 1H), 1.66 (d, *J*=4.3, 3H), 1.41 (t, *J*=4.8, 3H), 1.03 (s, 9H). HR-MS ($ESI^+$) *m/z*: 1032.3537 $[M+H]^+$; 1054.3353 $[M+Na]^+$; 516.6820 $[M+2H]^{2+}$.

*5.2.3.15. Synthesis of (1-(4-((2-chloro-6-fluorophenyl)carbamoyl)-2-fluoro-5-(((S)-1,1,1-trifluoropropan-2-yl)oxy)phenyl)-4-ethyl-5-oxo-4,5-dihydro-1H-1,2,4-triazol-3-yl)methyl (1-(2,5-dioxo-2,5-dihydro-1H-pyrrol-1-yl)-3-oxo-7,10,13,16-tetraoxa-4-azanonadecan-19-oyl)-L-phenylalaninate (**C6**).* To a solution of **27** (35 mg, 0.062 mmol) in anhydrous DMF was added **12** (59 mg, 0.10 mmol) and anhydrous potassium carbonate (22 mg, 0.15 mmol) at room temperature. The mixture continued to be stirred for 10 min or until the starting material disappeared completely. After filtration of insoluble solid materials, the crude product was obtained by evaporation of the solution under vacuum and then purified by chromatography on silica gel (dichloromethane/methanol = 10:1, *v/v*) to give **C6** as a semi-transparent solid (64 mg, 96.7% yield). $^1$H-NMR (400 MHz, $CDCl_3$): $\delta$ 7.74 (d, *J*=6.8, 1H), 7.56 (d, *J*=3.8, 1H), 7.38 (m, 2H), 7.27 (m, 2H), 7.22 (m, 5H), 6.79 (s, 2H), 5.22 (m, 1H), 5.16 (d, *J*=1.9, 2H), 4.70 (m, 1H), 3.74 (m, 4H), 3.66 (m, 2H), 3.60 (m, 6H), 3.57 (m, 4H), 3.54 (m, 2H), 3.48 (m, 2H), 3.29 (m, 2H), 3.16 (m, 1H), 3.07 (m, 1H), 2.45 (m, 4H), 1.57 (d, *J*=4.3, 3H), 1.30 (t, *J*=4.8, 3H). HR-MS ($ESI^+$) *m/z*: 1066.3388 $[M+H]^+$; 1088.3101 $[M+Na]^{+;}$ 533.6740 $[M+2H]^{2+}$.

*5.2.3.16. Synthesis of (1-(4-((2-chloro-6-fluorophenyl)carbamoyl)-2-fluoro-5-(((S)-1,1,1-trifluoropropan-2-yl)oxy)phenyl)-4-ethyl-5-oxo-4,5-dihydro-1H-1,2,4-triazol-3-yl)methyl (S)-2-(tert-*

*butyl)-34-(2,5-dioxo-2,5-dihydro-1H-pyrrol-1-yl)-4,32-dioxo-7,10,13,16,19,22,25,28-octaoxa-3,31-diazatetratriacontano*ate ***(C7)***. To a solution of **28** (40 mg, 0.057 mmol) in anhydrous DMF was added **12** (30 mg, 0.052 mmol) and anhydrous potassium carbonate (24 mg, 0.17 mmol) at room temperature. The mixture continued to be stirred for 10 min or until the starting material disappeared completely. After filtration of insoluble solid materials, the crude product was obtained by evaporation of the solution under vacuum and then purified by chromatography on silica gel (dichloromethane/methanol = 10:1, *v/v*) to give **C7** as a semi-transparent solid (31 mg, 49.8% yield). $^{1}$H-NMR (400 MHz, $CDCl_3$): $\delta$ 9.02 (s, 1H), 8.18 (d, *J*=7.5, 1H), 7.50 (d, *J*=3.7, 1H), 7.29 (m, 1H), 7.24 (m, 1H), 7.12 (m, 2H), 7.08 6.70 (s, 2H), 6.52 (s, 1H), 5.19 (d, *J*=9.0, 1H), 5.06 (d, *J*=9.0, 1H), 4.98 (m, 1H), 4.40 (d, *J*=5.6, 1H), 3.86 (m, 2H), 3.84 (t, *J*=4.8, 2H), 3.75 (m, 2H), 3.67 (s, 4H), 3.64 (m, 24H), 3.53 (t, *J*=3.4, 2H), 3.42 (s, 2H), 2.53 (m, 4H), 1.66 (d, *J*=4.3, 3H), 1.41 (t, *J*=4.8, 3H), 1.03 (s, 9H). HR-MS (ESI$^{+}$) *m/z*: 1230.4387 [M+Na]$^{+;}$ 605.2338 [M+2H]$^{2+}$.

*5.2.3.17. Synthesis of (1-(4-((2-chloro-6-fluorophenyl)carbamoyl)-2-fluoro-5-(((S)-1,1,1-trifluoropropan-2-yl)oxy)phenyl)-4-ethyl-5-oxo-4,5-dihydro-1H-1,2,4-triazol-3-yl)methyl (S)-2-(tert-butyl)-46-(2,5-dioxo-2,5-dihydro-1H-pyrrol-1-yl)-4,44-dioxo-7,10,13,16,19,22,25,28,31,34,37,40-dodecaoxa-3,43-diazahexatetracontanoate **(C8)***. To a solution of **29** (48 mg, 0.055 mmol) in anhydrous DMF was added **12** (29 mg, 0.050 mmol) and anhydrous potassium carbonate (21 mg, 0.15 mmol) at room temperature. The mixture continued to be stirred for 10 min or until the starting material disappeared completely. After filtration of insoluble solid materials, the crude product was obtained by evaporation of the solution under vacuum and then purified by chromatography on silica gel (dichloromethane/methanol = 10:1, *v/v*) to give **C8** as semi-transparent oil (37 mg, 53.9% yield). $^{1}$H-NMR (400 MHz, DMSO): $\delta$ 10.07 (s, 1H), 8.15 (d, *J*=5.5, 1H), 8.01 (t, *J*=7.6, 1H), 7.61 (d, *J*=3.9, 1H), 7.57(d, *J*=6.5, 1H), 7.43 (m, 2H), 7.36 (m, 1H), 7.00 (s, 2H), 5.34 (m, 1H), 5.24 (d, *J*=9.1, 1H), 5.18 (d, *J*=9.1, 1H), 4.23 (d, *J*=5.5, 1H), 3.77 (m, 2H), 3.59 (m, 4H), 3.50 (m, 46H), 3.14 (q, *J*=3.8, 2H), 2.33 (t, *J*=4.9, 2H), 1.45 (d, *J*=4.2, 3H), 1.27 (t, *J*=4.8, 3H), 0.97 (s, 9H). HR-MS (ESI$^{+}$) *m/z*: 703.7775 [M+Na+H]$^{2+}$.

*5.2.3.18. Synthesis of ADCs **TH-C1–C8***. Compounds **C1**–**C8** were conjugated to Trastuzumab by *Standard Procedure A* respectively to prepare **TH-C1**–**C8**.

*5.2.3.19. Synthesis of ADCs **TH-C8H** and **HG-C3***. Compound **C8** was conjugated to Trastuzumab by *Standard Procedure B* to prepare **TH-C8H**, which was used for the antitumor study. While, compound **C3** was conjugated to human IgG1k antibody by *Standard Procedure A* to prepare **HG-C3**, which was

used for the antiviral study.

*5.2.4. Quality control*

HPLC was used to determine the purity of small molecule compounds. The DAR of conjugates was analyzed using hydrophobic interaction chromatography (HIC) or UPLC–Q-TOF-MS (Agilent, 6500 series Q-TOF, Canada). The aggregation level was determined by size exclusion chromatography (SEC).

*HPLC analysis:* HPLC analysis was conducted using an Agilent 1260 infinity module equipped with a reversed-phase column (Agilent Eclipse XDB-C18 column, 5 μm, 250 mm × 4.6 mm). The mobile phase consisted of a 0.1% formic acid solution and acetonitrile in a 1:1 ratio with isocratic elution. The column temperature was maintained at 30 °C, with a detection wavelength of 254 nm with a sample injection volume of 10 μL per needle. Chromatography peaks were automatically integrated by software integrated into the workstation, and peak area normalization methods were carried out to calculate the purity. Chromatographic column: Agilent Eclipse XDB-C18 column (5 μm, 250 mm × 4.6 mm)。

*HIC analysis:* HIC analysis was carried out on a TSK-gel Butyl-NPR chromatography column (2.5 μm, 4.6 mm × 3.5 cm, TOSOH Bioscience) with linear gradient elution. The mobile phases consisted of (A) a solution containing 25 mmol/L sodium phosphate and 1.8 mol/L ammonium sulfate in water and (B) a solution containing 25 mmol/L sodium phosphate in water containing 20% isopropanol. The gradient ranged from 0% to 100% mobile phase B over 18.00 min, followed by re-equilibration from 18.00 to18.01 min with 100% mobile phase A, after which the mobile phase ratio remained constant until the entire method was completed. The column temperature of this method was maintained at 30 °C, with a flow rate of 0.8 mL/min, and detection was performed at a wavelength of 280 nm. The DAR of conjugates was determined by calculating from the sum of the weighted ratios of each peak area under the curve multiplied by corresponding peak drug loading through HIC analysis.

*UPLC–Q-TOF-MS analysis:* UPLC–Q-TOF-MS analysis was conducted using an Agilent 6550 LC–Q-TOF MS spectrometer equipped with a TSK UP-SW3000 column (4.6 mm×150 mm, 2.5 μm), and eluted with 200 mmol/L ammonium acetate solution at a flow rate of 0.2 mL/min. The Agilent MassHunter Bioconfirm software (deconvolution for protein, Agilent technology) was used to deconvolute the multiple charged peaks of ADCs.

*SEC analysis:* SEC analysis was carried out on a TSK gel butyl-NPR column (4.6 × 35 mm, 2.5 μm) maintained at 30 °C. Mobile phase A consisted of 1.5 mol/L ammonium sulfate and 25 mmol/L TRIS-HCl at pH 8.0, while mobile phase B was 25 mmol/L TRIS-HCl and 5% isopropyl alcohol (*v/v*) at pH 8.0. Separations were executed using a 0–100% linear gradient procedure of mobile phase B over 20 min, with detection at a wavelength of 280 nm. Sample injections ranged from approximately 5 μg to 15 μg. All samples were filtrated using a 0.22 μm needle filter.

*5.2.5. Stability study*

*The stability assays of small molecules (**C1–C6**) in PBS:* The calculated amounts of compounds **C1**–**C6** were respectively dissolved in organic solvent (DMA, or DMF). Then, an excess of 1.0 mg/mL aqueous solution of NAC was added to quench the maleimide joint. The solution was then stood at room temperature for 30 min, followed by the addition of 9 times the volume of Dulbecco's phosphate buffer (pH = 7.4) to achieve final concentrations ranging from 0.05 to 0.15 mg/mL. The samples were incubated at 37 °C. Aliquots (100–200μL) were collected at subsequent time points (0, 24, 48, 96, 168, 240, 336, and 504 h) and stored at −80 °C. Upon completion of the sampling, the release of **BAY** of each sample was determined by HPLC.

*The stability assays of ADCs (**TH-C2–C5**) in plasma:* We investigated the plasma stability of representative conjugates (**TH-C2**–**C5**) by diluting them into a mixture solution containing 0.03 mg/mL of 50% human plasma and incubating at 37 °C in a constant temperature incubator. Aliquots (100 μL) were collected at subsequent time points (0, 6, 24, 48, 96, 168, 240, and 336 h), and methanol (500 μL) was added to the samples. After centrifugation to extract the supernatant, LC–MS analysis was used to quantify the released **BAY**. After sampling was completed, the release of **BAY** for each sample was measured by UPLC–Q-TOF-MS.

*5.2.6. In vitro studies*

*Affinity assay:* The affinity of the ADC for HER2 antigen was measured on SKOV3 cells. To extract the supernatant, a specific volume of FACS solution (containing $3 \times 10^5$–$5 \times 10^5$ cells) was transferred to a 1.5 mL centrifuge tube and centrifuged at 7200 rpm for 1 min (TOMY MX-307 telescopic micro-refrigerated centrifuge). Before adding 100 μL of the ADC sample to the cells, it was diluted to a concentration of 40 μg/mL with PBS and kept on ice. The mixture was then incubated at 4 °C for 30 min. Subsequently, (Cytomics FC 500, Beckman Coulter, USA) was performed to calculate the affinity. SKOV3 cells (or NIH-3T3 cells) without treatment were served as a negative control.

*In vitro cell imaging:* SKOV3 cells ($5 \times 10^4$ cells/well) were cultured in new confocal dishes and subsequently incubated with ADC (FITC labeled **TH-C8H**, 1 μmol/L) for 2 d at 37 °C. Free ADCs were removed by washing the samples twice with a pre-cooled PBS solution. Lysozyme (50 nmol/L per well) was added to the samples and was shielded from light for 30 min at 4 °C. Following this, the samples were washed twice with a pre-cooled PBS solution. Hoechast stain (100 nmol/L per well) was added to the samples and kept in the dark for 20 min at 25 °C and then were washed once with a pre-cooled PBS solution. Fluorescence images were captured using a confocal laser scanning microscope (Zeiss LSM 510, Zeiss, Oberko, Germany).

*Cell proliferation assay:* The HER2-positive ovarian cancer cell line SKOV3, breast cancer cell line SKBR3, gastric cancer cell line NCI-N87 and HER2-negative mouse embryonic cells NIH-3T3, canine renal cells MDCK were selected to detect the cytotoxicity of ADCs. Cells were seeded in 96-well plates ($8 \times 10^3$ cells per well) with 100 μL of complete medium and adhered overnight at 37 °C in a humidified atmosphere of 5% $CO_2$. Subsequently, the cells were incubated for 24 to 48 h depending on the cell status. Samples (either ADCs or small molecules at concentrations ranging from 100 to 0.015 nmol/L) were diluted with 3.0-fold to create multiple concentration gradients and were added to the supernatant. Each concentration point contains 3 replicates. After incubation under growth conditions for another 96 h (144 h for NCI-N87), the cells were removed from the incubator and equilibrated at 25 °C for 30 min. Then 100 μL of CellTiter-Glo Luminescent Solution (Promega, Cat. No. G7570) was added to each well. After shaking at 650 rpm for 8 min, the plates were placed at room temperature for an additional 5 min. Luminescence was measured using a GloMax® Navigator system with an integration time of 1 s per well. $IC_{50}$ values were calculated using GraphPad Prism 8.0 (GraphPad Software).

*Cell co-culture assay*: $HER2^+$ SKBR3 cells, $HER2^-$ MDA-MB-231 cells and co-culture (SKBR3 + MDA-MB-231) cells were seeded in a 6-well plate at $2 \times 10^5$ cells, with 2 mL culture medium/well. $HER2^+$ NCI-N87 cells, $HER2^-$ MCF-7 cells and co-culture (NCI-N87 + MCF-7) cells were seeded in a 6-well plate at $3 \times 10^5$ cells, with 2 mL culture medium/well. After overnight incubation, the supernatant was removed from the plate, then **BAY** (5 nmol/L), Trastuzumab (100 nmol/L), or **TH-C8H** diluent (5 nmol/L) was added. After 5 d of incubation, the surviving cells were detached from the plate, and the number of cells in each well was determined using a cell counter. To determine the ratio of SKBR3/MDA-MB-231 and NCI-N87/MCF-7 cells of the total viable cells, the cells were stained with anti-HER2 antibody (ab264541, Abcam, MA, USA) and incubated for 60 min in the dark. Subsequently, the cells were washed 3 times by centrifugation at 400 g for 5 min. Then, cells were incubated with fluorochrome labeled secondary antibody in 3% BSA/PBS for 30 min at room temperature. After washing, fluorescent signals of $1 \times 10^4$ stained cells were measured using a flow cytometric analysis. Based on the number and ratio of HER2-positive and HER2-negative cells in each treatment well, the number of SKBR3/MDA-MB-231 and NCI-N87/MCF-7 cells were calculated.

*Cell cycle arrest:* SKOV3 cells were inoculated into a 12-well plate ($1.0 \times 10^5$ cells /mL), and incubated at 37 °C for 24 h under appropriate incubation conditions. The ADC was diluted with medium to concentrations of 1, 3, and 10 nmol/L, and added into the corresponding wells and continued to incubate for 72 h. Each concentration point contains 3 replicates. The cells were collected and washed with pre-cooled PBS (500 μL) followed by Rinse lightly with pre-cooled 70% ethanol. The mixture was fixed at

4 °C for 30 min; centrifuged at 1000 rpm for 5 min. Then 500 μL of pre-cooled PBS was added to resuspension and washed the cells. Each sample was added with propidium iodide (PI) staining solution (0.5 mL per tube) and RNase (10 uL), and incubated at 25 °C for 30 min in the dark. Flow cytometric analysis was performed to assess the effects of ADC on cell cycle arrest.

*Apoptosis analysis:* SKOV3 cells were seeded in 12-well plates ($1.0 \times 10^5$ cells/mL) with 100 μL of complete medium per well and adhered for 24 h at 37 °C in a humidified atmosphere of 5% $CO_2$. The ADC was diluted with medium to concentrations of 1, 3, and 10 nmol/L, and added into the corresponding plates. Each concentration point contains 3 replicates. Subsequently, the plates were placed and incubated for 96 h. The cell culture solution was inhaled into a suitable centrifuge tube, the adherent cells were washed with PBS, and an appropriate amount of pancreatic enzyme digestion solution was added for cell digestion. The collected cell culture medium was added, and the cells were gently pipetted to dislodge, transferred to a centrifuge tube, and centrifuged at 1000 g for 5 min. The supernatant was discarded then the cells were collected and gently resuspended in PBS. Annexin V-FITC (5 μL) and PI (10 μL) staining solutions were added sequentially and incubated for 20 min at 25 °C in the dark. Flow cytometric analysis was performed to evaluate the cell apoptosis.

*Virus stock:* The SARS-CoV-2 prototype, Delta variant, Omicron BA.5 variant and XBB1.9.2.1 variant were passaged on Vero cells (#CCL-81, ATCC), respectively. Viral stocks were prepared in Vero cells with DMEM (#11995) supplemented with 2% FBS (#10270106), 5 μg/mL TPCK-trypsin (#T1426), Penicillin (100 U/mL)–Streptomycin (100 μg/mL) (#15140-122) and 30 mmol/L $MgCl_2$. All these reagents were purchased from GIBCO, SIGMA-ALDRICH and Invitrogen. The viruses were subsequently harvested and stored in an ultra-low temperature refrigerator. The titers were determined by means of plaque assay in Vero cells.

*Evaluation for the antiviral efficacy of ADC in vitro:* The ADC (**HG-C3**) was diluted by a gradient in a 96-well plate and co-incubated with SARS-CoV-2 for 1 h (100 $TCID_{50}$ per well). Subsequently, the mixture was added to a 96-well plate seeded with Vero cells for 1 h another incubation. Each concentration point contains 4 replicates. Then, the mixture was renewed with a fresh culture medium. Three days post-incubation, the antiviral deficiency of small molecule drug was evaluated by measuring the inhibition of cytopathic effect (CPE). The values of 100, 75, 50, 25, and 0 indicated the percentage of CPE in each well, respectively.

*Detection of viral RNA levels:* Viral RNA was extracted by using the QIAamp Viral RNA Mini kit (#52906, Qiagen) according to the manufacturer's instructions. RT-PCR was conducted by using the SLAN-96S Real-Time System (Hongshi, Shanghai, China) with a SARS-CoV-2 RT-PCR Kit from

Wantai (Beijing, China). The relative viral RNA of SARS-CoV-2 ORF1ab and NP gene were determined using primers pairs and probes shown in the kit instruction. Viral RNA copies were expressed on a log10 scale after normalized to the standard curve obtained by using ten-fold dilutions of a SARS-CoV-2 stock.

*5.2.7. In vivo antitumor activity of ADC* ***TH-C8H***

*Experimental animal and methods:* NOD/SCID mice were purchased from SiPeiFu company (Beijing, China) and ranged from 6 to 8 weeks of age, with an average weight of approximately 20 g. All the mice were housed in specific pathogen-free conditions, and all procedures were performed under guidelines approved by Institutional Ethics Committee of Military Medical Science (IACUC-DWZX-2022-649). The mice were injected subcutaneously with gastric cancer NCI-N87 cells ($1 \times 10^7$ cells) suspended in 0.1 mL of DMEM. The treatment started when the mean tumor volume of the gastric cancer xenograft model reached approximately 200 $mm^3$. The mice were randomly divided into six groups, with no less than five mice in each group. Then the mice were given vehicle (PBS), [Trastuzumab (15 mg/kg) + **BAY** (234 μg/kg)], **TH-C8H** (5 mg/kg), **TH-C8H** (15 mg/kg), **RSL**3 (10mg/kg), and [**RSL3** (10mg/kg) + **TH-C8H** (5 mg/kg)], on Days 0, 4, 8, 12, 16, and 20, respectively. [In the sample above, 15 mg of **TH-C8H** contains the equivalent of 234 μg of **BAY**, conversion formula: (15 mg/150,000) * 521 * 4.5 *1000 = 234 μg]. The mice were monitored once every three days for body weight and tumor size. Tumor volume was calculated using the formula: $TV = a \times b^2/2$, where "a" and "b" are the long and short diameters of a tumor, respectively. Euthanasia and tumor collection were performed when tumor volumes reached roughly 1500 $mm^3$. Tumor growth inhibition value (TGI)% = $[1 - RTV(Treated)/RTV(Control)] \times 100\%$. All experimental procedures were executed according to the protocols approved by Institutional Ethics Committee of Military Medical Science (IACUC-DWZX-2022-649). *Evaluation of malondialdehyde (MDA) and 4-hydroxynonenal (4-HNE) level:* The relative MDA concentration in cell or tumor lysates was assessed using a Lipid Peroxidation (MDA) Assay Kit (ab118970, Abcam), according to the manufacturer’s instructions. This assay measures the MDA reaction with thiobarbituric acid (TBA) that generatea an MDA-TBA adduct in a sample. The MDA-TBA adduct can be quantified colorimetrically (OD = 532 nm). Lipid Peroxidation (4-HNE) Assay Kit (Abcam, ab238538) was used to evaluate the concentration of 4-HNE according to the manufacturer’s protocol.

*5.2.8. In vivo antiviral activity of ADC* ***TH-C3***

*Experimental animal and biosafety:* The Golden Syrian Hamster was raised in specific pathogen-free animal feeding facilities. All experiments with infectious SARS-CoV-2 were performed in the biosafety level 3 (BSL-3) and animal biosafety level 3 (ABSL-3) facilities. Our staff wear powered air-purifying

respirators that filter the air and disposable coveralls when they culture the virus and handle animals that are in isolation. The researchers are disinfected before they leave the room and then shower on exiting the facility. All facilities, procedures, training records, safety drills, and inventory records are subject to periodic inspections and ongoing oversight by the institutional biosafety officers who consult frequently with the facility managers. All experimental procedures were executed according to the protocols approved by the Medical Ethics Committee of joint Institute of Virology of Shantou University and The University of Hong Kong (SUCM2021-112).

*Virus inoculation, ADC treatment and sample collection:* The hamsters were randomly divided into four groups with no less than four hamsters in each group. The hamsters were anesthetized by isoflurane (#R510-22, RWD Life Science) and nasally inoculated with indicated doses of SARS-CoV-2 diluted in 200 μL of PBS (#10010031, GIBCO). In the treatment of SARS-CoV-2 infection, the hamsters were anesthetized by isoflurane and nasally inoculated with ADC (10 mg/kg) diluted in 200 μL of PBS at 2 h after virus inoculation. The body weight of these hamsters was measured by electronic balance. The hamsters were euthanized at the indicated time points, and respiratory tract tissues, including turbinate, trachea and lung, were collected to detect of viral RNA levels and cytokines.

*Histopathological studies:* For pathological analysis, lung tissues were fixed in formalin for over 72 h, dehydrated and then embedded in paraffin wax. The wax block of lung tissues was cut into 4 μm sections for several pathological staining and analysis. Hematoxylin and eosin (H&E) staining was employed for the analysis of general lung pathogenic lesions, including pulmonary edema, consolidation and inflammation. The standards for a pathological score of lung tissues in this study are derived from our previous study in a hamster model. Comprehensive pathological scores of lung sections were performed according to the degree of lung lesions, including alveolar septum hyperplasia, consolidation and impairment of alveolar structure, fluid exudation, mucus suppository, thrombus, inflammation recruitment and infiltration of immune cells in each individual lung lobes. For each hamster, three or four lung lobes were employed for evaluation of the comprehensive pathological score. In brief, the H&E staining results for each lung lobe were analyzed for the severity of pathological change. The pathological scores were categorized as follows: (a) Alveolar septum thickening and consolidation; (b) Hemorrhage, exudation, pulmonary edema and mucous; (c) Recruitment and infiltration of inflammatory immune cells. For each issue, a score related to the severity: "0" indicates no pathological change was observed, "1" indicates moderate pathological change, "2" indicates mild pathological change, "3" indicates severe pathological change, and "4" indicates very severe pathological change. Consequently, the scores of such three issues were added as the comprehensive pathological score of a lung lobe, with the average

comprehensive pathological score of the lobes indicating the severity of lung pathogenesis in the evaluated hamster. The images of whole lung lobes were screened by a high-throughput screening microscope system (EVOS M7000, Invitrogen of Thermo Fisher Scientific).

*Evaluation of cytokine storm:* To evaluate the severity of cytokine storm in hamsters, the supernatants of lung tissue homogenates were detected by the hamster IL-6 and IFN-$\gamma$ ELISA Kit, (Cusabio, USA).

*5.2.9. Statistical analyses*

All *in vitro*/*in vivo* activity tests included duplicate samples. The $IC_{50}$ values of all ADCs and small molecule compounds were expressed as the mean ± standard deviation (SD). For statistical analysis, the GraphPad Prism 8 software was used for two-way ANOVA or unpaired t-test (*$P < 0.05$, **$P < 0.001$; ***$P < 0.0003$; ****$P < 0.0001$; *ns* nonsignificant), and comments were given in the corresponding sections. All data points are normally distributed. In conducting the experiment and evaluating the data, the researchers were not blinded to the distribution of treatment groups. Sufficient sample size was taken into consideration to ensure adequate power. Our *in vivo* studies comply with animal guidelines, including ARRIVE guidelines and MIBBI guidelines.

*5.2.10. Data availability*

Data is available on request from the authors.

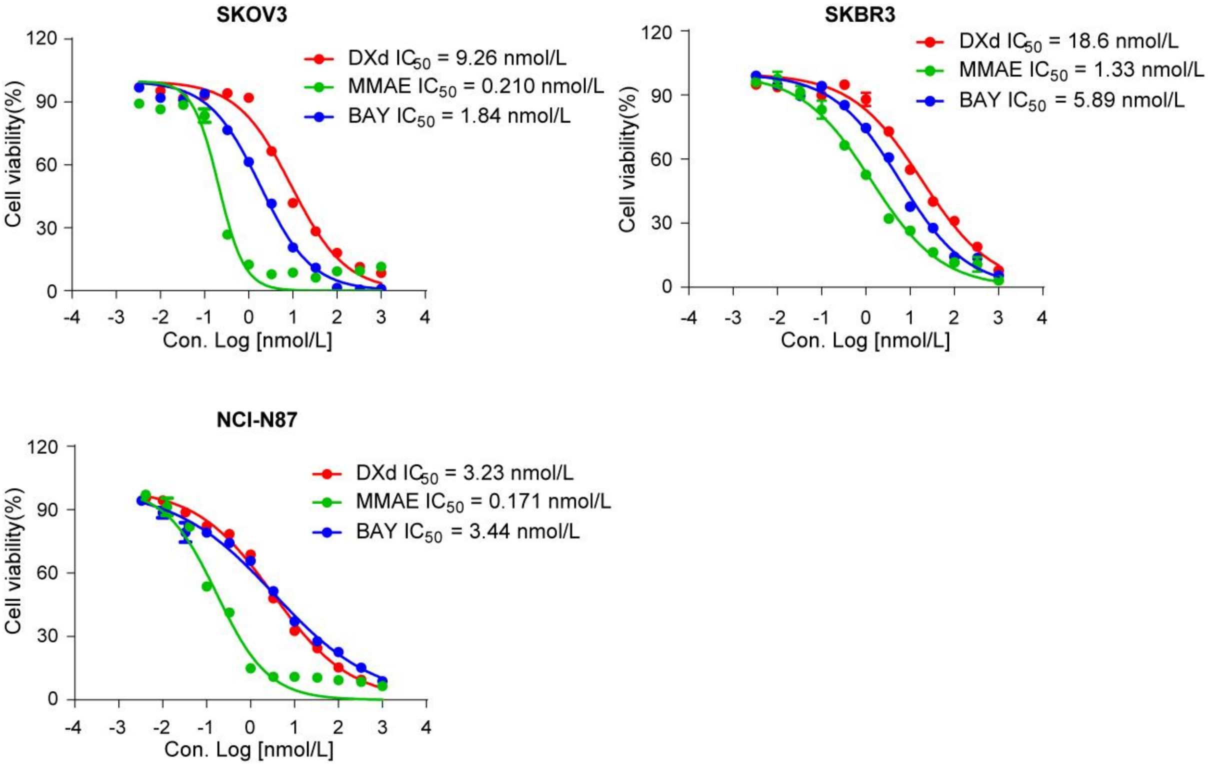


**Figure S1** Cytotoxic activity test data of **MMAE**, **DXd** and **BAY**.

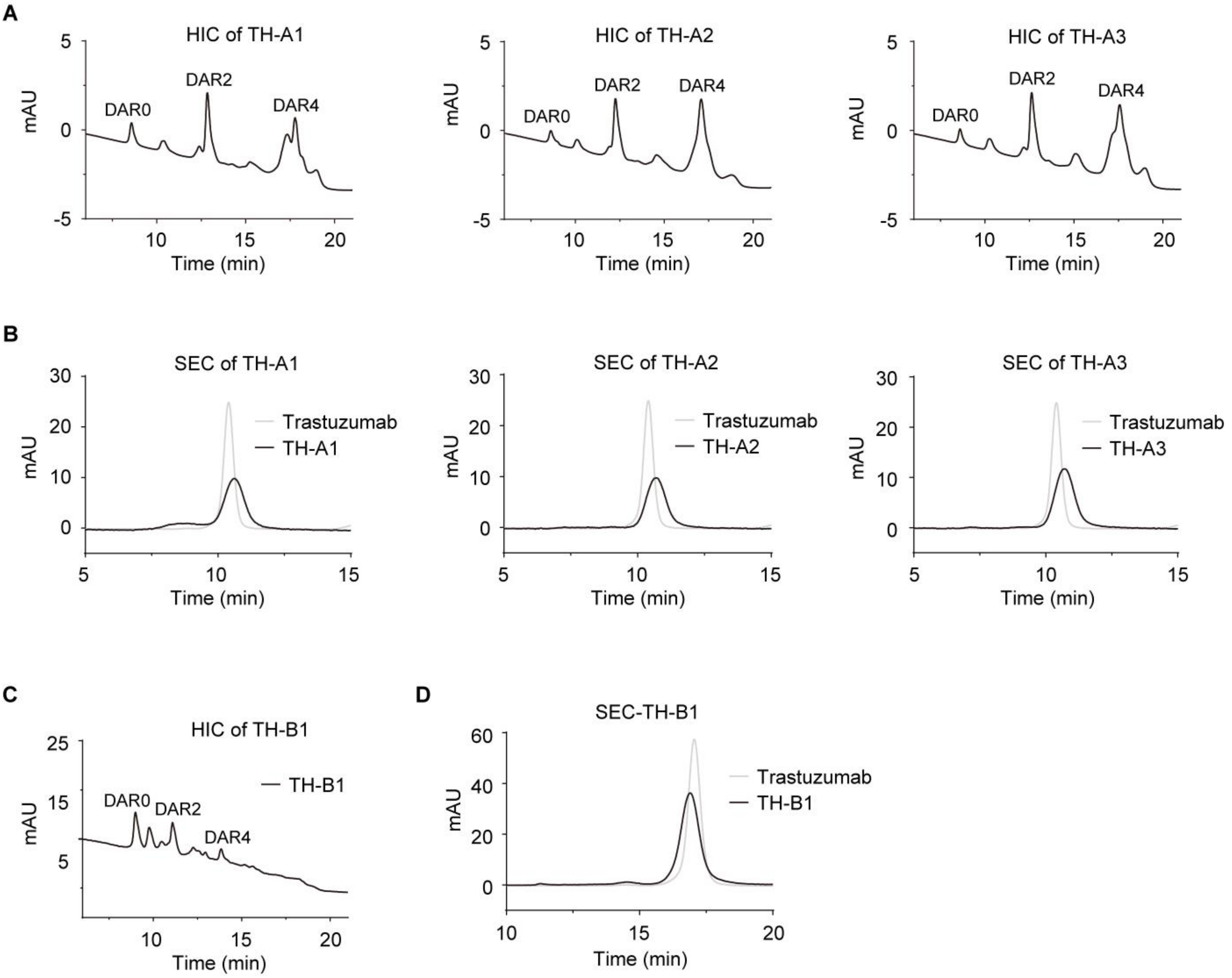


**Figure S2** The quality study of **TH-A1**–**A3**, and **TH-B1**–**B2**. The drug–antibody ratio (DAR) of ADCs was determined by hydrophobic chromatography (HIC), and the aggregation ratios % of ADCs were determined by size exclusion chromatography (SEC). (A) The DAR of **TH-A1**–**A3**. (B) The aggregation ratios % of **TH-A1**–**A3**. (C) The DAR of **TH-B1**. (D) The aggregation ratios % of **TH-B1**.

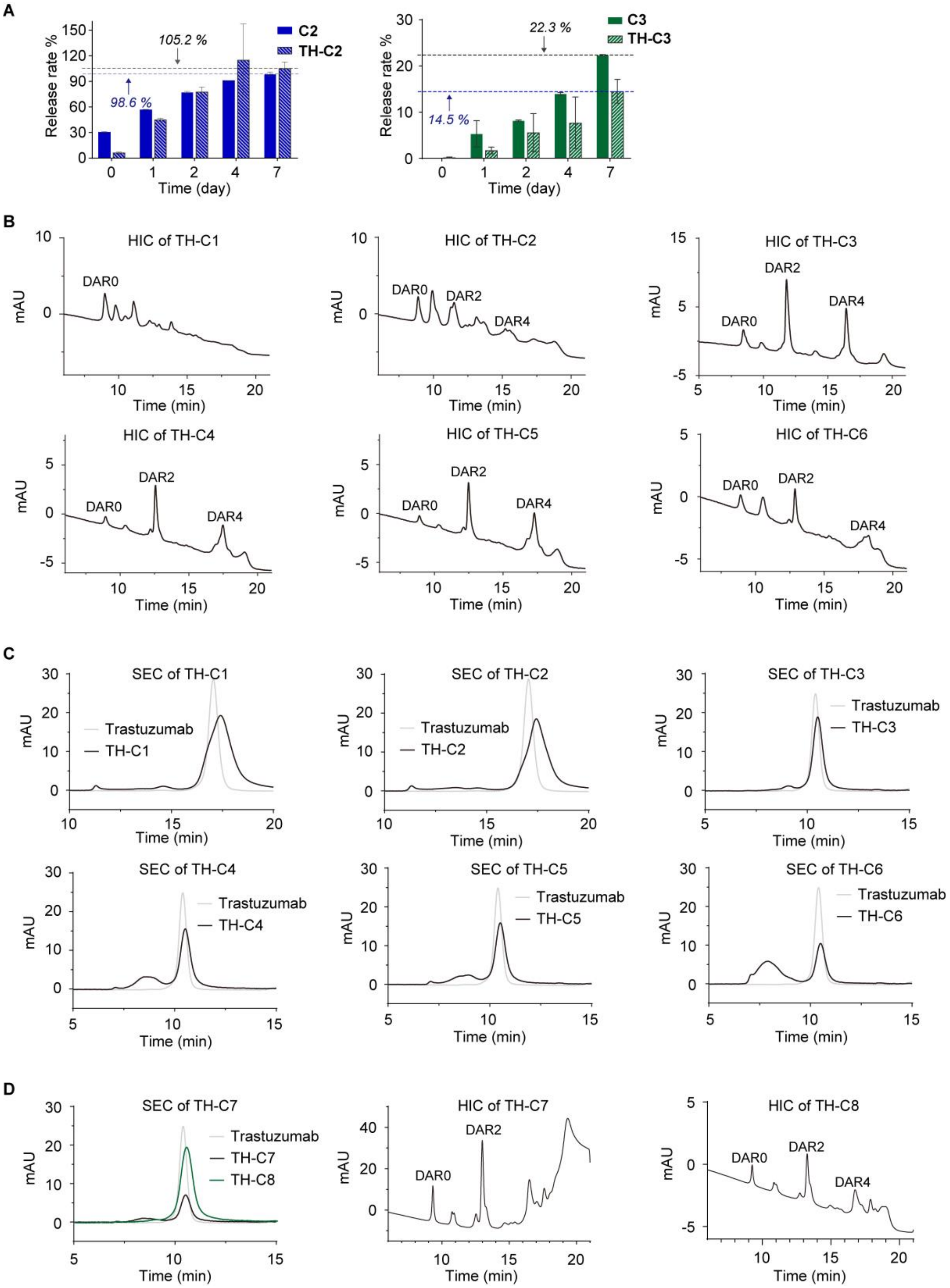


**Figure S3** The quality study of **TH-C1**–**C8**. (A) ADCs showed similar stability trends as linkers. (B) The DAR of **TH-C1**–**C6**. (C) The aggregation level of **TH-C1**–**C6**. (D) The quality study of **TH-C7** and **TH-C8**.

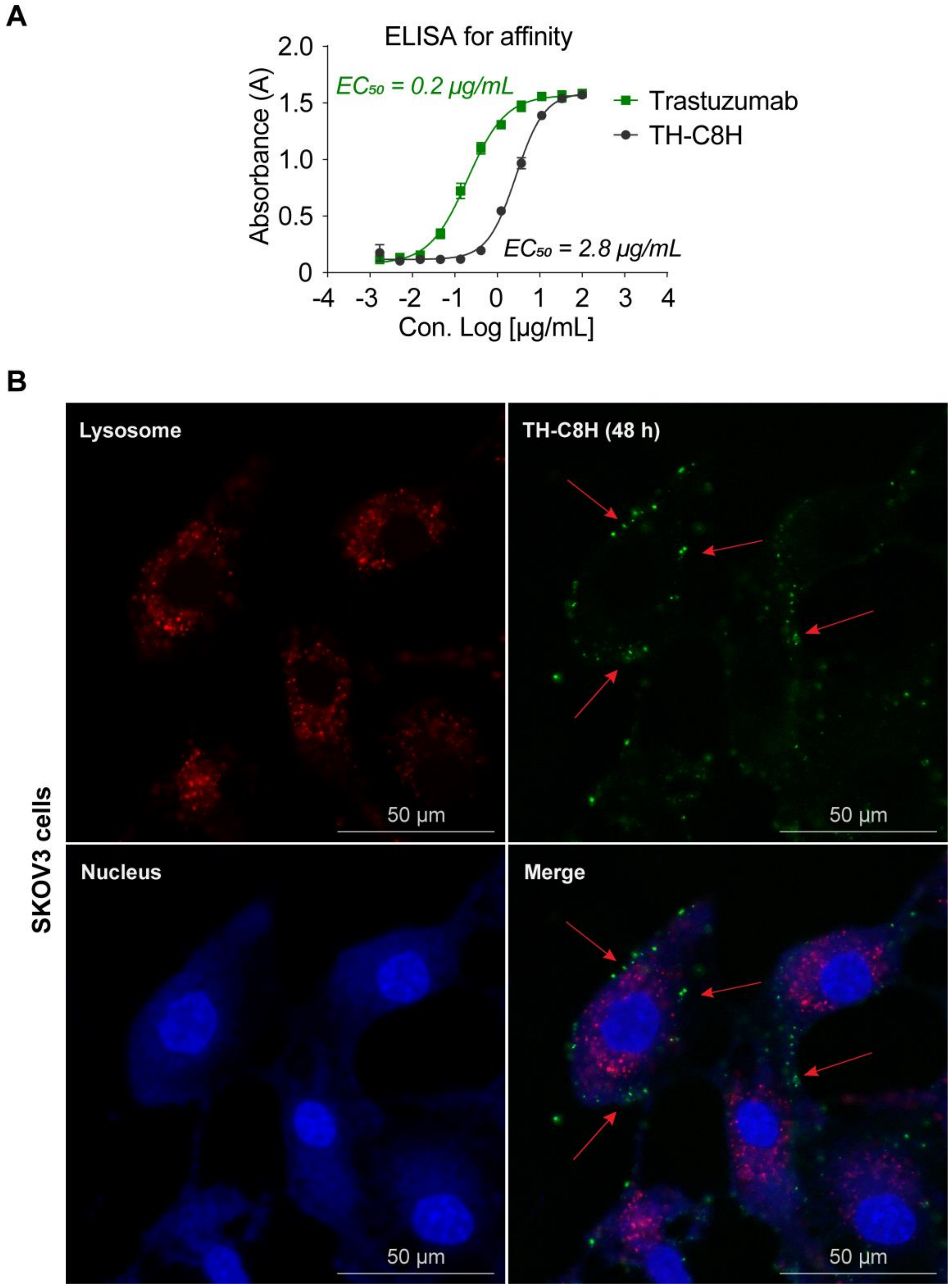


**Figure S4** The quality study of **TH-C8H**. (A) The binding affinities of **TH-C8H** and Trastuzumab for HER2 antigen were detected by ELISA. (B) Cell image analysis of **TH-C8H**. Image analysis was performed with a drug (FITC labeled **TH-C8H**) concentration of 1 μmol/L at 37 °C for 2 days. Scale bar = 50 μm.

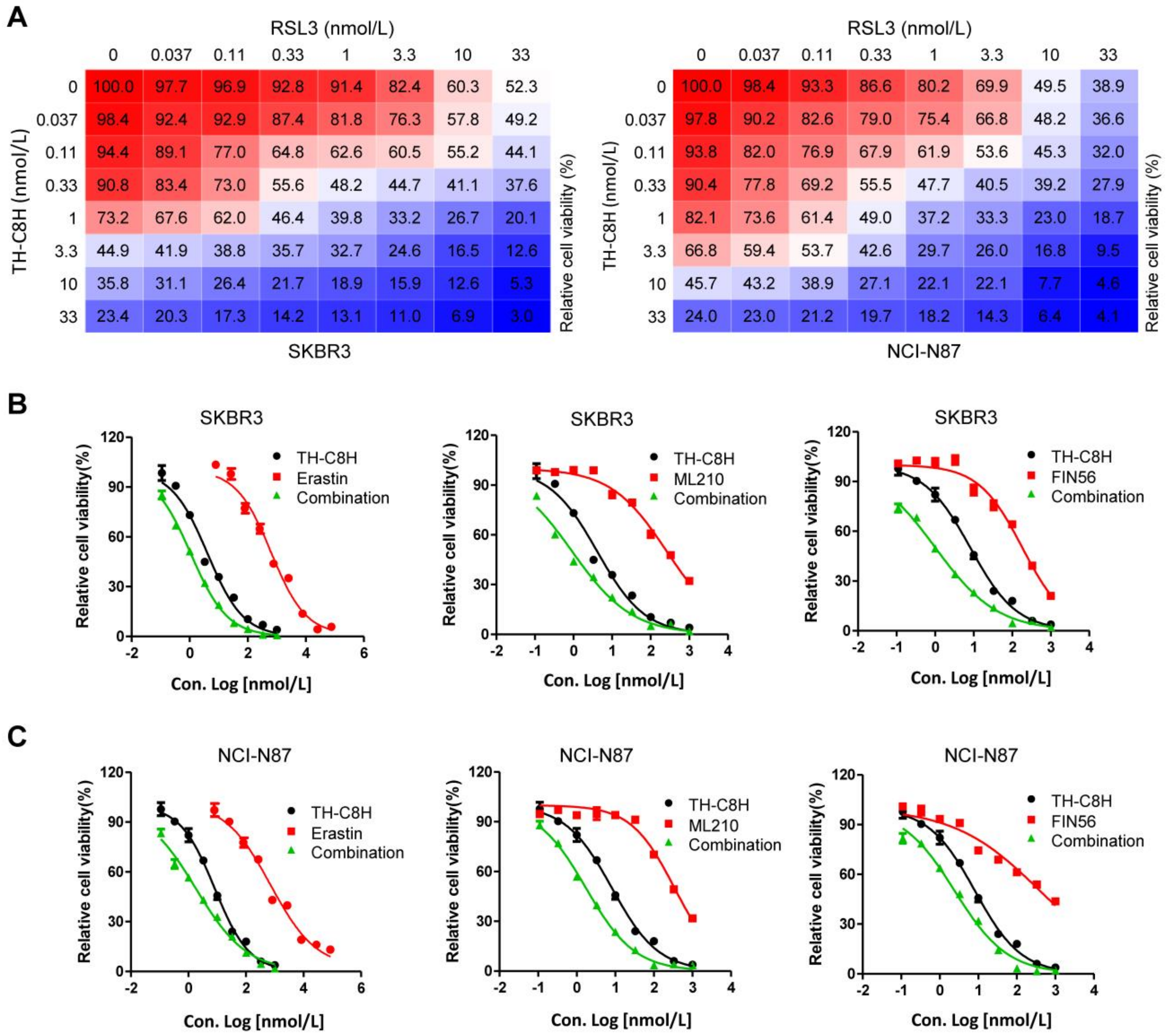


**Figure S5** Synergy of **TH-C8H** with ferroptosis inducers significantly inhibited HER2$^{+}$ cell growth. (A) Dose-response matrix for **TH-C8H** and **RSL3** combination in SKBR3 and NCI-N87 cells. Cell viability was measured 72 h after treatment with the indicated concentrations of drugs. (B, C) Effects of **TH-C8H** with **Erastin**, **ML210** or **FIN56** as single agents or drug combinations in SKBR3 cells (B) and NCI-N87 cells (C). Cell viability was measured after 96 h of treatment with the indicated drug dose. For (B), and (C), data are shown as mean ± SD ($n \geq 3$).

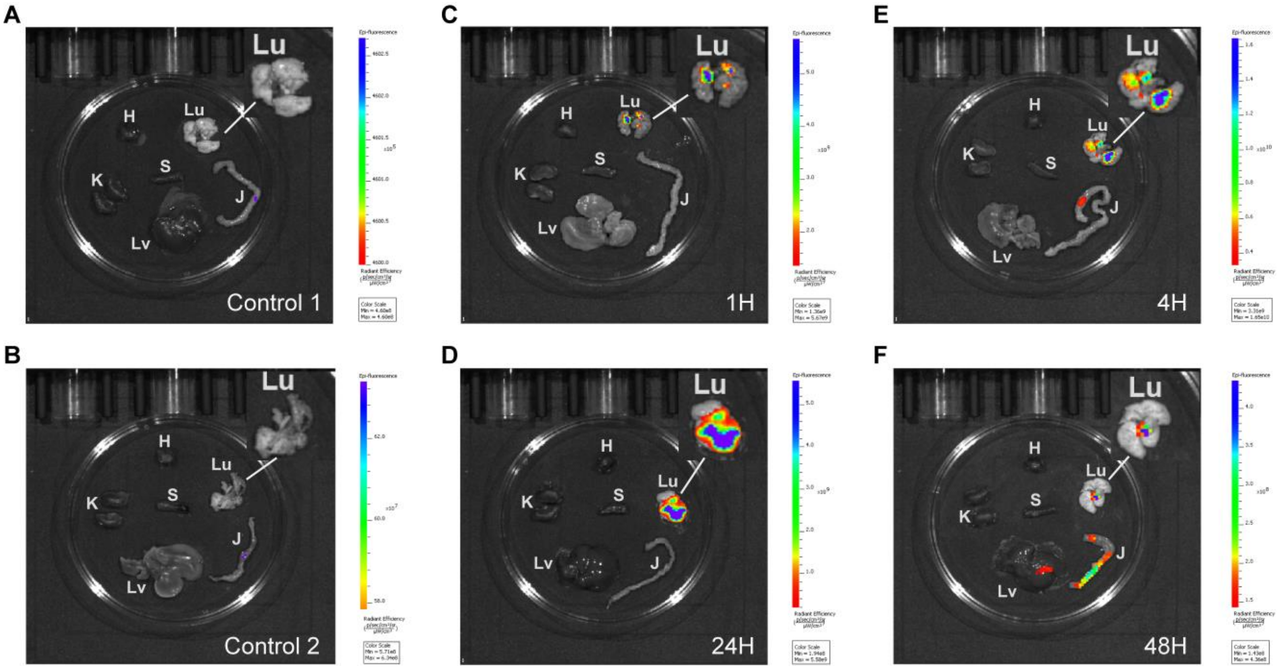


**Figure S6** The distribution of the antiviral ADC **HG-C3** after administration. The DyLight–680 labeled **HG-C3** was administered *via* nasal drops. Fluorescence at a wavelength of 680 nanometers was observed in the lungs within 1 h after administration and lasted for at least 48 h.

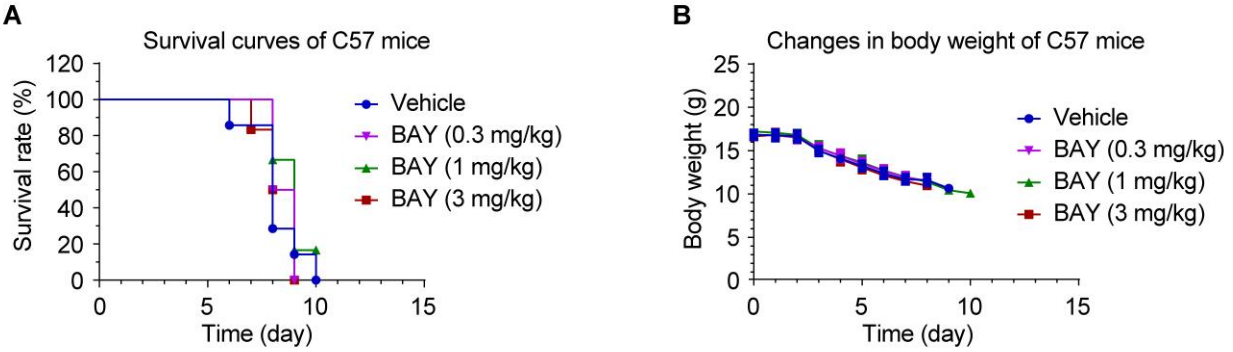


**Figure S7** *In vivo* anti-influenza virus (CA07 mutant) efficacy of **BAY** (intraperitoneal injection). (A) Survival curves of C57 mice in different dosing groups ($n \geq 6$). (B) Changes in body weight of C57 mice in different dosing groups.

## 2. HR-MS and $^{1}$H-NMR of linkers (A1–A3, B1–B2, C1–C8)

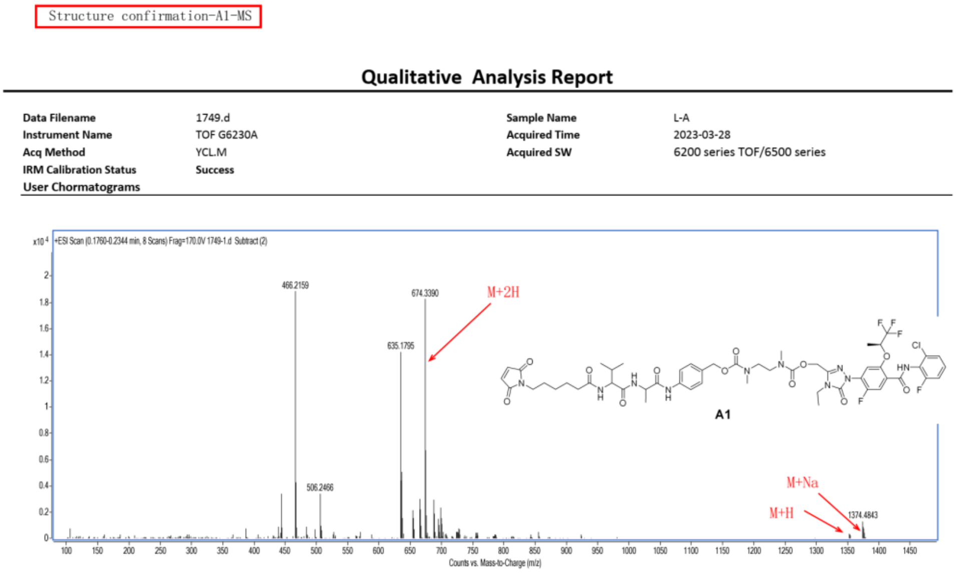


HR-MS of **A1**

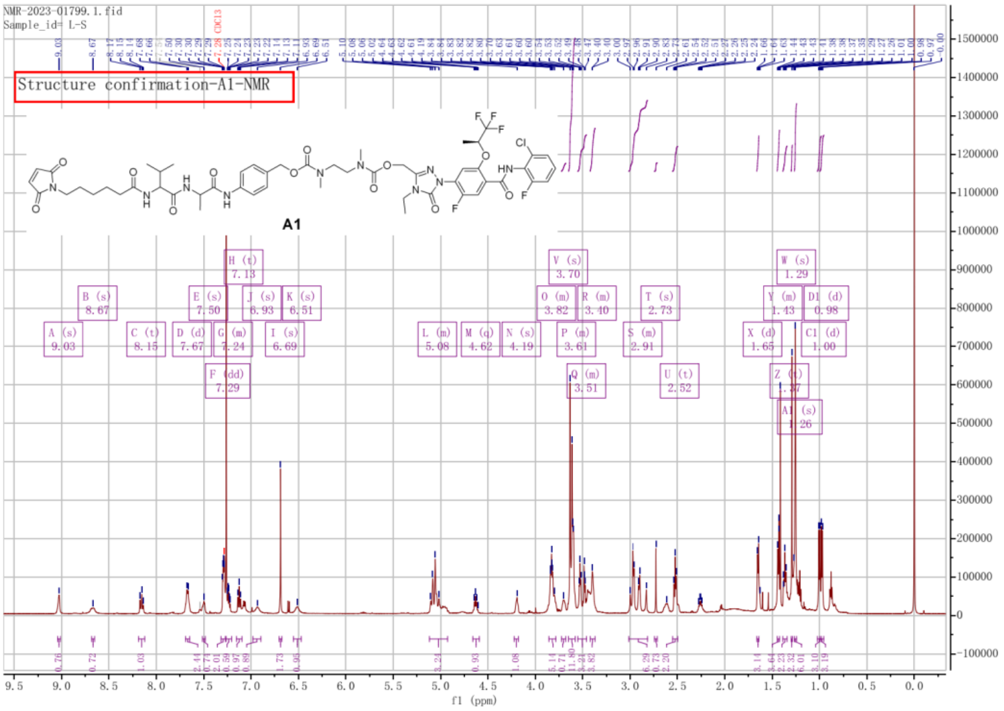


$^{1}$H-NMR of **A1**

Structure confirmation-A2-MS

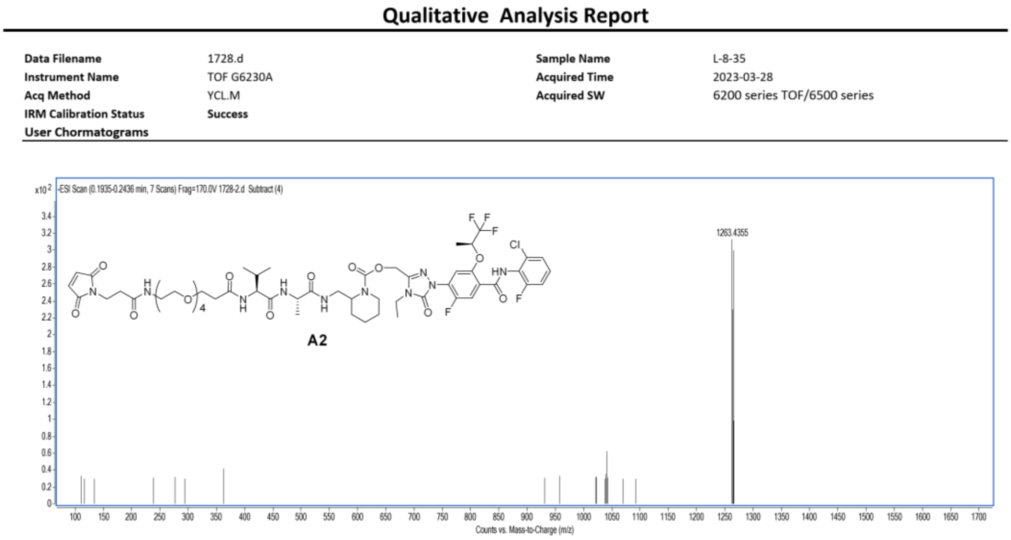


HR-MS of **A2**

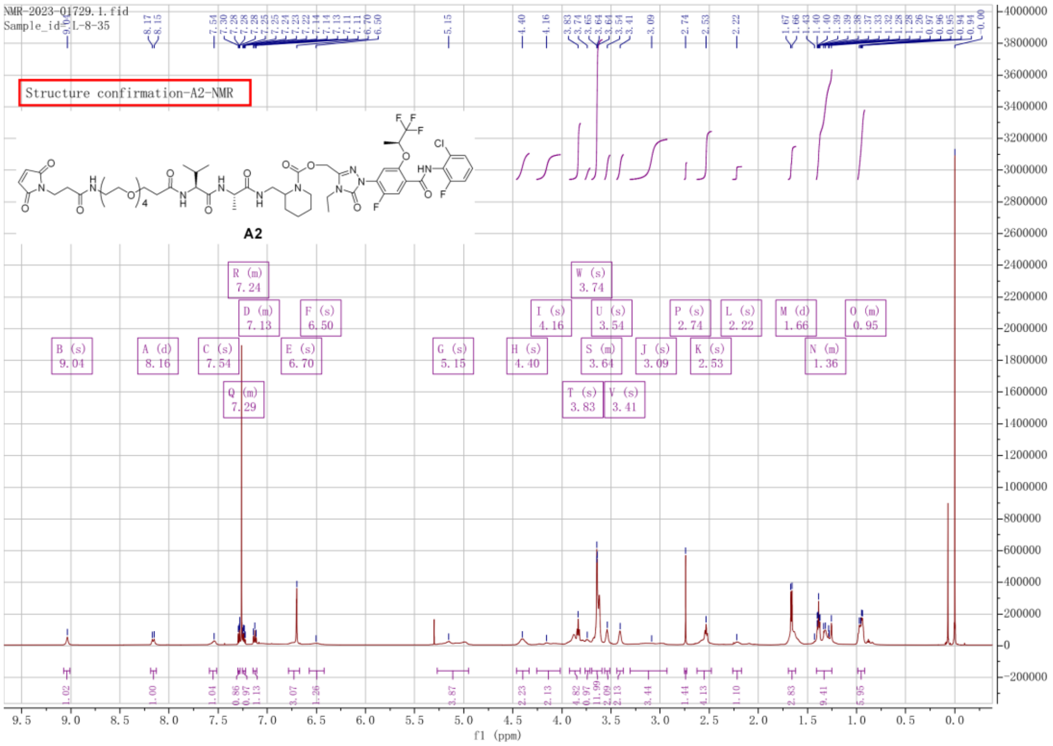


$^{1}$H-NMR of **A2**

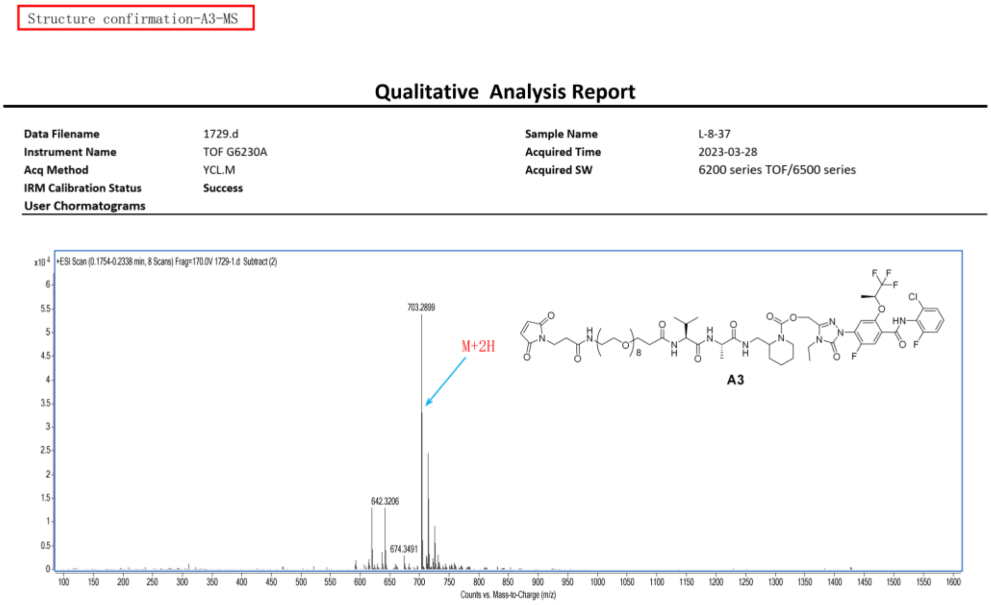


HR-MS of **A3**

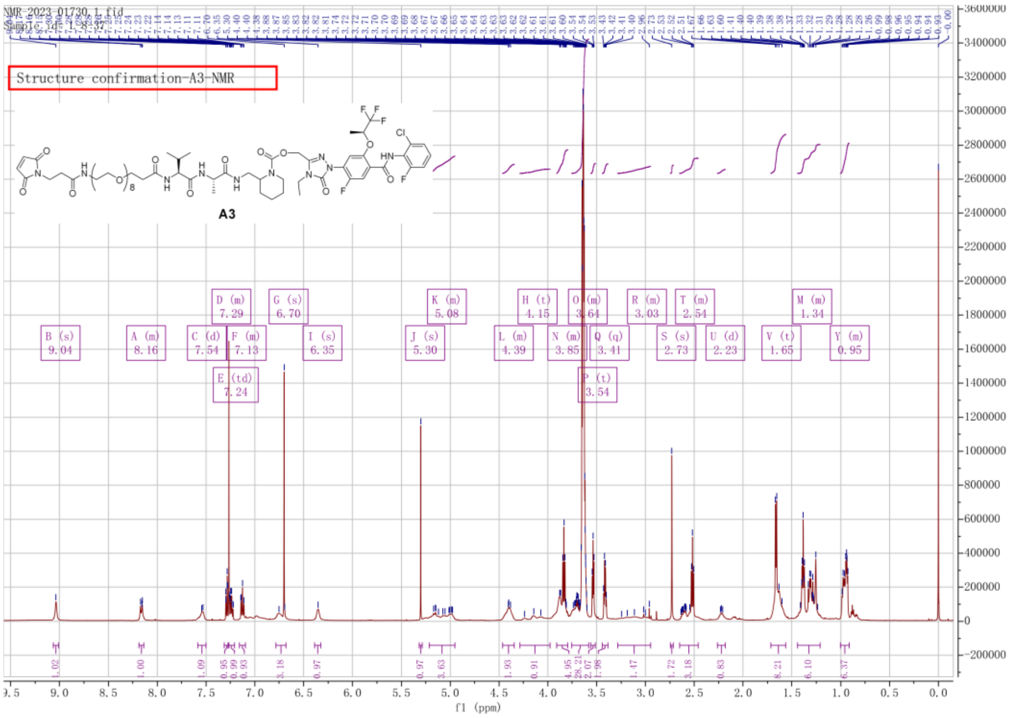


$^{1}$H-NMR of **A3**

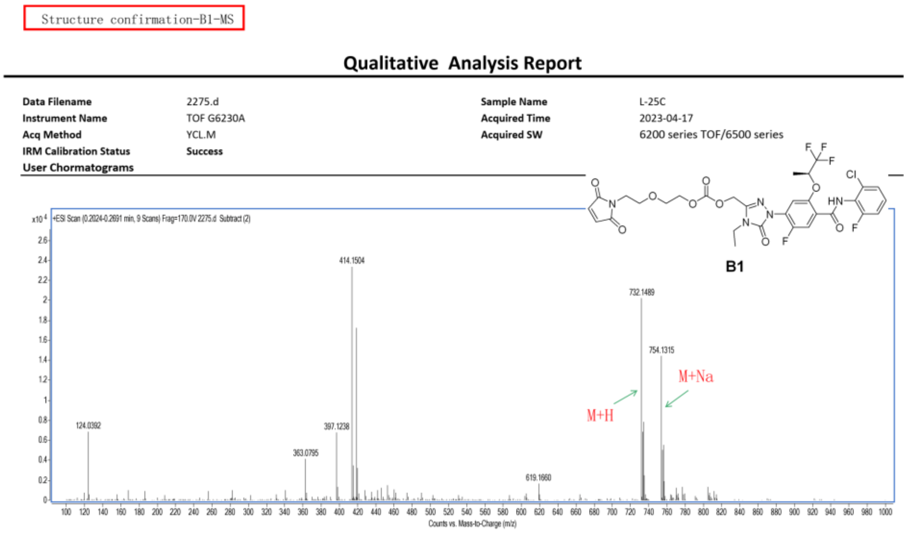


HR-MS of **B1**

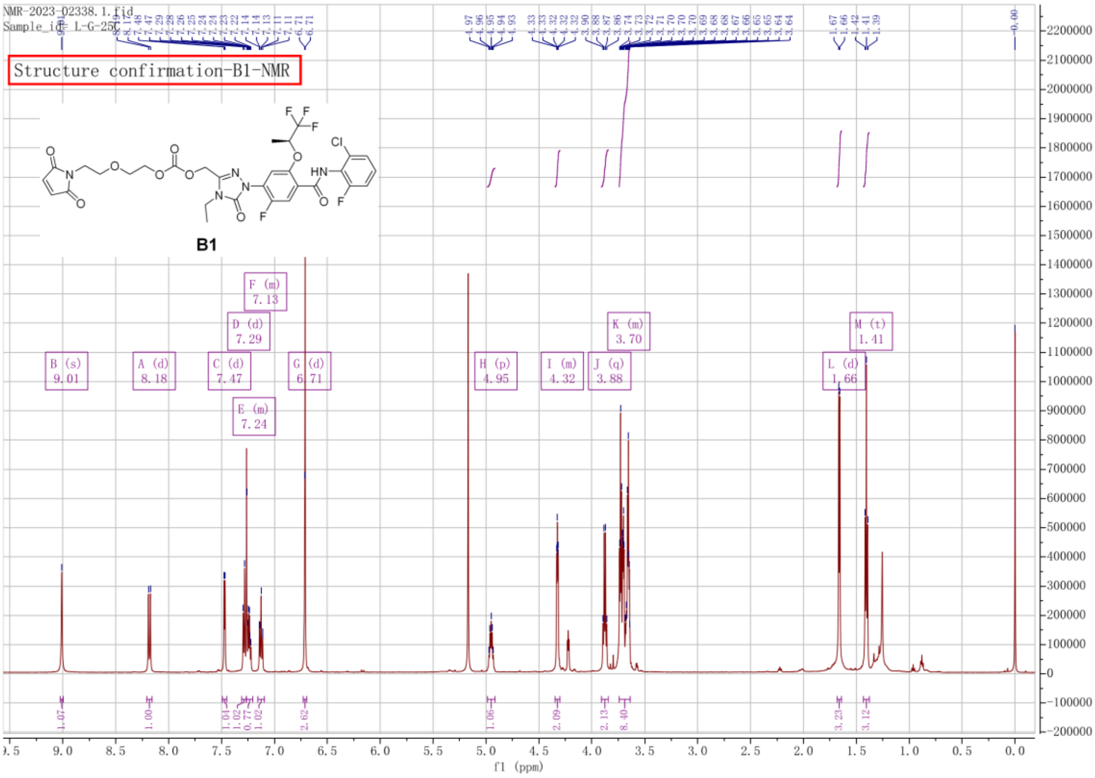


$^{1}$H-NMR of **B1**

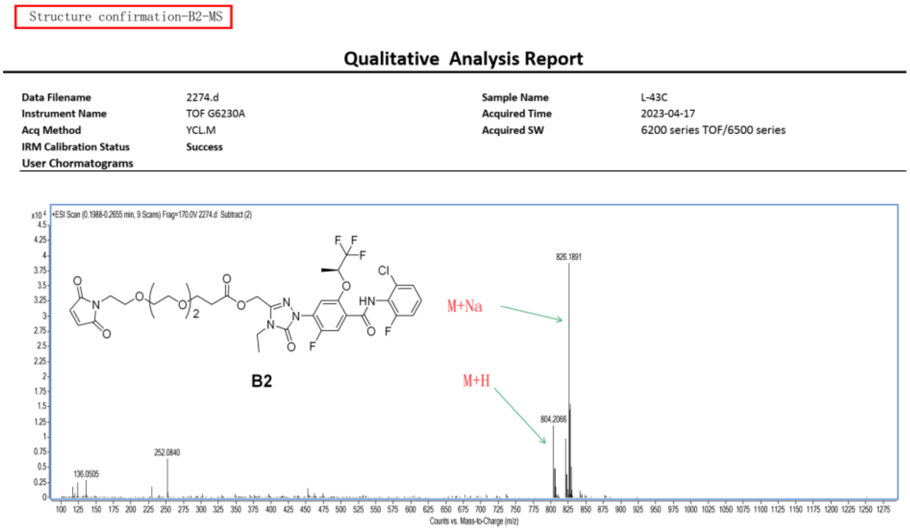


HR-MS of **B2**

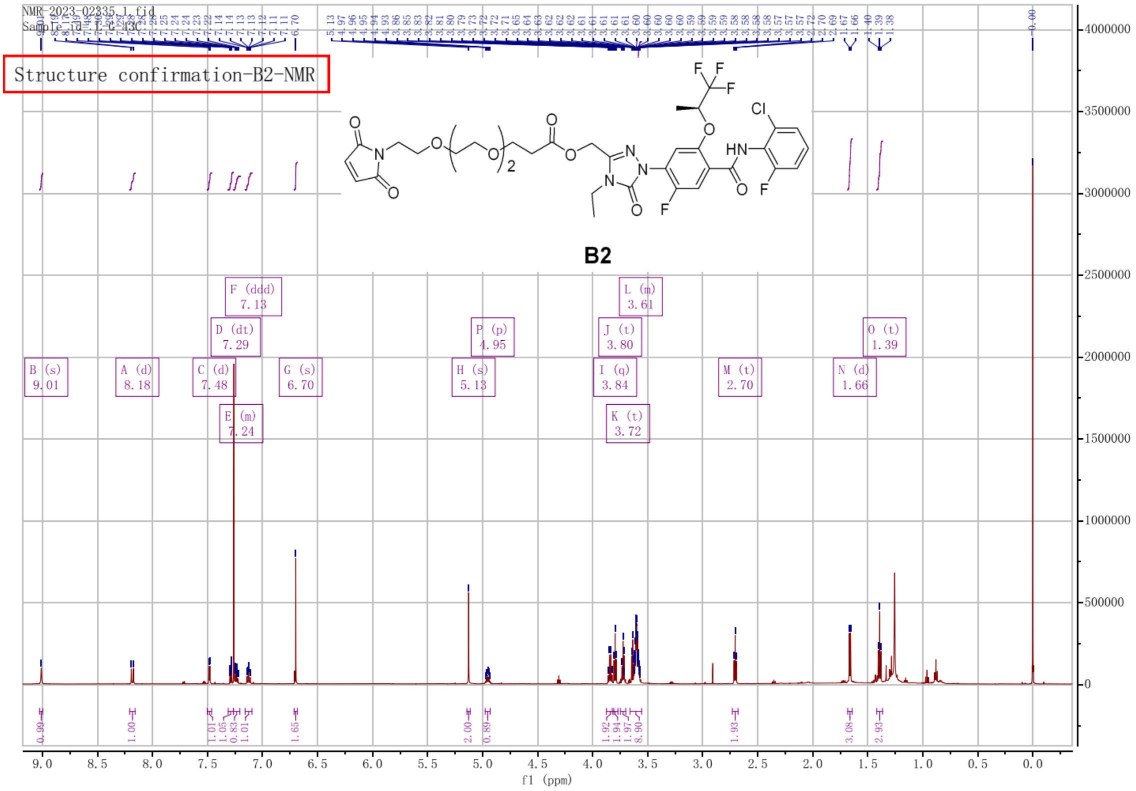


$^{1}$H-NMR of **B2**

Structure confirmation-C1-MS

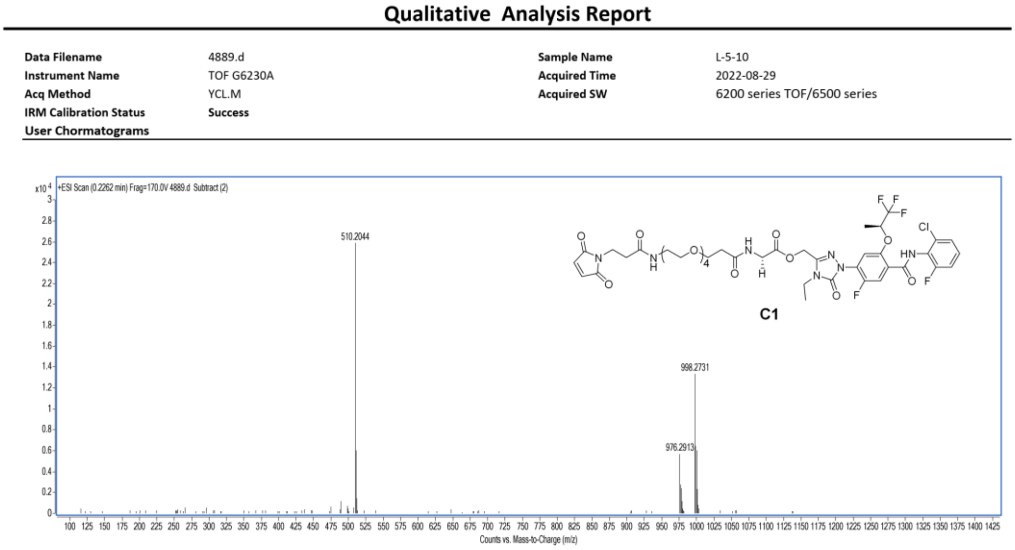


HR-MS of **C1**

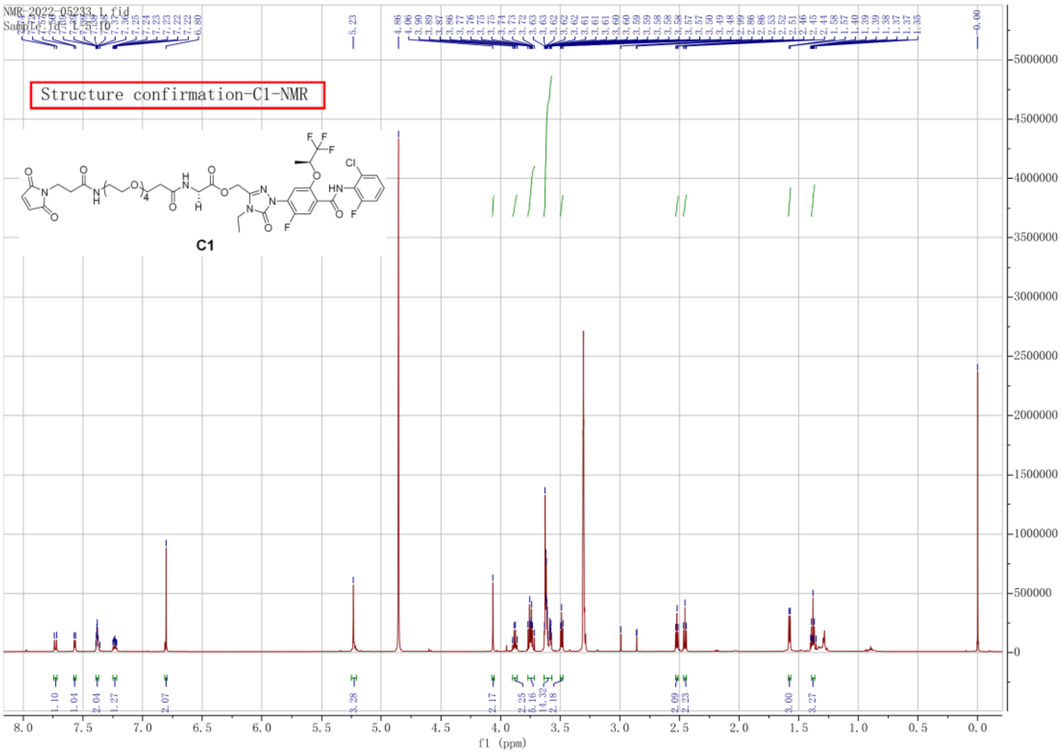


$^{1}$H-NMR of **C1**

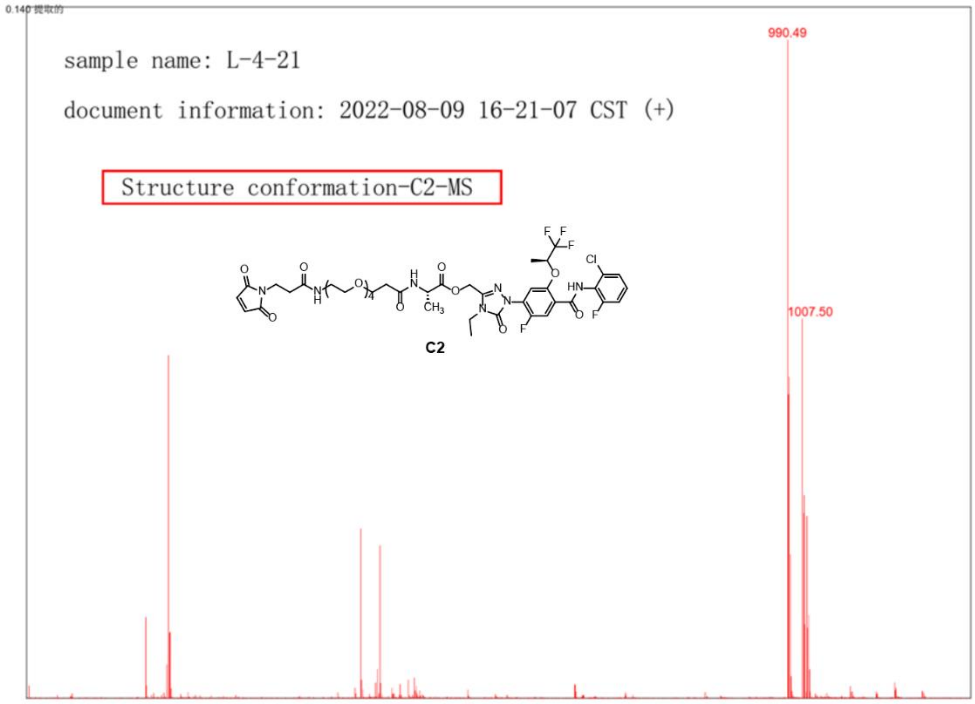


HR-MS of **C2**

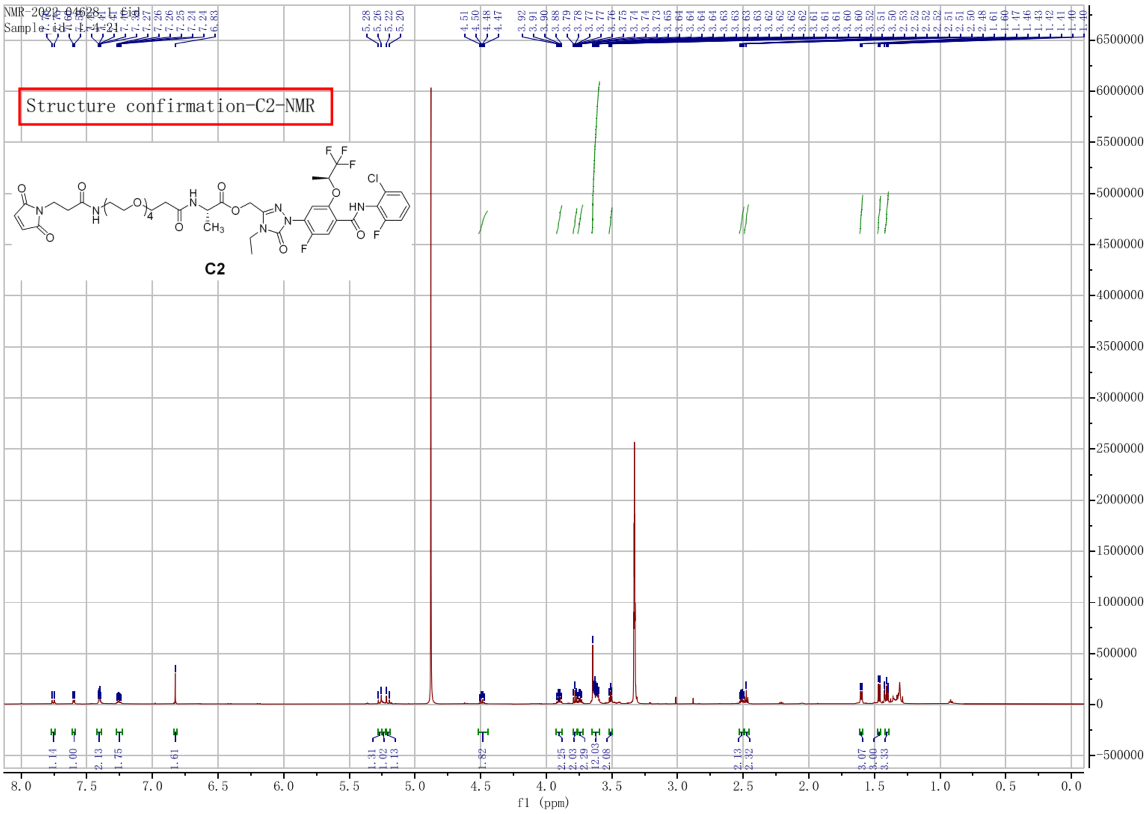


$^{1}$H-NMR of **C2**

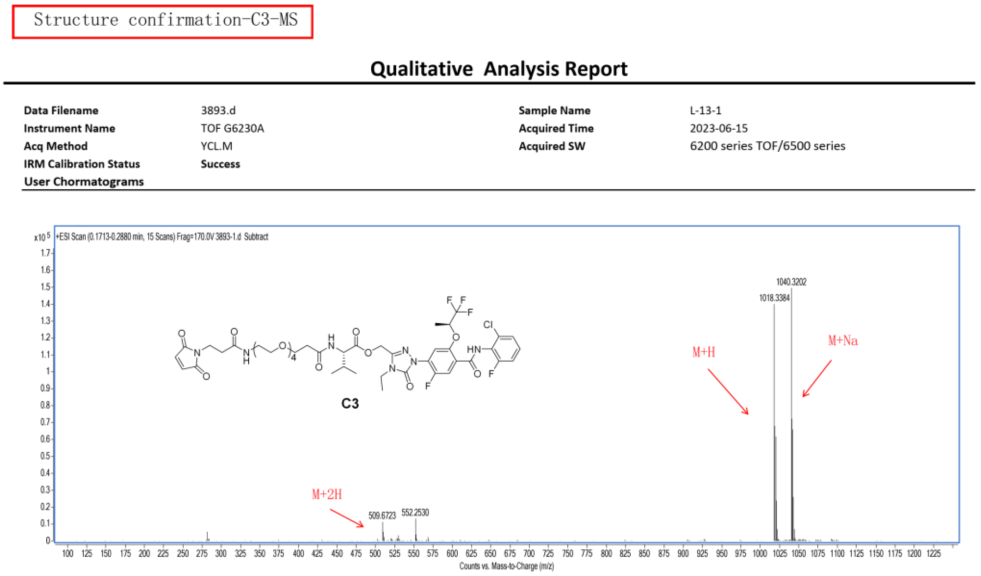


HR-MS of **C3**

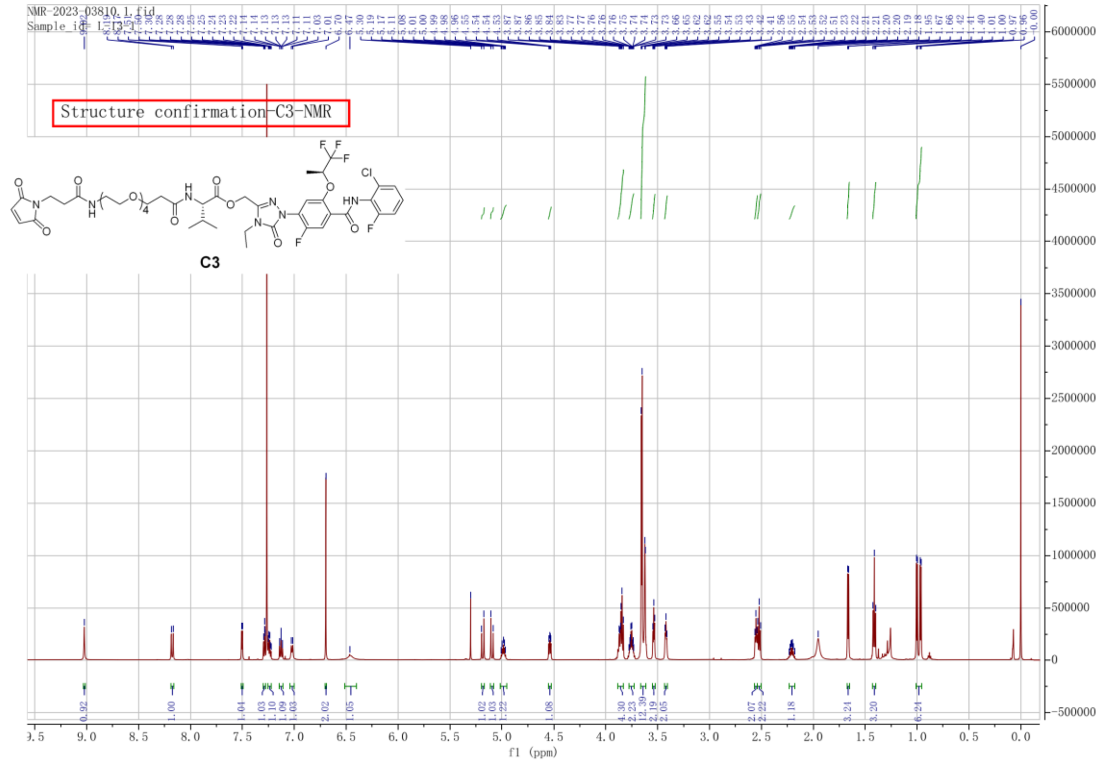


$^{1}$H-NMR of **C3**

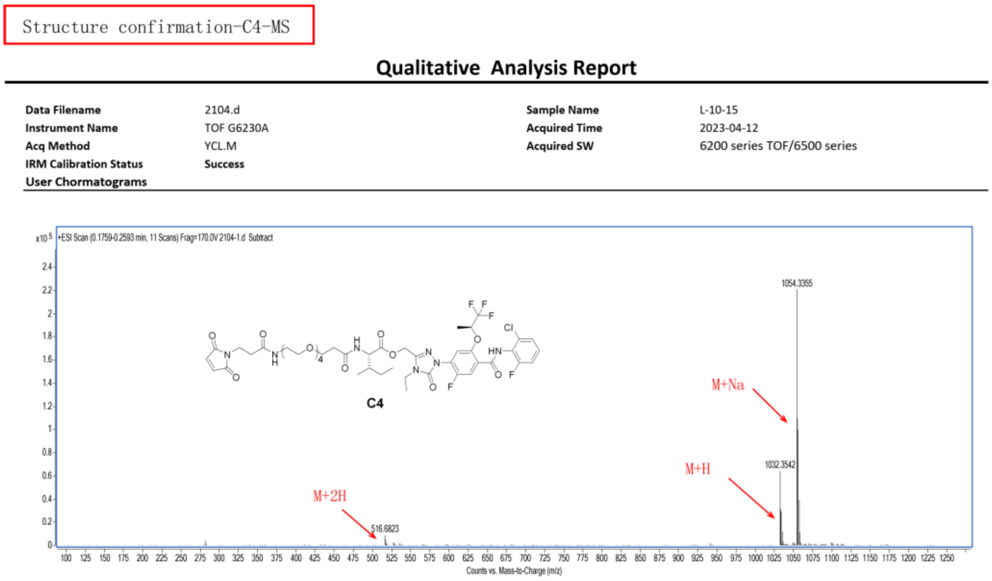


HR-MS of **C4**

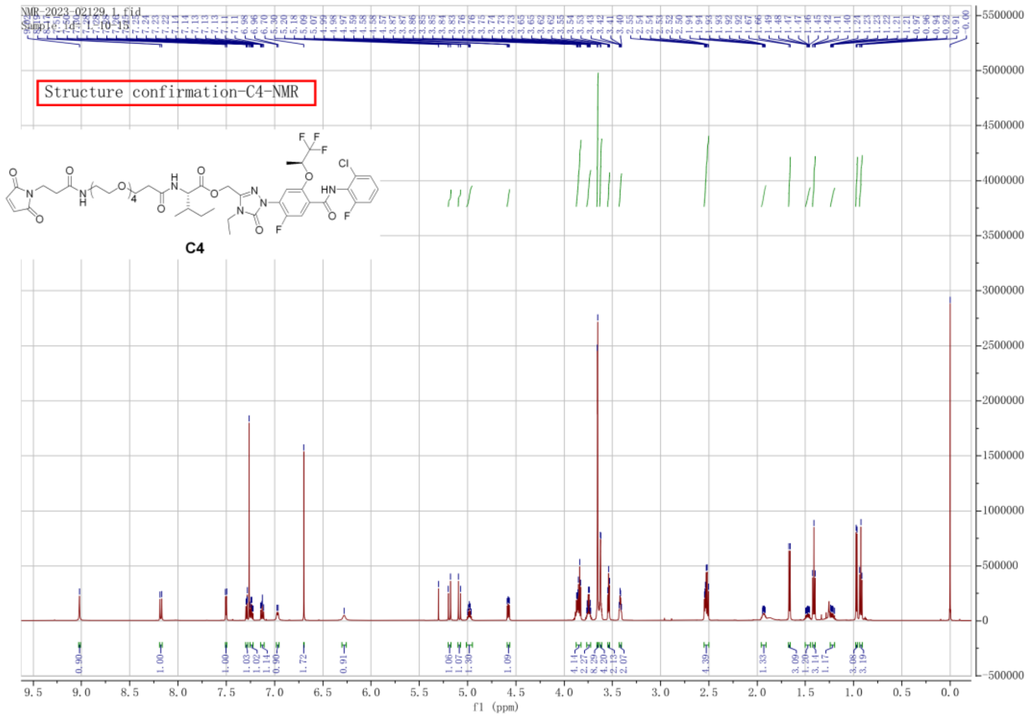


$^{1}$H-NMR of **C4**

Structure confirmation-C5-MS

## Qualitative Analysis Report

| | | | |
|---|---|---|---|
| **Data Filename** | 2107.d | **Sample Name** | L-10-17 |
| **Instrument Name** | TOF G6230A | **Acquired Time** | 2023-04-12 |
| **Acq Method** | YCL.M | **Acquired SW** | 6200 series TOF/6500 series |
| **IRM Calibration Status** | Success | | |
| **User Chormatograms** | | | |

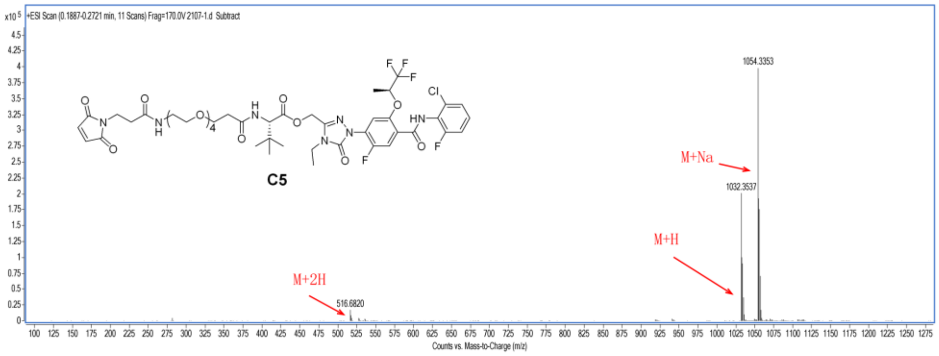


HR-MS of **C5**

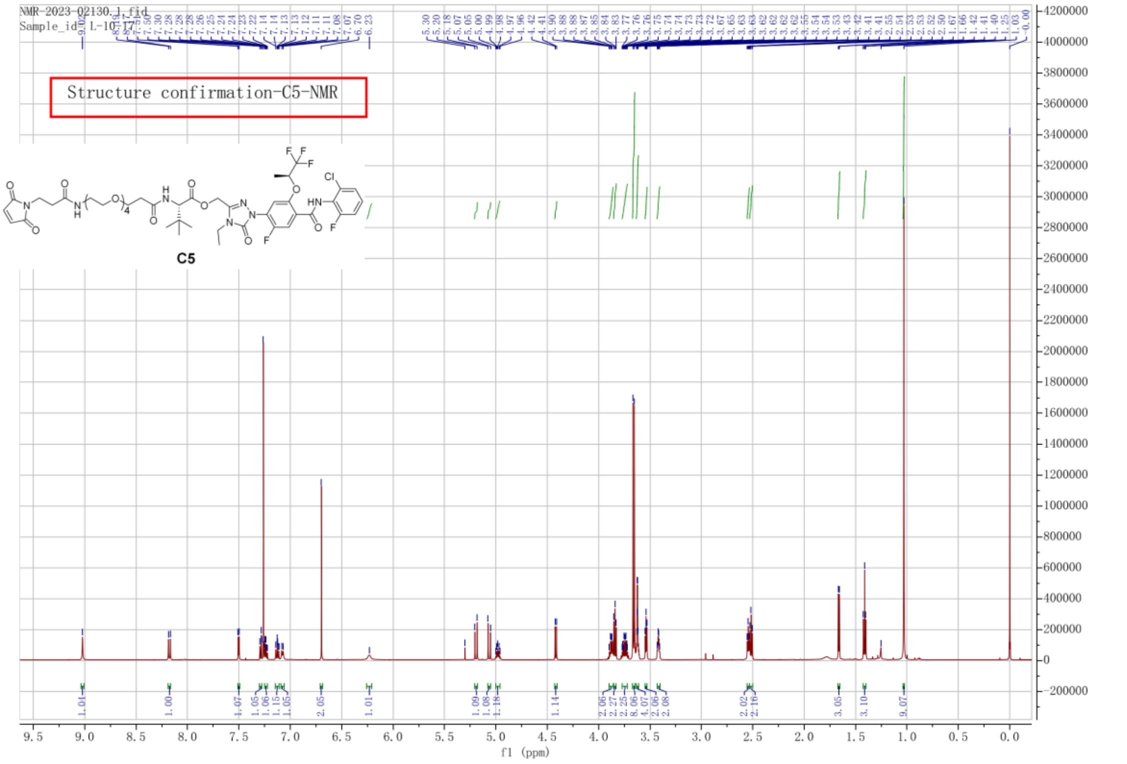


$^{1}$H-NMR of **C5**

Structure confirmation-C6-MS

## Qualitative Analysis Report

| | | | |
|---|---|---|---|
| **Data Filename** | 2109.d | **Sample Name** | L-10-19 |
| **Instrument Name** | TOF G6230A | **Acquired Time** | 2023-04-12 |
| **Acq Method** | YCL.M | **Acquired SW** | 6200 series TOF/6500 series |
| **IRM Calibration Status** | Success | | |
| **User Chormatograms** | | | |

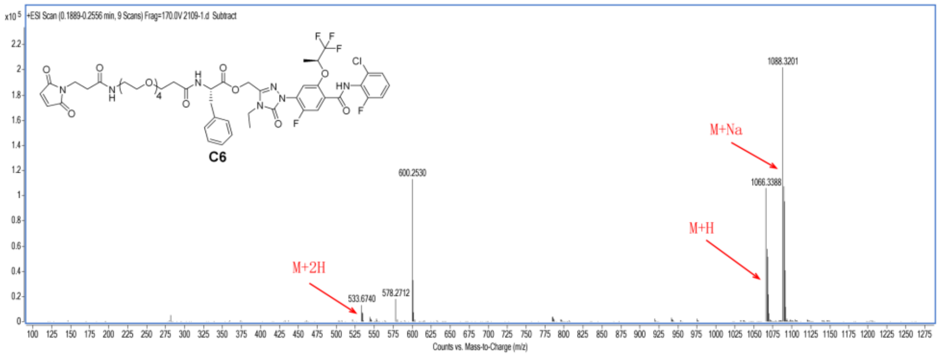


HR-MS of **C6**

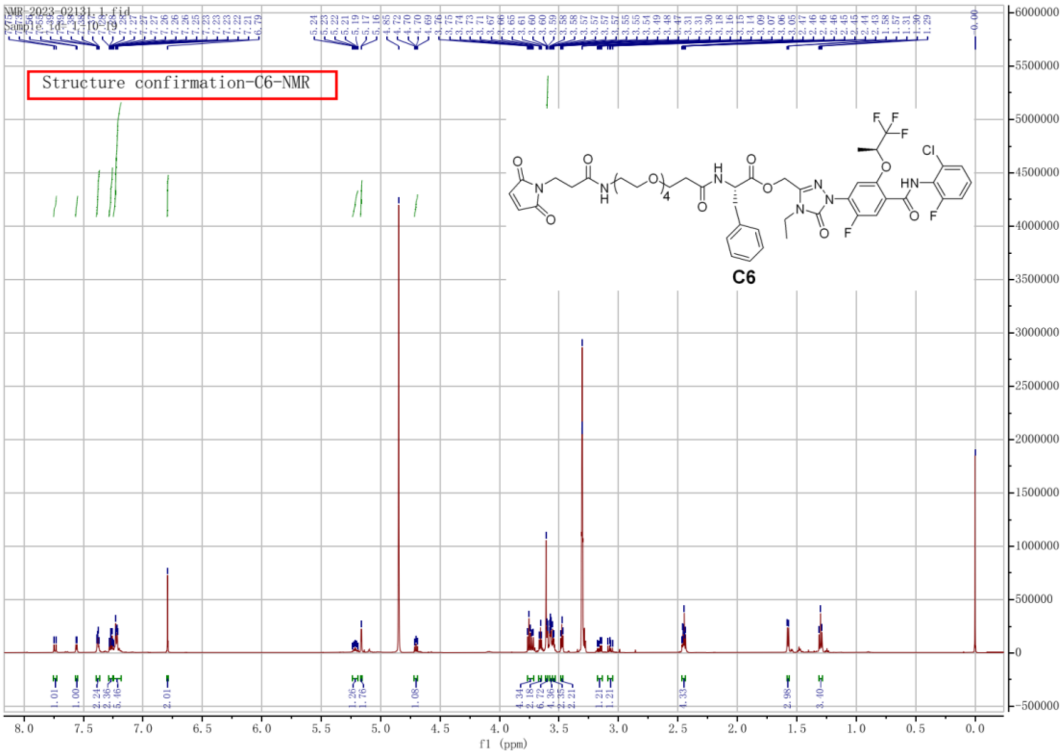


$^{1}$H-NMR of **C6**

Structure confirmation-C7-MS

## Qualitative Analysis Report

| | | | |
|---|---|---|---|
| **Data Filename** | 2791.d | **Sample Name** | L-12-5 |
| **Instrument Name** | TOF G6230A | **Acquired Time** | 2023-05-06 |
| **Acq Method** | YCL.M | **Acquired SW** | 6200 series TOF/6500 series |
| **IRM Calibration Status** | **Success** | | |
| **User Chormatograms** | | | |

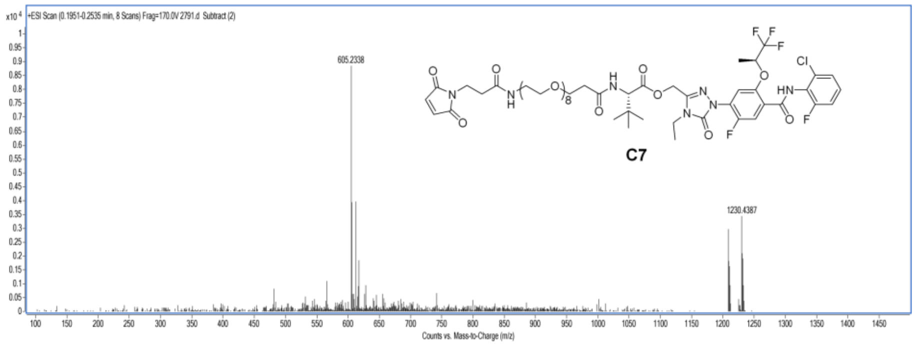


HR-MS of **C7**

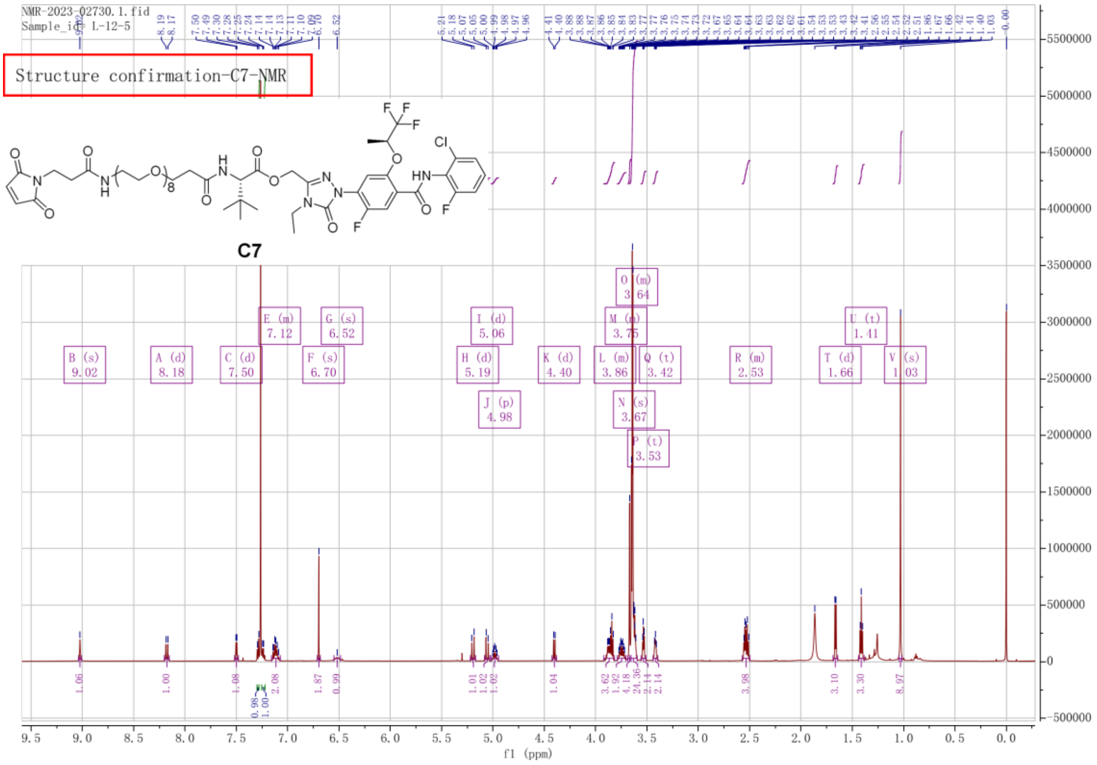


$^1$H-NMR of **C7**

Structure confirmation-C8-MS

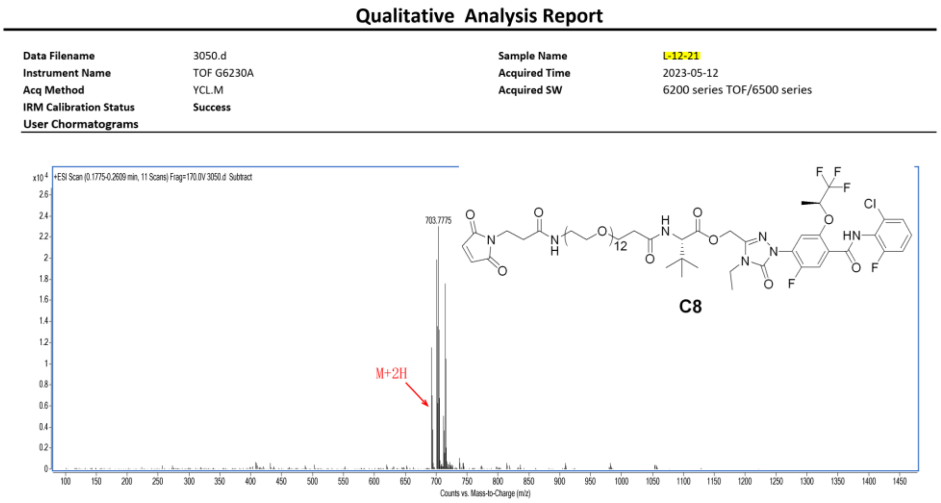


HR-MS of **C8**

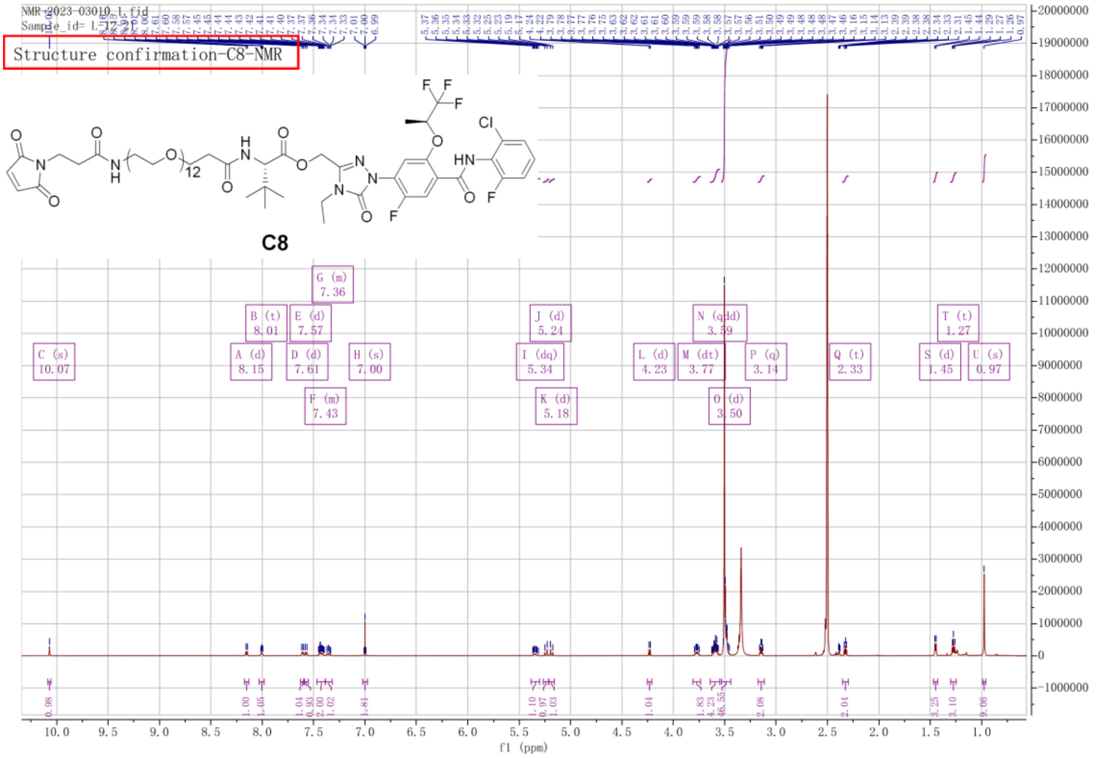


$^{1}$H-NMR of **C8**
